# Surface reduction boosts free electron concentration in MXene for enhanced photothermal performance

**DOI:** 10.1126/sciadv.aee2009

**Published:** 2026-05-13

**Authors:** Haoming Ding, Xiao Tong, Yong Zhang

**Affiliations:** ^1^ Department of Biomedical Engineering, College of Biomedicine, City University of Hong Kong, Hong Kong SAR, China.; ^2^ Cardiovascular and Cerebrovascular Health Research Centre (COCHE), Hong Kong Science Park, Hong Kong SAR, China.; ^3^ Department of Materials Science and Engineering, College of Engineering, City University of Hong Kong, Hong Kong SAR, China.

## Abstract

The photothermal properties of MXenes originate from the localized surface plasmon resonance, which is attributed to their high free electron concentration. However, their intrinsic electron concentration is limited by suboptimal d-orbital occupancy and the electron-withdrawing effect from electronegative terminations. Herein, we report a sodium-mediated surface reduction strategy in molten salts, which optimizes the surface coordination environment via electronic modulation to mitigate the electron-withdrawing and electron-scattering effects, while simultaneously injecting electrons into the Ti-3d states for controlled state filling. This dual modulation enables the free electron concentration, carrier mobility, and electrical conductivity to reach 4.92, 2.63, and 12.96 times that of the pristine MXene, respectively. The optimized reduced Ti_3_C_2_ achieves a high photothermal conversion efficiency of 91.66% under 808-nanometer laser irradiation. A photothermal antibacterial woundplast with ultralow MXene content demonstrates a high bacterial kill rate. This work not only shows an effective method for tuning the photothermal properties of MXenes but also inspires applications that require tailored surface chemistry and high electron concentration.

## Teaser

A surface reduction strategy for boosting free electron concentration of MXene.

## INTRODUCTION

MXenes represent an emerging class of two-dimensional (2D) transition metal carbides and nitrides, typically synthesized by selectively etching the A layer (e.g., etching Al from Ti_3_AlC_2_) using different etchants [e.g., hydrofluoric acid (HF) and Lewis acidic molten salts] (*1*–*5*). This process yields materials with the general formula M_*n*+1_X*_n_*T*_x_*, where M is an early transition metal (e.g., Ti, V, Nb, and Mo), X is carbon/nitrogen, and T*_x_* denotes surface terminations (-O, -OH, -F, and -Cl) inevitably introduced during synthesis or subsequent processing (*2*, *5*). This unique structure endows MXenes with exceptional metallic conductivity, mechanical flexibility, hydrophilicity, and tunable electronic properties, driving application exploration across energy storage, catalysis, and biomedicine (*5*–*8*). For biomedical applications, MXenes have attracted considerable attention due to their photothermal conversion capabilities, especially in the near-infrared (NIR) biological window (*9*–*13*). This capability is primarily driven by localized surface plasmon resonance (LSPR), a phenomenon arising from the oscillation of free electrons in the conductive MXene layers when excited by NIR photons (*14*, *15*). The d-orbital electrons of transition metals endow MXenes with a high density of states (DOS), which is essential for robust LSPR, thereby establishing the free electron concentration as a pivotal factor governing photothermal conversion efficiency (PTCE) (*16*, *17*). Currently, the PTCE of MXenes is still low, which limits their application in the biomedical field (*12*). Given that, many efforts have been made to improve their photothermal performance through structural and electronic engineering strategies (*14*, *18*–*20*).

Strategies to improve the photothermal performance of MXenes include decorating the MXene surface with plasmonic noble metal nanoparticles (e.g., Au, Ag, and Pt) to enhance NIR absorption (*18*). However, this approach introduces complexity in synthesis and raises concerns about nanoparticle stability and biocompatibility. Other approaches include intrinsic electron modulation, such as nitrogen doping in Ti_3_C_2_ to increase DOS via additional electron donation, or designing high-entropy MXenes to induce d-orbital hybridization and delocalize electrons (*19*, *20*). A more direct approach is surface chemistry regulation, as electronegative terminations withdraw electrons from the M_*n*+1_X*_n_* lattice to suppress free electron concentration (*21*–*23*). Thus, it is reasonable to tailor the terminations’ electronegativity or to reduce the amount of terminations for restoring intrinsic DOS and PTCE (*23*, *24*). Conventional high-temperature annealing can only remove weakly bound surface terminations (e.g., -F and -OH), while chemical reduction with sodium hydride (NaH), gallium (Ga), or hydrogen (H_2_) usually results in incomplete termination removal or 2D lattice destruction (*21*, *25*–*28*). On the other side, because the d orbitals of the M atoms in MXenes are not filled, external electron injection may substantially increase the electron concentration, which is a notable advantage over other 2D materials. However, to the best of our knowledge, this electron injection process has not yet been demonstrated.

Here, we report a sodium (Na)–mediated molten salt reduction strategy that achieves dual electronic optimization: (i) alleviating their electron-withdrawing and scattering effect by modifying the surface termination through the electronic regulation, and (ii) direct electron injection into the Ti-3d states of Ti_3_C_2_ via Na-induced Ti reduction. This synergistic process saturates Ti-3d states near the Fermi level and optimizes the surface coordination, resulting in free electron concentration, mobility, and conductivity reaching 4.92, 2.63, and 12.96 times that of pristine MXene, respectively. As a result, the optimized reduced Ti_3_C_2_ achieves an ultrahigh PTCE of 91.66% under 808-nm laser irradiation, which is the highest efficiency reported for MXenes at this wavelength to the best of our knowledge. As a proof of concept for chronic wound treatment, a light-triggered woundplast with ultralow MXene content can effectively kill 91.39% of *Staphylococcus aureus*, highlighting the translational potential of this approach.

## RESULTS

### Synthesis of reduced Ti_3_C_2_ MXene

**Fig. 1.**
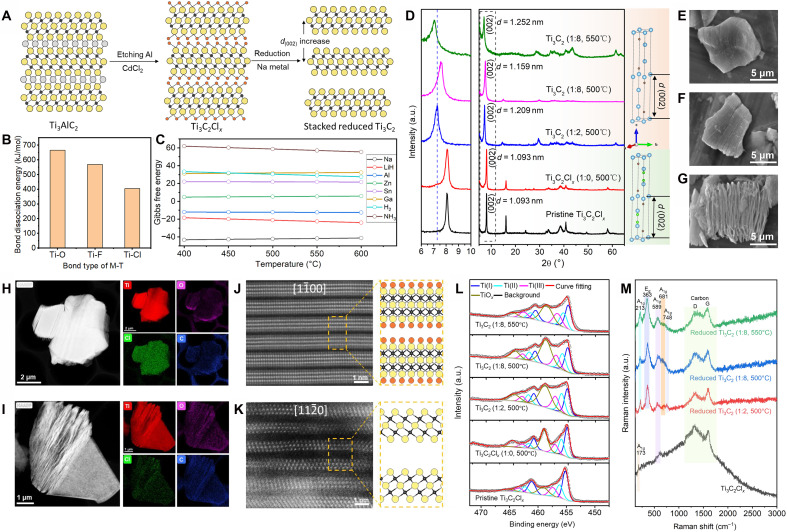
Synthesis of reduced MXene through a Na-mediated molten salt reduction strategy. (**A**) Schematic illustration of the synthesis process of reduced Ti_3_C_2_ MXenes. (**B**) Bond dissociation energy for Ti─O, Ti─F, and Ti─Cl bonds (*29*). (**C**) Gibbs free energy of reactions between Ti^3+^ and different reductants, including Na, LiH, Al, Zn, Sn, Ga, H_2_, and NH_3_. (**D**) X-ray diffraction (XRD) patterns of pristine Ti_3_C_2_Cl*_x_* MXene and reduced Ti_3_C_2_ MXenes synthesized under different reaction conditions. Scanning electron microscopy (SEM) image of pristine Ti_3_C_2_Cl*_x_* (**E**), annealed Ti_3_C_2_Cl*_x_* in molten salts (**F**), and reduced Ti_3_C_2_ (1:8, 550°C) (**G**). High-angle annular dark field (HAADF) transmission electron microscopy (TEM) image of pristine Ti_3_C_2_Cl*_x_* (**H**) and reduced Ti_3_C_2_ (1:8, 550°C) (**I**), and their corresponding energy-dispersive spectroscopy (EDS) mapping of Ti, Cl, O, and C elements. (**J**) Scanning transmission electron microscopy (STEM) of pristine Ti_3_C_2_Cl*_x_*, showing its atomic structure with the beam along the [$1\bar{1}00$] axis. (**K**) STEM image of reduced Ti_3_C_2_ (1:8, 550°C), showing the atomic structure with the beam along the [$11\bar{2}0$] after removal of -Cl terminations. (**L**) X-ray photoelectron spectroscopy (XPS) results of pristine Ti_3_C_2_Cl*_x_*, annealed Ti_3_C_2_Cl*_x_*, and reduced Ti_3_C_2_ MXenes synthesized under different conditions. (**M**) Raman spectra of pristine Ti_3_C_2_Cl*_x_* and different reduced Ti_3_C_2_ MXenes with a 633-nm laser.

Figure 1A illustrates the synthesis of reduced MXene via the reaction of Ti_3_C_2_Cl*_x_* with metallic Na. The pristine Ti_3_C_2_Cl*_x_* was prepared by etching Ti_3_AlC_2_ MAX phase using a Lewis acidic molten salt (CdCl_2_), as previously reported (*3*, *4*). This precursor was then reacted with Na in a LiCl-KCl molten salt under an inert atmosphere, which can provide a liquid environment at suitable temperatures and thus facilitate reaction kinetics and thermodynamics (fig. S1). The choice of Ti_3_C_2_Cl*_x_* as the starting material and Na as the reductant was guided by two key thermodynamic considerations, as summarized in Fig. 1 (B and C). Figure 1B compares the bond dissociation energies of Ti─O, Ti─F, and Ti─Cl bonds (*29*). The lower bond dissociation energy of Ti─Cl (compared to Ti─O and Ti─F) suggests that -Cl terminations can be more readily removed under moderate thermal conditions, making Ti_3_C_2_Cl*_x_* an ideal precursor for surface reduction. This provides a fundamental basis for selecting Cl-terminated MXenes as the starting material to facilitate efficient termination removal and subsequent electron injection. Figure 1C further evaluates the thermodynamic feasibility of electron injection by comparing the Gibbs free energy of reactions between Ti^3+^ and various reductants. Among these, Na exhibits the lowest Gibbs free energy change, indicating the most favorable electron transfer process. Although LiH and Al also show negative Gibbs free energy, their use often causes structural changes in 2D MXene lattices (*26*, *27*). These strongly support the use of metallic Na as the optimal reductant for maximizing electron injection into the MXene lattice. In the reaction process, Na donates electrons to electronegative -Cl terminations (χ ≈ 3.16 versus Ti ≈ 1.54), converting -Cl termination into Cl^−^ that diffuses into the molten salt (Eq. 1), and Na will be converted into Na^+^ that can combine with Cl^−^ to form NaCl in molten salts. By adjusting Na content and temperature, electron injection was controlled (table S1). With *x* ≈ 2 in Ti_3_C_2_Cl*_x_*, we first tested a 1:2 molar ratio at 500°C to remove -Cl terminations. Increasing Na up to 1:8, we further studied electron injection (Eq. 2). At 500°C, the structure remained intact. Raising the temperature to 550°C with the same ratio further improved electron transfer. Products were labeled as reduced Ti_3_C_2_ (1:2, 500°C), (1:8, 500°C), and (1:8, 550°C), respectively. However, structural degradation occurred at 600°C or a 1:12 ratio (figs. S2 and S3), indicating the accommodation limit of injected electrons. As a result, optimal conditions for maximal electron concentration while preserving the layered structure are 550°C and a 1:8 ratio. This stepwise design not only enables precise control over the reduction degree but also provides insight into the thermodynamic and kinetic factors governing the electron injection mechanism in MXenes.

$${\text{Ti}}_{3}C_{2}{\text{Cl}}_{x}+x\text{Na}={\text{Ti}}_{3}C_{2}+x\text{NaCl}$$ (1)

$${\text{Ti}}_{3}C_{2}+\text{Na}={\left[{\text{Ti}}_{3}C_{2}\right]}^{-}+{\text{Na}}^{+}$$ (2)

X-ray diffraction (XRD) patterns of the pristine Ti_3_C_2_Cl*_x_* and reduced Ti_3_C_2_ synthesized under different conditions are shown in Fig. 1D. XRD, scanning electron microscopy (SEM), and energy-dispersive spectroscopy (EDS) results of Ti_3_C_2_Cl*_x_* annealed in eutectic LiCl-KCl molten salt at 500°C without Na metal show no notable structural and compositional change, eliminating the effect of temperature and molten salt (Fig. 1D and figs. S4 and S5). In contrast, reduced Ti_3_C_2_ samples synthesized under different conditions exhibit notable variations in the (002) peak, corresponding to interlayer spacing values of 1.209 nm [reduced Ti_3_C_2_, (500°C, 1:2)], 1.159 nm [reduced Ti_3_C_2_, (500°C, 1:8)], and 1.252 nm [reduced Ti_3_C_2_, (550°C, 1:8)]. These differences are attributed to the evolution of interlayer interactions during reduction. The removal of -Cl terminations exposes Ti atoms, generating dangling bonds and strong interlayer Coulombic repulsion. This repulsion can lead to increased interlayer spacing, even if retermination occurs during subsequent posttreatment (*30*). As electrons are injected, they occupy Ti-3d orbitals, screening the positive charges and enhancing in-plane metallic bonding, which reduces the interlayer distance (*31*, *32*). However, under excessive electron injection, high-density negative charges accumulate between layers, leading to dominant interlayer Coulombic and Pauli repulsions, resulting in a sudden expansion of the interlayer spacing (*33*). This evolution of interlayer spacing reflects an electronic structure–driven transition in interlayer interactions, offering insight into the tunable structural behavior of MXenes.

Figure 1 (E and F) shows SEM images of pristine Ti_3_C_2_Cl*_x_* and annealed Ti_3_C_2_Cl*_x_*, respectively. Unlike HF-etched MXene, which typically exhibits a distinct accordion-like layered morphology, both particles show compact layered structures due to the strong interlayer binding caused by -Cl terminations (*1*, *3*, *34*). In contrast, the particle of reduced Ti_3_C_2_ (1:8, 550°C) displays a more pronounced accordion-like layered structure, which aligns with the increased interlayer spacing, as observed in XRD results (Fig. 1G and fig. S6). This suggests that the reduction process effectively weakens the interlayer interactions, which is key for the single- or few-layer exfoliation. SEM-EDS point and mapping analyses confirm that the Cl element is almost entirely removed in the reduced samples, providing direct evidence for the elimination of -Cl terminations through surface reduction (figs. S7 to S9). Moreover, the lamellar structure of the Ti_3_C_2_Cl*_x_* and reduced Ti_3_C_2_ (1:8, 550°C) is revealed in the scanning transmission electron microscopy (STEM) images, as shown in Fig. 1 (H to K). STEM-EDS elemental mapping confirms the nearly complete removal of Cl element in the reduced Ti_3_C_2_ sample, indicating highly efficient elimination of -Cl terminations (Fig. 1I). Atomic-resolution STEM images further show structural details at the atomic level. As shown in Fig. 1J, five distinct atomic layers are resolved for each Ti_3_C_2_Cl*_x_* layer, with the brighter atoms corresponding to Ti and the darker ones assigned to Cl. In contrast, the STEM image of the reduced Ti_3_C_2_ sample shows a enlarged interlayer spacing and the absence of Cl atoms, indicating that Na metal effectively reduces and removes the -Cl terminations (Fig. 1K). To further characterize the chemical composition and eliminate the possibility of residual intercalated ions, we performed atomic-scale electron energy-loss spectroscopy (EELS) on reduced Ti_3_C_2_ samples (fig. S10). The atomic-resolution STEM images (fig. S10, A, C, and E) clearly display the well-preserved lamellar atomic structure of MXene after reduction, with no observable disorder or amorphous phases. The corresponding EELS spectra (fig. S10, B, D, and F) only exhibit characteristic peaks of C-*K*, Ti-*L*, and O-*K* edges, with no detectable signals from Na or other foreign ions. This eliminates interlayer intercalation of Na atoms or other ions after reduction and aqueous posttreatment, which may directly interfere with MXene’s intrinsic electronic properties.

The surface chemistry of the MXene samples was further investigated by x-ray photoelectron spectroscopy (XPS) (figs. S11 to S15 and tables S2 to S6). Figure 1L compares the Ti 2p spectra of MXenes synthesized under different conditions. Annealing leads to a stronger TiO_2_ signal compared to pristine Ti_3_C_2_Cl*_x_*, suggesting partial surface oxidation during thermal treatment, likely due to residual oxygen and moisture in the molten salt, even under an inert atmosphere. The Ti─C bonding, originating from the [TiC_6_] octahedral building blocks in the Ti_3_C_2_ structure, appears as three distinct peaks in the Ti 2p spectrum. Notably, these peaks remain at nearly the same binding energy after annealing, suggesting that the thermal treatment itself does not alter the oxidation state of Ti. In contrast, for the reduced Ti_3_C_2_ samples, a clear shift of these Ti─C characteristic peaks toward lower binding energies is observed with increasing reduction degree (Fig. 1L and tables S2 to S6). This downshift indicates electron transfer into the Ti-3d orbitals, effectively reducing the oxidation state of Ti. The extent of the shift provides direct spectroscopic evidence that Na-mediated reduction successfully injects electrons into the MXene lattice and modulates the Ti valence state. However, it should be noted that this peak shift is only suitable for qualitative analysis, as quantitative analysis is hindered by the varying degrees of surface oxidation among the different samples.

Raman spectroscopy (Fig. 1M) further analyzed the surface terminations of the different MXene samples. In the pristine Ti_3_C_2_Cl_x_, the A_1g_ mode at 171 cm^−1^ corresponds to the out-of-plane vibration of surface Cl terminations, confirming the initial Cl-dominated surface (*26*, *35*). The Raman spectra of all reduced MXene samples exhibit the characteristic E_g_ (Ti, C) mode at 363 cm^−1^ and A_1g_ (Ti, C) modes at 213 and 589 cm^−1^, which are intrinsic to the Ti_3_C_2_ lattice structure (*36*–*38*). In the weakly reduced sample [reduced Ti_3_C_2_ (1:2, 500°C)], two additional A_1g_ modes appear at 681 and 748 cm^−1^; these modes arise from the out-of-plane vibrations of sublattice carbon atoms and are highly sensitive to surface termination species, with the 681 cm^−1^ peak assigned to -OH termination and the 748 cm^−1^ peak assigned to -O terminations (*36*–*39*). This indicates a mixed termination state on the MXene surface when the degree of electron injection is low. As the reduction degree increases, however, the 748 cm^−1^ (-O) mode disappears in reduced Ti_3_C_2_ (1:8, 500°C), leaving only the 681 cm^−1^ (-OH) mode. This trend is further reinforced in the reduced Ti_3_C_2_ (1:8, 550°C), where only the 681 cm^−1^ (-OH) mode remains, and its intensity decreases with higher reduction levels. These observations reveal that when MXene undergoes Cl removal without sufficient electron injection, its surface Ti atoms retain the coordination capacity to reterminate with both -OH and -O during aqueous posttreatment. In contrast, increased electron injection fills Ti-3d orbitals, lowering the valence state of Ti and altering its Lewis acid-base properties; i.e., the reduced Ti sites (soft Lewis acid) preferentially coordinate with the soft base (-OH) rather than the hard base (-O), as dictated by the Hard-Soft-Acid-Base (HSAB) principle (*27*, *40*). The impact of valence on surface coordination also explains why Cl^−^ anions cannot reterminate with Ti atoms after removing pristine Cl-terminations in Cl-rich molten salts. Moreover, the electron-enriched Ti sites favor the monodentate coordination of -OH over the bridging bidentate coordination of -O, providing more favorable binding sites for -OH termination (*22*, *23*). These factors collectively result in the surface termination being dominated by -OH in the highly reduced MXene. The domination of -OH termination is further corroborated by Fourier transform infrared (FTIR) spectroscopy (fig. S16), which displays a broad peak around 3500 cm^−1^ corresponding to -OH stretching vibrations (*41*). The intensity of this peak decreases with increasing reduction degree, consistent with the Raman results. Electron paramagnetic resonance (EPR) spectroscopy (fig. S17) was used to further probe the electronic defects in the highly reduced Ti_3_C_2_ (1:8, 550°C) sample. No signal with *g* factor = 1.97 (characteristic of Ti atomic vacancies) was detected, confirming the structural integrity of the MXene lattice (*42*). A weak signal at *g* = 2.003 was observed, which can be assigned to oxygen vacancies in the surface TiO_2_ oxide layer (*43*). This observation aligns with the XPS results showing partial surface oxidation and confirms that our reduction strategy primarily modulates Ti valence via electron injection rather than introducing lattice defects, ensuring the intrinsic lamellar structure of MXenes.

### Direct exfoliation of reduced Ti_3_C_2_ MXene

Given the increase in interlayer spacing observed in previous results, it is evident that the reduction process effectively mimics the effects of preintercalation steps typically required for MXene exfoliation (*34*, *44*). This increased interlayer distance facilitates easier exfoliation of reduced MXene into nanosheets without the need for additional intercalation treatments. Consequently, reduced Ti_3_C_2_ (1:8, 550°C) with the largest interlayer spacing can be directly exfoliated into nanosheets via simple ultrasonication, streamlining the preparation process. However, other reduced Ti_3_C_2_ failed to be exfoliated despite their expanded interlayer spacing and subtle difference with reduced Ti_3_C_2_ (1:8, 550°C) (fig. S18). In contrast, Ti_3_C_2_Cl*_x_* can only be exfoliated after intercalation treatment using tetrabutylammonium hydroxide (TBAOH) (figs. S19 to S21 and table S7). These findings emphasize the significance of interlayer expansion in the exfoliation process and show the advantage of our reduction strategy.

**Fig. 2.**
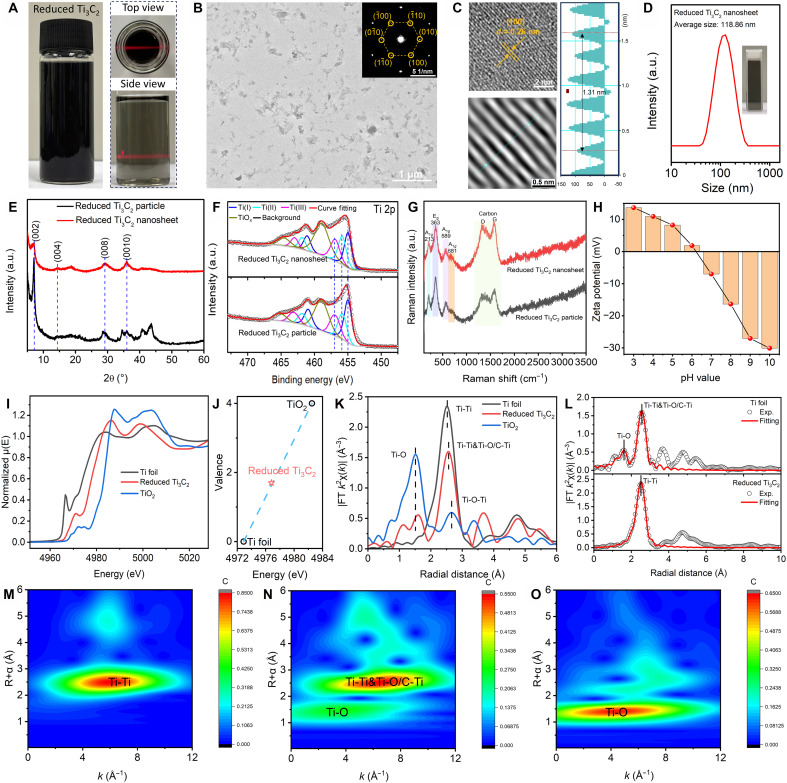
Preparation of reduced Ti_3_C_2_ (1:8, 550°C) nanosheets. (**A**) Optical photograph of the reduced Ti_3_C_2_ nanosheet suspension with a high concentration (left) and a diluted concentration (right), showing the Tyndall effect under laser illumination. (**B**) TEM image of the reduced Ti_3_C_2_ (1:8, 550°C) nanosheets, and the inset shows the selected-area electron diffraction (SAED) pattern corresponding to the hexagonal structure of Ti_3_C_2_, indicating preserved crystallinity. (**C**) High-resolution TEM lattice image indexed to the (100) plane of reduced Ti_3_C_2_ (1:8, 550°C) and its corresponding Fourier transform of the selected area (left), and the measured lattice spacing based on the Fourier transform. (**D**) Dynamic light scattering (DLS) size distribution of the as-obtained reduced Ti_3_C_2_ (1:8, 550°C) nanosheets. (**E**) XRD patterns of both reduced Ti_3_C_2_ MXene particles and their corresponding exfoliated nanosheets. (**F**) XPS spectra of reduced Ti_3_C_2_ particles and nanosheets. (**G**) Raman spectra of reduced Ti_3_C_2_ (1:8, 550°C) particles and nanosheets. (**H**) Zeta potentials of reduced Ti_3_C_2_ (1:8, 550°C) nanosheets with the change of pH values. (**I**) Ti *K*-edge x-ray absorption near edge structure (XANES) spectra of Ti foil, reduced Ti_3_C_2_ (1:8, 550°C) nanosheets, and TiO_2_. (**J**) Valence state plot of Ti species, with Ti foil, reduced Ti_3_C_2_ (1:8, 550°C) nanosheets, and TiO_2_ positioned to show the variation in Ti valence. (**K**) Fourier transform extended x-ray absorption fine structure (FT-EXAFS) spectra of Ti foil, reduced Ti_3_C_2_ (1:8, 550°C) nanosheets, and TiO_2_ in *R*-space. (**L**) FT-EXAFS and fitting curves for Ti foil in *R*-space. The wavelet transform analysis in Ti *K*-edge for Ti foil (**M**), reduced Ti_3_C_2_ (1:8, 550°C) nanosheets (**N**), and TiO_2_ (**O**).

The optical photograph in Fig. 2A demonstrates the successful exfoliation of reduced Ti_3_C_2_ (1:8, 550°C) into nanosheets, as evidenced by the pronounced Tyndall effect observed under laser illumination. This indicates a stable colloidal suspension, which is crucial for subsequent applications. TEM image (Fig. 2B) reveals that the reduced Ti_3_C_2_ (1:8, 550°C) nanosheets exhibit a highly uniform morphology with consistent particle sizes. The homogeneous distribution of these nanosheets suggests efficient delamination, leading to high-quality MXene nanosheets suitable for various applications. The selected-area electron diffraction (SAED) pattern shown in the inset of Fig. 2B confirms the hexagonal crystal structure of Ti_3_C_2_, indicating that the reduction process did not alter the fundamental crystal structure. This is essential for maintaining the intrinsic electronic and optical properties of the MXene. High-resolution TEM image (Fig. 2C) provides further insight into the atomic structure of the reduced Ti_3_C_2_ (1:8, 550°C) nanosheets. The lattice fringes are indexed to the (100) plane, confirming the structural integrity of the reduced MXene nanosheets. Figure 2D presents the particle size distribution of the exfoliated nanosheets, with an average size of 118.86 nm, highlighting their uniformity and narrow size range. This uniformity is critical for ensuring consistent performance in practical applications. XRD analysis (Fig. 2E) compares the diffraction patterns of reduced Ti_3_C_2_ (1:8, 550°C) particles and their exfoliated nanosheets. Both samples exhibit characteristic peaks corresponding to the hexagonal phase of Ti_3_C_2_ MXenes. However, the exfoliated nanosheets show only (00*l*) plane reflections, indicating a preferred orientation. XPS (Fig. 2F and fig. S22) was used to compare the chemical states of Ti_3_C_2_ in both its nanoparticle and nanosheet forms. The XPS spectra of reduced Ti_3_C_2_ (1:8, 550°C) produced freshly and after 24 hours confirm that the Ti oxidation state remains unchanged between the two forms, indicating that the exfoliation process does not alter the chemical state of Ti (figs. S22 and S23 and tables S8 and S9). Specifically, the presence of Ti─C bonds and the absence of Cl signals validate the complete removal of -Cl terminations in both samples (Fig. 2F and fig. S22). The consistency in binding energies across both forms underscores the structural and chemical stability of the reduced Ti_3_C_2_ (1:8, 550°C) during sonication. Notably, the sonication process must be operated in ascorbic acid solution to avoid the oxidation caused by the high-energy damage and dissolved oxygen (fig. S24 and table S10). Moreover, a filtration and redispersion process to remove ascorbic acid will not cause obvious agglomeration (fig. S25).

Raman spectroscopy (Fig. 2G) further confirms the structural consistency and surface termination characteristics of reduced Ti_3_C_2_ (1:8, 550°C) particles and nanosheets. Both samples exhibit the intrinsic lattice modes of Ti_3_C_2_, including the E_g_ (Ti, C) mode at 363 cm^−1^ and A_1g_ (Ti, C) modes at 213 and 589 cm^−1^, verifying that ultrasonic exfoliation does not disrupt the basal plane structure of MXene. Meanwhile, the presence of the 681 cm^−1^ A_1g_ mode (assigned to -OH termination) confirms that the surface of both particles and nanosheets maintains the OH-dominated termination state. This consistency in surface chemistry highlights that exfoliation does not alter the selective retermination behavior of reduced MXene. Zeta potential measurements (Fig. 2H) of the exfoliated reduced Ti_3_C_2_ (1:8, 550°C) nanosheets as a function of pH reveal an isoelectric point (IEP) near neutral, which aligns with our previous discussion on OH-dominated surface terminations. The zeta potential undergoes a gradual transition from positive to negative with the increase in pH, reflecting the protonation/deprotonation equilibrium of surface -OH groups (Ti-OH_2_^+^ ⇌ Ti-OH ⇌ Ti-O^−^), which is consistent with the OH-terminated TiO_2_ (*45*). In contrast, Ti_3_C_2_T*_x_* nanosheets prepared via the traditional HF method exhibit a distinct acidic IEP value (fig. S26), further confirming the unique OH-dominated surface chemistry of our reduced MXene. FTIR spectroscopy (fig. S27) provides additional evidence for the absence of ascorbic acid residues in the exfoliated nanosheets, as no characteristic C═O absorption band is detected. The broad peak around 3431 cm^−1^ corresponds to -OH stretching vibrations, consistent with Raman results and further validating the OH-dominated surface. TEM and EELS characterization (fig. S28) of the exfoliated nanosheets confirms their lamellar morphology and chemical purity, with EELS spectra only showing characteristic peaks of C-*K*, Ti-*L*, and O-*K* edges, verifying that neither the reduction nor exfoliation process introduces residual impurities. EPR spectroscopy (fig. S29) further supports structural integrity, with no Ti vacancy signal detected; only a weak signal at *g* = 2.003 assigned to O vacancies in minor surface TiO_2_ is observed, consistent with earlier XPS results of slight surface oxidation, but not affecting the overall lattice stability (*42*). Photographs of reduced Ti_3_C_2_ (1:8, 550°C) nanosheet suspensions after one to five ultrasonic exfoliation cycles (using 0.5 g of starting reduced Ti_3_C_2_ powder) further confirm the efficiency and reproducibility of the exfoliation process (fig. S30). Quantitative analysis of the exfoliated product reveals an overall concentration of 268 μg/ml and an overall yield of ~27% from the starting powder. Dynamic light scattering (DLS) analysis of nanosheets after five ultrasonic exfoliation cycles (fig. S31) shows an average hydrodynamic diameter of 122.9 nm, which is consistent with the initial 118.86 nm (Fig. 2D). This minor size variation is attributed to minimal internanosheet interactions during repeated sonication, yet it does not compromise the overall uniformity critical for consistent application performance.

To gain insight into the electronic and structural evolution of reduced Ti_3_C_2_ (1:8, 550°C) nanosheets induced by the reduction process, Ti *K*-edge x-ray absorption fine structure (XAFS) measurements of reduced Ti_3_C_2_ nanosheets (1:8, 550°C) were performed, with Ti foil (metallic Ti^0^) and TiO_2_ (Ti^4+^) serving as reference materials (Fig. 2, I to O). The Ti *K*-edge x-ray absorption near-edge structure (XANES) spectra (Fig. 2I) directly reflect the variation in Ti valence states. The absorption edge of reduced Ti_3_C_2_ (1:8, 550°C) nanosheets lies between the two references, an indication that its Ti valence is intermediate between 0 and +4, resulting from the reduction treatment. This valence trend is further quantified by the valence state plot (Fig. 2J). The Ti valence of reduced Ti_3_C_2_ nanosheets is approximately 1.6, which marks a substantial reduction compared to the ~3.0 valence of pristine Ti_3_C_2_ (*46*). Such a valence drop confirms that the reduction process effectively reduces Ti species in the MXene, leading to a high degree of Ti valence lowering that is consistent with the XPS results.

The local atomic environment of Ti was consistently illustrated by both the *k*-space extended x-ray absorption fine structure (EXAFS) oscillation spectra (figs. S32 and S33) and Fourier transform (FT) EXAFS spectra in *R*-space (Fig. 2, K and L, and fig. S34). Figure 2K shows FT-EXAFS spectra of Ti foil, reduced Ti_3_C_2_ nanosheets, and TiO_2_ in *R*-space. Ti foil exhibits a single sharp peak corresponding to Ti─Ti coordination, while TiO_2_ shows Ti─O characteristic peaks. Reduced Ti_3_C_2_ nanosheets display multiple peaks, corresponding to Ti─O and Ti─O/C─Ti coordinations, confirming its mixed local atomic environment. The Ti─O/C─Ti peak exhibits a slight angular offset relative to the Ti foil, which is attributed to the surface coordination. Figure 2L presents the FT-EXAFS spectrum and fitting curve of Ti foil and reduced Ti_3_C_2_ in *R*-space, which closely overlaps with the experimental data (table S11). To visualize the spatial distribution of these coordination features, *k*-*R* space contour plots (Fig. 2, M to O) were constructed. Ti foil (Fig. 2M) shows only Ti─Ti correlation, TiO_2_ (Fig. 2O) shows only Ti─O correlation, and reduced Ti_3_C_2_ nanosheets (Fig. 2N) clearly display both Ti─O/C─Ti and Ti─O correlations.

### Electronic behavior analysis

**Fig. 3.**
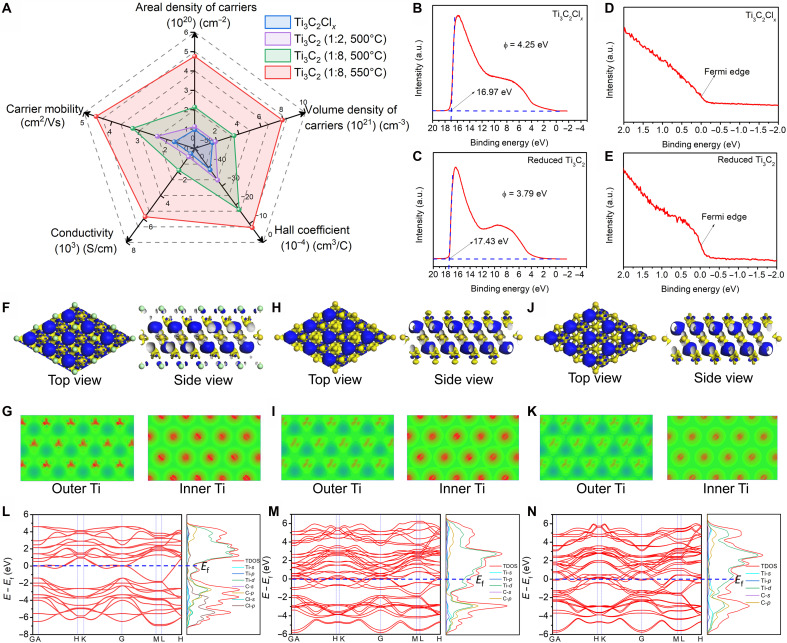
Electronic structure analysis. (**A**) Radar map of the electronic structure information of different MXenes based on the test of the Hall effect measurement, showing the electron concentration, mobility, conductivity, and Hall coefficient. Ultraviolet photoelectronic spectra of Ti_3_C_2_Cl*_x_* nanosheets (**B**) and reduced Ti_3_C_2_ (1:8, 550°C) nanosheets (**C**) and their corresponding Fermi edges (**D** and **E**), respectively. Three-dimensional (3D) differential charge density mappings of Ti_3_C_2_Cl*_x_* (**F**) and its corresponding in-plane 2D charge density images (**G**). 3D differential charge density mappings of Ti_3_C_2_ without terminations (**H**) and its corresponding in-plane 2D charge density images (**I**). 3D differential charge density mappings of Ti_3_C_2_ injected with electrons (**J**) and its corresponding in-plane 2D charge density images (**K**). The yellow and blue isosurfaces correspond to electron deletion and accumulation, respectively. In-plane 2D charge density images show the charge distribution of outer and inner Ti, where red represents the electron deletion, and green represents the neutral background. Energy band structures and density of state (DOS) of Ti_3_C_2_Cl*_x_* (**L**), reduced Ti_3_C_2_ without terminations (**M**), and reduced Ti_3_C_2_ injected with electrons (**N**).

The electronic properties of pristine Ti_3_C_2_Cl*_x_* and reduced Ti_3_C_2_ particles prepared under different conditions were systematically investigated via Hall effect measurements (Fig. 3A, fig. S35, and table S12), with key parameters including free electron areal/volume density, carrier mobility, conductivity, and Hall coefficient comprehensively characterized to clarify the intrinsic correlation between surface termination evolution, electron injection, and MXene’s electronic behavior.

Figure 3A intuitively presents the variation of electronic properties across samples. Compared to pristine Ti_3_C_2_Cl*_x_*, the annealed Ti_3_C_2_Cl*_x_* (500°C) exhibits nearly identical electron concentration and mobility (fig. S36 and table S12), excluding the interference of thermal effect and molten salts on subsequent reduction results. For the weakly reduced Ti_3_C_2_ (1:2, 500°C), the free electron volume density only slightly increases (from 1.61 × 10^21^ to 1.88 × 10^21^ cm^−3^), while the carrier mobility rises by ~35.5% (from 1.74 to 2.36 cm^2^/V·s) (Fig. 3A and table S12). This phenomenon is attributed to the selective retermination behavior after Cl removal (Fig. 1M), whose weak electron-withdrawing and electron-scattering effects lead to the slightly increased electron concentration and increased carrier mobility (*47*). To further reveal the mechanism of electron injection regulating electronic properties, we further studied the electronic behavior of different reduced MXenes (table S12). As a typical MXene, Ti_3_C_2_ features a unique hybrid orbital structure, where Ti’s low-energy t_2g_ orbitals hybridize with C-p orbitals to form a delocalized π-bond network, while the high-energy e_g_ orbitals hybridize with C-p orbitals to construct σ bonds that stabilize the lamellar structure. The adjacent Ti atoms form metal bonds with t_2g_ orbitals (*48*, *49*). Therefore, the Ti-3d orbitals dominate the electronic properties of Ti_3_C_2_. Electrons donated by Na first fill the unoccupied low-energy t_2g_ states. At this stage, the injected electrons remain localized in these states and do not contribute to delocalized conduction, so the free electron concentration does not increase notably. This is verified by the reduced Ti_3_C_2_ (1:4, 500°C) sample (fig. S37), where Ti *K*-edge XANES spectra and valence state analysis (figs. S38 to S40) show +2.1 Ti valence, confirming effective electron injection-induced valence reduction. However, Hall effect results (fig. S41 and table S12) reveal that its electron volume density is nearly identical to that of reduced Ti_3_C_2_ (1:2, 500°C), which directly proves that electron filling of low-energy localized states does not contribute to free electron concentration.

Upon saturation of localized Ti-3d states with increasing Na ratio and temperature, additional electrons are injected into delocalized states near the Fermi level, resulting in a marked increase in free electron concentration and further reduction of the Ti valence state. As shown in Fig. 3A and table S12, reduced Ti_3_C_2_ (1:8, 500°C) exhibits an electron volume density of 3.56 × 10^21^ cm^−3^ (2.21 times that of pristine Ti_3_C_2_Cl*_x_*) and a mobility of 3.25 cm^2^/V·s (1.87 times), resulting in a conductivity of 1853.03 S/cm (4.13 times); the reduced Ti_3_C_2_ (1:8, 550°C) sample, with stronger reduction, achieves an electron volume density of 7.93 × 10^21^ cm^−3^ (4.92 times), a mobility of 4.57 cm^2^/V·s (2.63 times), and a conductivity of 5811.42 S/cm (12.96 times), attributed to the saturated filling of Ti-3d states and massive electron delocalization. Notably, excessive reduction [e.g., reduced Ti_3_C_2_ (1:10, 550°C)] triggers a structural transition from Ti_3_C_2_ MXene to TiC (figs. S42 and S43). Consequently, its electron volume density drops to 2.78 × 10^21^ cm^−3^, and conductivity decreases to 1366.22 S/cm, indicating that moderate electron injection is the key to optimizing MXene’s electronic properties.

In addition, ultraviolet photoelectron spectroscopy (UPS) was conducted on both the pristine Ti_3_C_2_Cl*_x_* and the most effectively reduced Ti_3_C_2_ (1:8, 550°C) nanosheets. As shown in Fig. 3 (B and C), the work function of the reduced MXene decreases from 4.25 to 3.79 eV, which is primarily attributed to the large amount of electron injection during the reduction process. The comparison of Fermi edge features between the two samples reveals a much steeper slope in the reduced Ti_3_C_2_, indicating a higher concentration of free electrons near the Fermi level (Fig. 3, D and E). This sharp increase in available free electrons results in a stronger photoelectron signal upon ultraviolet excitation, directly supporting the conclusion that the reduction process greatly enhances the DOS near the Fermi level.

To gain deeper insight into how reduction affects the electronic structure of MXene, theoretical calculations were performed based on an ideal termination-free Ti_3_C_2_ model (figs. S44 to S47 and table S13). This simplification was adopted to decouple and separately elucidate the effects of two key reduction-related processes, surface coordination optimization and electron injection, on electronic structure, despite the retermination in the actual experimental material. As illustrated in Fig. 3 (F and G) and fig. S44, the -Cl terminations in pristine Ti_3_C_2_Cl*_x_*, due to their high electronegativity, effectively withdraw electrons from the outer Ti atoms, resulting in lower electron concentration in these regions. Inner Ti atoms, however, remain largely unaffected by the presence of -Cl terminations. Upon removal of the terminations, the suppression of electron concentration at the outer layers is lifted, leading to a noticeable increase in electron availability (Fig. 3, H and I, and fig. S45). When additional electrons are injected into the system, both outer and inner Ti atoms experience a notable enhancement in electron concentration, demonstrating that the reduction not only affects the surface but also penetrates the inner structure (Fig. 3, J and K, and fig. S46). Further analysis of the energy band structure and DOS reveals that in pristine Ti_3_C_2_Cl*_x_*, the presence of -Cl terminations causes the electrons near the Fermi level to be more dispersed (Fig. 3L). After termination removal, these electrons begin to accumulate closer to the Fermi level, increasing the local charge density and improving conductivity (Fig. 3M). With continued electron injection, the electron density at the Fermi level increases even further, leading to a marked enhancement in electrical properties (Fig. 3N). However, excessive electron injection will cause the structural transformation, which is consistent with the experimental results (fig. S47). These theoretical observations align closely with the experimental data, providing a unified explanation for the observed improvements in free electron concentration, mobility, and overall conductivity in reduced MXene materials.

### Photothermal property study

On the basis of our previous findings, the surface reduction process enhances the free electron concentration in MXenes. This increase in free electron concentration is expected to have a profound impact on their optical and electronic properties, particularly those related to photothermal conversion. Thus, we investigated how the surface reduction strategy affects the photothermal behavior of MXenes.

**Fig. 4.**
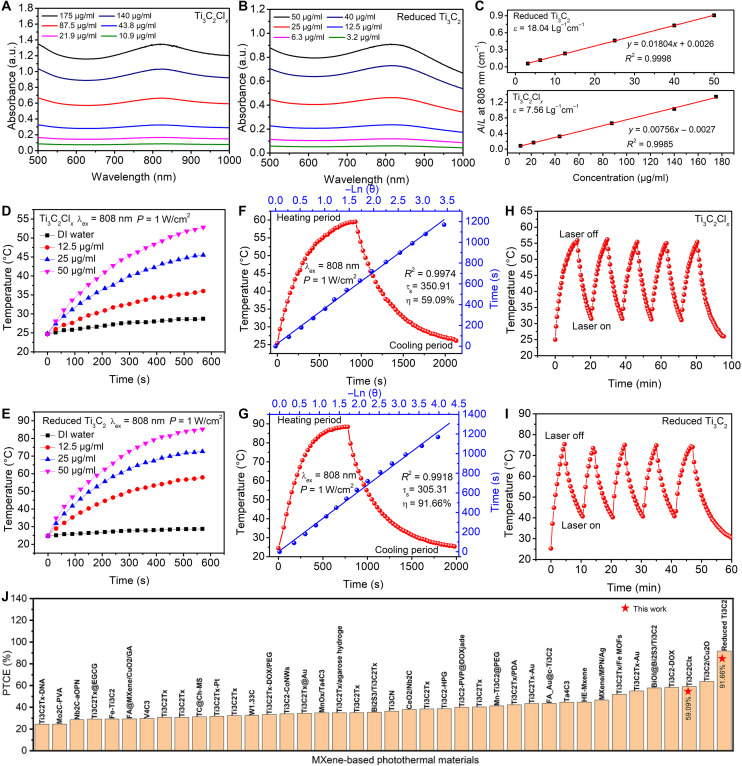
Photothermal-conversion performance of pristine Ti_3_C_2_Cl*_x_* and reduced Ti_3_C_2_ (550°C, 1:8) nanosheets. (**A**) Ultraviolet-visible spectroscopy (UV-Vis) absorbance spectra of aqueous suspensions of dispersed Ti_3_C_2_Cl*_x_* nanosheets at varied concentrations (10.9, 21.9, 43.8, 87.5, 140, and 175 μg ml^−1^) (**B**) UV-Vis absorbance spectra of aqueous suspensions of dispersed reduced Ti_3_C_2_ (1:8, 550°C) nanosheets at varied concentrations (3.2, 6.3, 12.5, 25, 40, and 50 μg ml^−1^). (**C**) Mass extinction coefficient of Ti_3_C_2_Cl*_x_* and reduced Ti_3_C_2_ (1:8, 550°C) nanosheets at 808 nm. Normalized absorbance intensity at λ = 808 nm divided by the characteristic length of the cell (*A*/*L*) at varied concentrations. Photothermal heating curves of aqueous suspensions of dispersed Ti_3_C_2_Cl*_x_* (**D**) and reduced Ti_3_C_2_ (1:8, 550°C) (**E**) nanosheets under irradiation of an 808-nm laser at varied concentrations (12.5, 25, and 50 μg ml^−1^). Calculation of PTCE at 808 nm for Ti_3_C_2_Cl*_x_* (**F**) and reduced Ti_3_C_2_ (1:8, 550°C) (**G**) nanosheets. Red line: Temperature rise during laser irradiation and temperature decrease after turning off the laser; blue line: cooling phase with fitted time constant (τ_s_) for heat dissipation. Stability test over five on/off laser cycles (1 W/cm^2^) for Ti_3_C_2_Cl*_x_* (**H**) and reduced Ti_3_C_2_ (1:8, 550°C) (**I**) nanosheets. (**J**) Summary of the PTEC of different MXene-based nanosystems, references from table S14.

The surface reduction process enhances the free electron concentration in MXenes, which is expected to profoundly optimize their photothermal conversion performance by regulating the LSPR mechanism. The photothermal conversion performance of pristine Ti_3_C_2_Cl*_x_* and reduced Ti_3_C_2_ (550°C, 1:8) nanosheets was evaluated using ultraviolet-visible (UV-Vis) spectroscopy and laser irradiation experiments. As shown in Fig. 4 (A and B), both materials exhibit absorption in the NIR region, which is essential for efficient photothermal conversion. However, a notable blue shift in the absorption peak is observed for the reduced Ti_3_C_2_ compared to the pristine Ti_3_C_2_Cl*_x_* (fig. S48). This blue shift suggests a change in the LSPR wavelength, which may be attributed to the increased free electron concentration and surface coordination optimization. Figure 4C compares the mass extinction coefficient at 808 nm for both samples. The reduced Ti_3_C_2_ exhibits a much higher extinction coefficient (18.04 L g^−1^ cm^−1^) than the pristine Ti_3_C_2_Cl*_x_* (7.56 L g^−1^ cm^−1^). This confirms the enhanced light absorption capability after reduction, which originates from the increased electron concentration.

Photothermal heating curves under 808-nm laser irradiation (Fig. 4, D and E) show that the reduced Ti_3_C_2_ achieves a higher temperature rise at the same power density and concentration. This superior photothermal performance can be attributed to both the enhanced light absorption due to increased free electron concentration and the improved charge transport properties resulting from the removal of surface scattering centers. PTCE was calculated based on the temperature rise and cooling rate (Fig. 4, F and G, and fig. S49). The reduced Ti_3_C_2_ shows a high efficiency of 91.66%, indicating an enhancement of approximately 55.12% compared to 59.09% for the pristine Ti_3_C_2_Cl*_x_*. This superior photothermal performance is essentially governed by the LSPR mechanism, strongly enhanced by the electronic regulation effect of our reduction strategy (*13*, *50*). Upon 808-nm laser illumination, the increased free electron concentration in reduced Ti_3_C_2_ enables intense coherent oscillation of electrons when photon energy matches the LSPR absorption band, further amplifying light absorption (*51*, *52*). The excited plasmons undergo nonradiative decay via Landau damping, transferring energy to electrons and then to the lattice through electron-electron and electron-phonon scattering, ultimately achieving efficient light-to-heat conversion (*13*, *50*). Meanwhile, the surface coordination optimization reduces electron scattering, further promoting charge transport and energy transfer efficiency. This mechanism is well supported by the enhanced extinction coefficient (Fig. 4C), while the remarkable temperature rise and PTCE confirm efficient energy conversion. Moreover, stability tests over five laser on/off cycles (Fig. 4, H and I) demonstrate that both the pristine Ti_3_C_2_Cl*_x_* and reduced Ti_3_C_2_ samples exhibit good photothermal stability, maintaining consistent heating and cooling profiles without notable degradation. To further verify the long-term photothermal durability of the reduced Ti_3_C_2_ nanosheets, an extended stability test involving 15 on/off laser cycles was conducted on a reduced Ti_3_C_2_ nanosheet solution (50 μg/ml; fig. S50). It is worth noting that, to the best of our knowledge, this obtained PTCE of reduced Ti_3_C_2_ is the highest reported value for MXene materials at 808-nm irradiation (Fig. 4J and table S14) (*12*, *20*, *53*–*79*).

### Application exploration on the photothermal antibacterial woundplast

Chronic wounds are particularly susceptible to bacterial infection due to impaired immune responses, poor vascularization, and delayed healing (*80*). Traditional treatments, such as systemic antibiotics, topical agents, and debridement, often face drug resistance, cytotoxicity, incomplete efficacy, and lack of localized control, highlighting the need for more effective and targeted therapeutic strategies (*81*). In this context, photothermal MXenes offer promising potential for localized, noninvasive, and efficient infection control. To explore the biomedical potential of reduced Ti_3_C_2_ MXene for chronic wound applications, its cytotoxicity and biocompatibility were first evaluated using human umbilical vein endothelial cells and Raw 264.7 cells through standard cell toxicity assays and live/dead cell viability staining (figs. S51 to S53). The results show negligible cytotoxicity across all tested concentrations, with more than 90% cell viability maintained even at a high MXene loading, demonstrating excellent biocompatibility and laying the foundation for further biomedical applications.

**Fig. 5.**
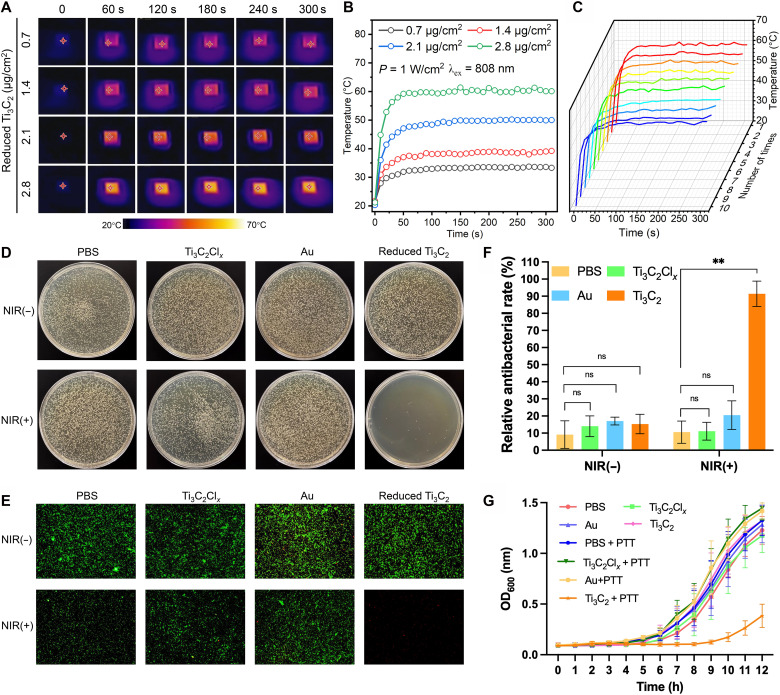
The photothermal antibacterial effect of woundplast loaded with reduced Ti_3_C_2_ nanosheets (1:8, 550°C). (**A**) Infrared thermal images of woundplast loaded with reduced Ti_3_C_2_ at different concentrations under 808 nm laser irradiation with a power density of 1 W/cm^2^. (**B**) Photothermal heating curves of woundplast loaded with reduced Ti_3_C_2_ at different concentrations under 808-nm laser irradiation with a power density of 1 W/cm^2^. (**C**) Photothermal cycling stability of woundplast loaded with reduced Ti_3_C_2_ at different concentrations under 808-nm laser irradiation with a power density of 1 W/cm^2^. (**D**) Colony-forming unit (CFU) assay results of antibacterial treatments using woundplast loaded with different photothermal agents. (**E**) Fluorescence images of bacterial live/dead staining showing the survival and death of bacteria after treatment with woundplast loaded with different photothermal agents. Live bacteria are stained green, while dead bacteria are stained red. (**F**) Relative antibacterial rates of different treatment groups. (**G**) Bacterial growth curves over 12 hours. Data are presented as mean ± SD. Compared to the control group, statistically significant differences were observed: *P < 0.05, **P < 0.01, ***P < 0.001; ns, not significant.

On the basis of these findings, a photothermal antibacterial woundplast was fabricated using reduced Ti_3_C_2_ MXene (1:8, 550°C) (figs. S54 and S55). Considering the safe temperature range for photothermal therapy (42° to 60°C), samples were irradiated with an 808-nm laser (1.0 W/cm^2^), and real-time temperatures were recorded using infrared imaging (Fig. 5, A and B). The 2.8-μg/cm^2^ group reaches ~60°C within 1 min, achieving effective photothermal performance while minimizing thermal damage risk. In addition, this sample exhibits excellent cyclic thermal stability; i.e., it maintains consistent performance even after 10 on/off cycles (Fig. 5C), and under continuous heating at ~60°C for up to 90 min (fig. S56), with no obvious degradation in its thermal response observed. Thus, 2.8 μg/cm^2^ was selected as the optimal loading concentration. Four control groups, AuNPs, phosphate-buffered saline (PBS), pristine Ti_3_C_2_Cl*_x_*, and reduced Ti_3_C_2_, were prepared and tested under identical conditions (figs. S57 to S60). Only the reduced Ti_3_C_2_ group exhibits rapid heating; no notable temperature rise is observed in AuNPs or Ti_3_C_2_Cl*_x_* groups (see movies S1 and S2).

For antimicrobial evaluation, *S. aureus* was used as the model bacterial strain. As shown in Fig. 5D, colony-forming unit (CFU) assays demonstrate that woundplast loaded with reduced Ti_3_C_2_ exhibits excellent antibacterial performance under 808-nm laser irradiation, with nearly no bacterial colonies observed. In contrast, minimal antibacterial effects are seen in the PBS, AuNPs, and Ti_3_C_2_Cl*_x_* groups, highlighting the essential role of the photothermal effect. Figure 5E presents the live/dead staining results, where green fluorescence indicates live bacteria and red fluorescence indicates dead bacteria, providing a direct visual assessment of bacterial viability after treatment. Quantitative analysis in Fig. 5F shows that the reduced Ti_3_C_2_ group achieved an antibacterial rate of 91.39% under NIR irradiation, substantially higher than that of the Ti_3_C_2_Cl*_x_* or AuNPs group, which is attributed to the suitable temperature (fig. S61). No notable antibacterial effect is observed without laser irradiation, further confirming that the photothermal effect is the main mechanism of bacterial inactivation. To evaluate long-term antibacterial performance, bacterial growth was monitored over 12 hours using optical density at 600 nm (OD_600_) measurements (Fig. 5G). The reduced Ti_3_C_2_ group under laser irradiation maintains consistently low optical density values, indicating effective and sustained inhibition of bacterial proliferation, whereas all other groups showed rapid regrowth within a few hours. These results highlight the superior and durable antimicrobial capability of MXene-based photothermal treatment.

Notably, while the in vitro results demonstrate strong antibacterial efficacy, it is important to consider the influence of baseline body temperature (~37°C) in vivo, which may affect the local thermal response during photothermal therapy. Therefore, precise regulation of MXene loading will be critical in future in vivo studies to ensure both therapeutic effectiveness and thermal safety when applied to living tissues. Given the high susceptibility of chronic wounds to infection and the limitations of current antibiotic therapies, this MXene-based photothermal woundplast offers a promising alternative, enabling rapid, localized, and durable bacterial elimination without systemic side effects. Future work will focus on evaluating its performance in chronic wound models to assess its translational potential in clinical settings.

## DISCUSSION

In summary, we developed a Na-mediated surface reduction strategy for MXenes in molten salts, whose core advantage lies in realizing electronic regulation of MXene through a well-defined two-step electron injection mechanism, distinguishing it from conventional surface modification or doping approaches. Specifically, this strategy achieves dual effects, i.e., effectively reducing electron-withdrawing and electron-scattering effects by optimizing the surface coordination, and enabling direct electron injection into the Ti-3d states of Ti_3_C_2_ MXene. Systematic characterizations clarify the underlying regulation mechanism: (i) In the weak reduction stage (low Na ratio/temperature), the elimination of -Cl terminations and retermination process optimizes surface coordination, primarily enhancing carrier mobility by reducing electron scattering and slightly increasing electron concentration; (ii) with moderate-to-strong reduction (optimal Na ratio/temperature), continuous electron injection first fills the localized Ti-3d states and then drives electron delocalization upon localized state saturation. This dual process boosts free electron concentration and conductivity. This mechanism clarifies the intrinsic correlation between Ti-3d state filling, electron localization/delocalization, and functional behaviors of MXenes. Through this precise electronic regulation, the optimized reduced Ti_3_C_2_ MXene achieves a remarkable PTCE of 91.66% under 808-nm laser irradiation, with a bacterial kill rate of 91.39% in a model woundplast application.

Notably, this Na-mediated electron injection method exhibits remarkable universality and multifield extensibility. Unlike methods restricted by specific surface terminations or elemental compositions, it targets the intrinsic electronic structure of MXene, making it applicable to the entire MXene family (e.g., Ti_2_CT*_x_*, Nb_2_CT*_x_*, and V_4_C_3_T*_x_*) containing transition metal atoms. This system serves as an electron reservoir, supplying sufficient electrons to fill M-d orbitals, reduce M valence states, and optimize electronic structures, ensuring reliable and tunable reduction effects.

Furthermore, the electron-enriched MXene enabled by this strategy opens up cross-field opportunities. In biomedicine, the electron-enriched Ti sites with low valence display strong affinity for reactive oxygen species (ROS) and efficient electron transfer for ROS scavenging, mimicking natural antioxidant enzymes to address key challenges in anti-inflammatory, antioxidative, and tissue repair applications. In energy storage and conversion, the enhanced electron donation capability of reduced Ti sites facilitates stable single-atom loading (e.g., Pt, Fe, and Co) and efficient heterojunction construction, promoting interfacial charge transfer for electrocatalysis. For intercalation compound engineering, the synergy between MXene’s interlayer channels and the strong reducing ability of electron-enriched Ti sites enables efficient coupling with guest species (metal ions and organic intercalants), supporting the development of high-performance batteries and supercapacitors.

Collectively, this work highlights the critical role of surface chemistry and electron concentration in regulating MXene properties and establishes the Na-mediated electron injection strategy as a versatile platform for atomic-level electronic regulation of MXenes. The reduced MXene not only excels in photothermal conversion and antibacterial applications but also holds great potential in broader fields requiring tailored surface chemistry and high electron concentration. Despite these advantages, there are still some challenges, such as large-scale production and more advanced in situ characterization for the chemistry mechanism, which is essential for future development.

## MATERIALS AND METHODS

### Materials

The following materials were used in the study: Anhydrous cadmium chloride (CdCl_2_, Aladdin Scientific, ≥99%), potassium chloride (KCl, Aladdin Scientific, ≥99.5%), lithium chloride (LiCl, aladdin, ≥99.5%), sodium chloride (NaCl, Aladdin Scientific, ≥99.5%), ascorbic acid (Aladdin Scientific, ≥99%), hydrochloric acid (HCl, Anaqua, 37%), metallic sodium (Na) (Sigma-Aldrich, ≥99.9%), gold (Au) nanoparticle solution (Tomicro Technology Co. Ltd., 50 μg ml^−1^), Ti_3_AlC_2_ (Laizhou Kai Kai ceramic Materials Co. Ltd., ≥98%), Ti_3_C_2_T*_x_* suspension solution (Laizhou Kai Kai ceramic Materials Co. Ltd., 1 mg/ml), Dulbecco’s modified Eagle’s medium (Thermo Fisher Scientific), fetal bovine serum (Thermo Fisher Scientific), penicillin-streptomycin (Thermo Fisher Scientific), Cell Counting Kit-8 (Beyotime), calcein AM/propidium iodide staining kit (Beyotime), PBS (Thermo Fisher Scientific), Luria-Bertani (LB) broth (Qingdao Hope Bio-Technology Co. Ltd.), and commercial woundplast (Henan Maidinkang Medical Technology Co. Ltd.)

### Synthesis of Ti_3_C_2_Cl*_x_*

Ti_3_C_2_Cl*_x_* was synthesized using a nitrogen atmosphere tube furnace. In a typical procedure, the starting MAX phase (Ti_3_AlC_2_) and the etching agent (CdCl_2_) were weighed in a molar ratio of 1:3 and thoroughly mixed in an agate mortar to ensure homogeneity. The resulting powder mixture was transferred into an alumina crucible and placed inside the tube furnace under a continuous flow of nitrogen gas. The furnace was heated to 700°C at a rate of 5°C/min and held at this temperature for 5 hours to allow for complete reaction. After the reaction, the system was naturally cooled to room temperature under nitrogen protection. The resulting black product was collected for further purification. To remove residual molten salts and metallic by-products, the reacted powder was first washed repeatedly with deionized water. Subsequently, to effectively eliminate any remaining Cd metal, the sample was treated with a 1 M HCl solution under vigorous stirring. This step ensured the selective dissolution of metallic Cd formed during the reaction. Following this washing, the mixture was separated via vacuum filtration and rinsed thoroughly with deionized water until the filtrate reached a neutral pH. Last, the purified product was dried under vacuum at 60°C overnight and stored in an inert atmosphere for subsequent characterization and analysis.

### Synthesis of reduced Ti_3_C_2_

Reduced Ti_3_C_2_ samples were prepared by thermally treating as-synthesized Ti_3_C_2_Cl*_x_* in the presence of sodium (Na) metal as a reducing agent. A specific amount of Ti_3_C_2_Cl*_x_* and Na was weighed according to the desired molar ratios and mixed thoroughly with LiCl-KCl molten salt in an agate mortar. The detailed recipes and conditions of the different reduced Ti_3_C_2_ are shown in table S1. The mixture was then loaded into an alumina crucible and placed in a nitrogen-purged tube furnace. The furnace was heated to the target temperature (500 or 550°C/ min) at a rate of 5°C/ min and maintained at that temperature for 5 hours. After the reaction, the system was allowed to cool down naturally under nitrogen flow. The obtained product was subsequently treated with a dilute HCl solution to remove residual molten salts and metallic Na. The mixture was stirred continuously during washing to ensure the complete removal of impurities. The purified MXene was collected via vacuum filtration, rinsed multiple times with deionized water, and lastly dried under vacuum at 60°C for 12 hours. By adjusting the Ti_3_C_2_Cl*_x_*:Na molar ratio and the annealing temperature, a series of reduced Ti_3_C_2_ samples with different degrees of surface reduction were obtained for systematic investigation of their structural and photothermal properties.

### Intercalation of Ti_3_C_2_Cl*_x_*

To facilitate the exfoliation of Ti_3_C_2_Cl*_x_* into nanosheets, TBAOH was used as an intercalation agent. In a typical procedure, 0.5 g of Ti_3_C_2_Cl*_x_* powder was dispersed in a mixture of 10 ml of deionized water and 5 ml of 40 wt % TBAOH solution under vigorous magnetic stirring. The suspension was then transferred to a water bath maintained at 50°C and continuously stirred for 12 hours to ensure sufficient intercalation between TBA^+^ ions and the MXene interlayers. After the intercalation process, the resulting dispersion was centrifuged at moderate speed to remove the supernatant containing excess TBAOH and reaction by-products. The precipitate was subsequently redispersed in deionized water and centrifuged repeatedly until the pH of the supernatant reached neutrality, indicating the complete removal of residual alkaline species. Last, the intercalated Ti_3_C_2_Cl*_x_* product was collected via vacuum filtration and dried under vacuum at 60°C for 12 hours to obtain a freestanding powder, which was further used for exfoliation or structural characterization.

### Preparation of Ti_3_C_2_Cl*_x_* nanosheets

To minimize oxidative degradation of Ti_3_C_2_Cl*_x_* nanosheets during the exfoliation process, deionized water was first degassed by boiling under magnetic stirring for 30 min, followed by natural cooling to room temperature. Subsequently, 0.5 g of intercalated Ti_3_C_2_Cl*_x_* was dispersed into 100 ml of the degassed water under an ambient argon atmosphere. The dispersion was then subjected to ultrasonication using a probe-type ultrasonic processor with a power output of 300 W. The sonication was carried out in a pulsed mode (30 s on, 15 s off) for a total duration of 12 hours, while nitrogen gas was continuously introduced into the solution through a fine tube to generate bubbles, thereby further reducing the dissolved oxygen content and maintaining an inert environment throughout the process. The entire ultrasonication was performed under a −12°C cooling bath to prevent thermal degradation. After sonication, the resulting suspension was transferred to centrifuge tubes and centrifuged at 5000 rpm for 1 hour to remove unexfoliated multilayer particles. The upper portion of the supernatant, which contained few-layer or monolayer Ti_3_C_2_Cl*_x_* nanosheets, was carefully collected. Last, the obtained nanosheet dispersion was stored in sealed vials and kept at 4°C before use, ensuring long-term stability and minimal oxidation during storage.

### Preparation of reduced Ti_3_C_2_ nanosheets

To effectively preserve the reduced surface state of reduced Ti_3_C_2_ and mitigate oxidative degradation due to its high surface reactivity, the exfoliation process was carried out directly in an aqueous solution containing ascorbic acid. Before use, an ascorbic acid solution (2 mg/ml) was prepared by dissolving ascorbic acid in deionized water that had been degassed via boiling under magnetic stirring for 30 min, followed by natural cooling to room temperature under an argon atmosphere. A total of 0.5 g of reduced Ti_3_C_2_ was then added to 100 ml of the degassed ascorbic acid solution under argon protection. The mixture was subsequently subjected to ultrasonication using a probe-type ultrasonic processor operating at a power output of 300 W. The sonication was conducted in a pulsed mode (30 s on, 15 s off) for a total duration of 12 hours, with nitrogen gas continuously introduced into the solution through a fine tube to further reduce dissolved oxygen levels and maintain an inert environment throughout the process. The entire procedure was performed under a −12°C cooling bath to prevent thermal damage and oxidation. After ultrasonication, the resulting suspension was transferred into centrifuge tubes and centrifuged at 5000 rpm for 1 hour to remove unexfoliated multilayer particles. The upper portion of the supernatant, rich in few-layer or monolayer reduced Ti_3_C_2_ nanosheets, was carefully collected. Last, the obtained nanosheet dispersion was stored in sealed vials under an argon atmosphere and kept at 4°C before further use, ensuring long-term stability and minimal surface oxidation.

### Preparation of reduced Ti_3_C_2_ nanosheets dispersed in deionized water

To facilitate subsequent characterization and application studies, reduced Ti_3_C_2_ nanosheets were transferred from the ascorbic acid–containing dispersion into pure deionized water. In this process, the original nanosheet dispersion prepared in an ascorbic acid solution (2 mg/ml) was subjected to vacuum filtration using a Celgard 3501 microporous membrane. During filtration, large volumes of degassed deionized water were repeatedly added to the membrane to thoroughly rinse away residual ascorbic acid until the pH of the filtrate reached neutrality. Unlike conventional drying procedures, the filter cake containing the nanosheets was not dried to avoid compromising colloidal stability. Instead, the freshly filtered nanosheet aggregate was immediately redispersed into a desired volume of degassed deionized water. To ensure uniform redispersion and prevent aggregation, the suspension was further sonicated in an ice-water bath for 1 hour using a bath sonicator. The resulting stable aqueous dispersion of reduced Ti_3_C_2_ nanosheets was injected with nitrogen and then stored at 4°C for future use, maintaining both structural integrity and surface reduction state without interference from stabilizing agents.

### Concentration determination of MXene nanosheets

Because of the lack of a universally accepted standard curve for MXene materials and potential interference from surface terminations, it is challenging to accurately determine the concentration of MXene nanosheets using UV-Vis spectroscopy. Consequently, a gravimetric method was used to quantify the mass concentration of MXene nanosheets in aqueous dispersion. In a typical procedure, 20 ml of well-dispersed nanosheet solution was vacuum-filtered through a preweighed Celgard 3501 microporous monolayer membrane, which is known for its uniform pore structure and low background mass interference. Before filtration, the membrane was dried at 60°C under vacuum for 12 hours and accurately weighed using an analytical balance to obtain its initial mass (*m*_1_). After filtration, the membrane with retained nanosheets was dried again under the same conditions to ensure complete removal of residual water. The membrane was then cooled in a desiccator and reweighed to obtain the final mass (*m*_2_). The dry mass of the nanosheets was calculated as the difference between *m*_2_ and *m*_1_. The mass concentration of the nanosheet dispersion (*C*, in micrograms per milliliter) was determined using the following equation

$$C=\frac{m_{2}-m_{1}}{V}$$

where *V* represents the volume of the dispersion used for filtration (20 ml). This operation process involves three measurements and the average value is taken.

### Determination of the extinction coefficient

To assess the NIR absorption capacity of MXene nanosheets, their extinction coefficient ε(λ) was calculated using the Lambert-Beer law

A(λ)=ε(λ)·L·C

where *A*(λ) is the absorbance at wavelength λ, *L* is the optical path length (1 cm), and *C* is the concentration of the MXene nanosheets (in grams per liter). By measuring the absorbance of dispersions with varying concentrations at a given wavelength, a linear calibration curve of absorbance versus concentration was obtained. The slope of this line corresponds to ε(λ) · *L*, allowing the extinction coefficient ε(λ) to be determined from the slope divided by the path length (in liters per gram per centimeter).

### Photothermal performance evaluation of MXene nanosheets

To evaluate the photothermal performance of MXene nanosheets, a series of in vitro experiments was conducted using NIR laser irradiation.

For temperature elevation measurement, MXene nanosheets were dispersed in deionized water at various concentrations. Each dispersion was sonicated for 5 min to ensure uniform distribution of the nanosheets. Each nanosheet dispersion (1 ml) was placed in a 1.5-ml centrifuge tube and irradiated with an 808-nm NIR laser at a power density of 1.0 W/cm^2^. The laser power was calibrated by a power meter, which confirms that the laser power is 1.5 W. For achieving the power density of 1.0 W/cm^2^, the light spot area was set as 1.5 cm^2^. The temperature changes were monitored using an infrared thermal camera every 30 s for a total duration of 10 min. A control experiment without nanosheets was also performed to account for any background heating effects.

For calculation of PTCE, we first recorded the temperature rise and fall data of the solution and solvent (first heated to the highest temperature, then let it cool naturally to close to room temperature). We used deionized water as the solvent. The absorbance of the solution at a specific wavelength can be obtained from the UV-Vis results. Here, we used 808-nm NIR light (power: 1.5 W calibrated by a power meter), with a laser power density of 1 W/cm^2^, a laser power on the samples of 1044 mW, and 1 ml of deionized water as the solvent.

Calculation formula

$$\eta =\frac{\mathrm{hs}\left(T_{\text{Max}}-T_{\text{Sur}}\right)-Q_{\text{Dis}}}{I\left(1-10^{-A}\right)}$$

Here, η is the PTCE, *h* represents the heat transfer coefficient, *s* is the surface area of the container, *T*_Max_ is the highest temperature reached by the solution, *T*_Sur_ is the ambient temperature, *Q*_Dis_ is the heat loss, *I* is the incident laser power, and *A* is the absorbance.

$$\mathrm{hs}=\frac{m_{D}c_{D}}{\tau_{s}}$$

Here, *m*_D_ represents the mass of the solution, *c*_D_ represents the specific heat capacity of the solution, and τ is the system time constant. Because the mass of the solvent is much greater than that of the solute, we can approximately consider the mass of the solution to be the mass of the deionized water, i.e., *m* = 1 g, and the specific heat capacity of the solution is the specific heat capacity of the deionized water, i.e., *c*_D_ = 4.2 J/(g·°C).

To calculate the system time constant, the following formula is required

$$t=-\tau_{s}\mathrm{ln\theta}$$

$$\theta =\frac{T-T_{\text{Sur}}}{T_{\text{Max}}-T_{\text{Sur}}}$$

Here, *t* represents the cooling time, *T* denotes the temperature at different time points during the cooling process, *T*_Sur_ represents the ambient temperature, and *T*_Max_ represents the maximum temperature.

$$Q_{\text{Dis}}=h_{\text{water}}s_{\text{water}}\left(T_{\text{Max},\text{water}}-T_{\text{Sur}}\right)$$

Thus, the final formula for light-to-heat conversion can be simplified to

$$\eta =\frac{m_{D}c_{D}\left[\frac{\left(T_{\text{Max},\text{solvent}}-T_{\text{Sur}}\right)}{\tau_{s,\text{solvent}}}\right]-Q_{\text{Dis}}}{I\left(1-10^{-A}\right)}$$

### Gibbs free energy calculation

To assess the thermodynamic feasibility of the reduction process, Gibbs free energy changes (Δ*G*) for the reactions between TiCl_3_ and various reductants (Na, LiH, Al, Zn, Sn, Ga, H_2_, and NH_3_) were calculated using HSC Chemistry 6.0 software. This approach was used to model the reduction of Ti^3+^ to Ti^2+^ during the electron injection process in MXene materials. The general reaction can be expressed as

$${\text{TiCl}}_{3}+n\text{Reductant}\to {\text{TiCl}}_{2}+n\text{By-products}$$

In this reaction, Ti^3+^ in TiCl_3_ is reduced to Ti^2+^ in TiCl_2_, accompanied by the oxidation of the reductant and formation of corresponding by-products (e.g., NaCl, LiCl, AlCl_3_, etc.). The stoichiometry and nature of the by-products were determined based on known reaction pathways and thermodynamic data available in the HSC database. Gibbs free energy changes were calculated at elevated temperatures (400° to 600°C), which correspond to the experimental conditions used in the reduction of MXene. The calculation is based on the thermodynamic relation

ΔG=ΔH−TΔS

where Δ*H* is the enthalpy change, *T* is the absolute temperature, and Δ*S* is the entropy change of the system. Reactions with more negative Δ*G* values indicate higher thermodynamic favorability for the reduction of Ti^3+^ to Ti^2+^ and thus greater potential for effective electron injection into the MXene lattice. These results provided a theoretical foundation for selecting metallic Na as the most suitable reductant, not only because of its highly negative Δ*G* value but also because of its compatibility with maintaining the structural integrity of MXene during the reduction process.
