# Supplementary material for: Surface reduction boosts free electron concentration in MXene for enhanced photothermal performance
Source: Sci Adv. 2026 May 13;12(20):eaee2009. doi: 10.1126/sciadv.aee2009 (PMC13170662; doi:10.1126/sciadv.aee2009)
Supplement: Supplementary file 1 — Supplementary Text Figs. S1 to S61 Tables S1 to S14 Legends for movies S1 and S2 [file sciadv.aee2009_sm.pdf]

Science Advances
AAAS

Supplementary Materials for

Surface reduction boosts free electron concentration in MXene for enhanced photothermal performance

Haoming Ding *et al.*

Corresponding author: Yong Zhang, yozhang@cityu.edu.hk

Sci. Adv. **12**, eaee2009 (2026)
DOI: 10.1126/sciadv.aee2009

The PDF file includes:

Supplementary Text
Figs. S1 to S61
Tables S1 to S14
Legends for movies S1 and S2

Other Supplementary Material for this manuscript includes the following:

Movies S1 and S2

Supplementary Text

Cell Culture and Treatment

This study utilized Human Umbilical Vein Endothelial Cells (HUVECs) (Sigma-Aldrich, SCCE001) and murine RAW 264.7 cells (Wuhan Pricella Biotechnology Co., Ltd., CL-0190) to evaluate the biocompatibility of MXenes. Both cell lines were cultured in Dulbecco's Modified Eagle Medium (DMEM) supplemented with 10% fetal bovine serum (FBS) and 1% penicillin-streptomycin (P/S). The cells were maintained at 37°C in a humidified atmosphere containing 5% CO_2.

Cytotoxicity Assay

To assess the cytotoxicity of MXene, HUVECs and RAW 264.7 cells were seeded at a density of 5×10^3 cells per well in 96-well plates and incubated for 24 hours at 37°C and 5% CO_2 to allow cell attachment. After attachment, the medium was replaced with fresh DMEM containing various concentrations of MXene for the experimental groups, while control groups received plain DMEM without MXene. Cells were further incubated for 24 and 48 hours. For viability assessment, 10 μL of the Cell Counting Kit-8 (CCK-8, Beyotime) reagent was added to each well and incubated at 37°C in the dark for 30 minutes. Absorbance was measured at 450 nm using a microplate reader. All experiments were conducted with three independent biological replicates. For each biological replicate, samples were assayed in triplicate (technical replicates) to ensure measurement precision. Cell viability was calculated based on the absorbance values to evaluate the impact of MXene on cell activity. Statistical analyses were performed using GraphPad Prism 9.4.1. Data conforming to a normal distribution are expressed as mean ± standard deviation (SD). Comparisons between multiple groups were performed using one-way analysis of variance (ANOVA) with Tukey's post-hoc test. Data not conforming to a normal distribution were analyzed using the Mann-Whitney U test. A value of $P < 0.05$ was considered statistically significant.

Live/Dead Cell Staining

Cell viability was also assessed using the Calcein AM/Propidium Iodide (PI) staining kit (Beyotime). Calcein AM penetrates live cells and is hydrolyzed by intracellular esterases to produce green fluorescence, while PI only enters cells with compromised membranes, binding to nucleic acids to emit red fluorescence. Briefly, after treatment with different concentrations of MXene for the designated periods, cells were gently washed three times with phosphate-buffered saline (PBS) to remove residual medium and unbound materials. A staining solution was prepared by mixing Calcein AM, PI, and buffer at a ratio of 1:1:1000 and added to the wells. Cells were then incubated at 37°C in the dark for 30 minutes. Fluorescence microscopy was used to visualize and capture images of the stained cells under appropriate excitation wavelengths, allowing for the analysis of cell viability and morphology. ImageJ software was used to calculate the fluorescence intensity of live and dead cells. All experiments were performed with three independent biological replicates. For each biological replicate, at least three representative fields of view were analyzed per condition (technical replicates) to ensure the representativeness of the results. Statistical analyses were performed using GraphPad Prism 9.4.1. Data conforming to a normal distribution are expressed as mean ± standard deviation (SD). Comparisons between multiple groups were

performed using one-way ANOVA with Tukey's post-hoc test. Data not conforming to a normal distribution were analyzed using the Mann-Whitney U test. A value of $P < 0.05$ was considered statistically significant.

Preparation of woundplasts loaded with different materials

To systematically investigate the influence of material type and loading concentration on photothermal performance, woundplasts were prepared by loading commercial medical adhesive dressings with reduced Ti_3C_2 (1:8, 550°C), pristine $Ti_3C_2Cl_x$, or AuNPs at gradient concentrations. Briefly, the original woundplasts were cut into standardized square units measuring $1 \times 1\ cm^2$ to ensure uniformity across all experimental groups. These substrates were then immersed in aqueous dispersions of each nanomaterial at four different concentrations: 5, 10, 15, and 20 μg/mL. The pristine woundplasts were immersed in the aqueous solutions containing different materials with different concentrations, allowing sufficient adsorption of the nanomaterials onto the fibrous surface of the woundplast. Following this, all samples were dried under vacuum at 60°C for 24 hours to remove residual moisture, prevent oxidation of MXene species, and ensure stable integration of the materials onto the substrate. After drying, the resulting woundplasts were stored in a desiccator under low-temperature conditions before further characterization. This preparation protocol not only ensured consistent loading across samples but also enabled a direct comparison of photothermal efficiency as a function of both material composition and concentration, laying the foundation for subsequent antimicrobial evaluations.

Antibacterial Experiment

Bacterial Strain Activation and Dilution. The frozen *Staphylococcus aureus* strain was inoculated into Luria-Bertani (LB) broth and activated by incubation at 37°C with shaking at 220 rpm for 16 hours. Following activation, the bacterial suspension was serially diluted using sterile saline via the 10-fold gradient dilution method to prepare a series of suspensions with different concentrations. An appropriate dilution was selected for the subsequent photothermal antibacterial.

Photothermal Antibacterial and Colony-Forming Unit (CFU) Assay. An appropriate volume of *Staphylococcus aureus* bacterial (Biofeng, ATCCBAA-977) suspension was added onto woundplasts loaded with different materials from the respective experimental groups. The samples were then subjected to photothermal treatment using an 808 nm laser at a power density of $1\ W/cm^2$ for 10 minutes. After irradiation, each woundplast was transferred into a tube containing bacterial culture medium. To ensure complete release of bacteria from the woundplast, the samples were vortexed thoroughly using a vortex mixer. For live/dead staining, 1 μL of staining working solution (C2030S, Beyotime) was added to 100 μL bacterial suspension and incubated at 37°C for 15 min in the dark. After incubation, 10 μL of suspension was observed under a fluorescence microscope. DMAO (green, Ex/Em = 503/530 nm) stained live bacteria, while PI (red, Ex/Em = 535/617 nm) stained dead bacteria. Subsequently, 100 μL of each treated bacterial suspension was uniformly spread onto the surface of LB agar plates using a Z-shaped streaking method to achieve even distribution. The inoculated plates were incubated upside-down in a constant-temperature incubator at 37°C for 16 hours. Following incubation, the number of colony-forming units on each plate was counted using ImageJ software. All photothermal antibacterial and CFU count experiments were performed with three independent biological replicates. For each biological

replicate, technical replicates were performed in triplicate to ensure measurement precision. Statistical analyses were performed using GraphPad Prism 9.4.1. Data conforming to a normal distribution are expressed as mean ± standard deviation (SD). Comparisons between multiple groups were performed using one-way ANOVA with Tukey's post-hoc test. Data not conforming to a normal distribution were analyzed using the Mann-Whitney U test. A value of $P < 0.05$ was considered statistically significant.

Bacterial Growth Curve Measurement. To evaluate the effect of different photothermal treatments on bacterial growth, 100 µL aliquots of the treated *Staphylococcus aureus* suspensions were inoculated into 96-well microplates containing 900 µL of fresh LB broth per well. The microplates were sealed with sterile breathable film to prevent evaporation and cross-contamination during incubation. The plates were incubated at 37°C in a microplate reader with continuous orbital shaking at 220 rpm. At hourly intervals over 12 hours, bacterial density was measured by reading the optical density at 600 nm (OD_{600}) using the microplate reader. Uninoculated LB broth was used as the blank control to subtract background absorbance. All experimental groups were tested in triplicate wells to ensure statistical reliability. The OD_{600} values were recorded over time and plotted against incubation time to construct bacterial growth curves. Growth curve experiments were performed with three independent biological replicates, and each condition was tested in triplicate technical replicates within each biological replicate.

Experiment and Characterization Instruments.

The varying samples were synthesized using a Nabertherm tabletop Tube Furnace. The morphological features of the as-prepared samples were investigated using a field emission scanning electron microscope (FEI Quanta 250 Environmental SEM) equipped with an energy-dispersive X-ray spectroscopy (EDS) system. Structural characterization was carried out via X-ray diffraction (XRD) on a Bruker D2 Phaser with LYNXEYE-T detector using Cu Kα radiation. For transmission electron microscopy (TEM) observations, low-magnification imaging and elemental analysis were performed using a FEI Talos 200S TEM/EDX system. High-resolution TEM (HR-TEM) and selected area electron diffraction (SAED) analyses were also conducted on the same instrument. For atomic-scale structural analysis, scanning transmission electron microscopy (STEM) was performed using a spherical aberration-corrected FEI Titan G2 60-300 microscope operated at 300 kV, which was also equipped with an EDX system for compositional mapping. X-ray photoelectron spectroscopy (XPS) measurements were carried out on a Thermo SCIENTIFIC Nexsa spectrometer with a monochromatic Al Kα X-ray source. The binding energy (BE) scale was calibrated by setting the C 1s peak to 284.8 eV for reference. To analyze the surface functional groups of MXene materials, Raman spectroscopy was employed using a Renishaw inVia Raman microscope. This technique allows for the identification of specific terminal groups present on the MXene surface, providing insights into their chemical composition and structural characteristics. The Raman spectra were acquired using a laser excitation wavelength of 633 nm, with appropriate power settings to avoid sample damage. The hydrodynamic size and surface charge (zeta potential) of the MXene were measured using a Malvern Zetasizer Pro Particle Analyser. Cell and bacterial viability assays were evaluated using a Molecular Devices SpectraMax M5e Microplate Reader. The optical absorption spectrum of MXene nanomaterials was recorded using an Agilent Cary 3500 Multicell UV-Vis spectrophotometer. Immunofluorescence experiments were conducted on a Nikon TS-100F fluorescence microscope.

Photothermal experiments were conducted under 808 nm continuous laser irradiation. The temperature measurement was recorded using a thermal imaging camera (HIKMICRO, K09). Hall effect measurements were carried out using a Physical Property Measurement System (PPMS-9, Quantum Design) equipped with a resistivity option. The electrical transport properties were characterized using a four-point probe configuration, with a KEITHLEY 2400 used as the current source and a KEITHLEY 2182 as the voltage meter. The measurement was controlled, and data were collected through a custom-built program in LabVIEW. For the Electron Paramagnetic Resonance (EPR) test in this study, the Bruker EMXplus-6/1 spectrometer produced by Germany was used. For the Fourier Transform Infrared Spectroscopy (FTIR) test, the Nicolet iN10 spectrometer manufactured in the United States was adopted. Ti K-edge analysis was performed with Si(111) crystal monochromators at the BL14W1 beamlines at the Shanghai Synchrotron Radiation Facility (SSRF) (Shanghai, China).

Theoretical Calculation

The initial crystal structure was obtained from the Crystallographic Information File (CIF) of the experimental crystal. The structure was then fully geometry-optimized using the Quantum ESPRESSO software package (version 7.3.1) under the framework of density functional theory (DFT). Electron-ion interactions were described using scalar-relativistic, optimized norm-conserving Vanderbilt (ONCV) pseudopotentials. A kinetic energy cutoff of 80 Ry was applied for the expansion of the Kohn–Sham wavefunctions in the plane-wave basis set. Exchange-correlation effects were treated within the generalized gradient approximation (GGA), using the Perdew-Burke-Ernzerhof (PBE) functional.

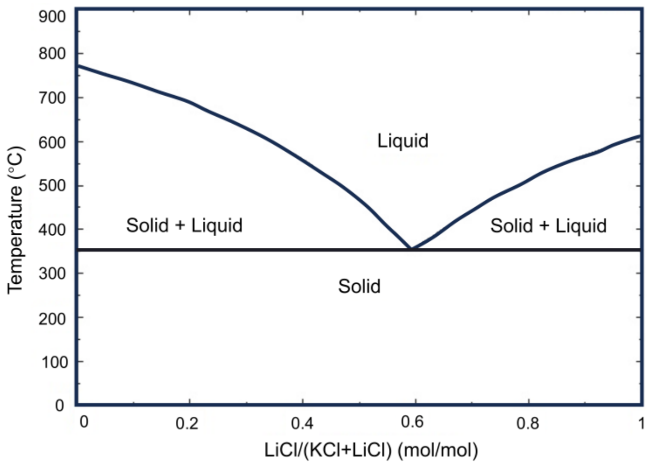

Fig. S1.
Salt phase diagrams of LiCl-KCl.

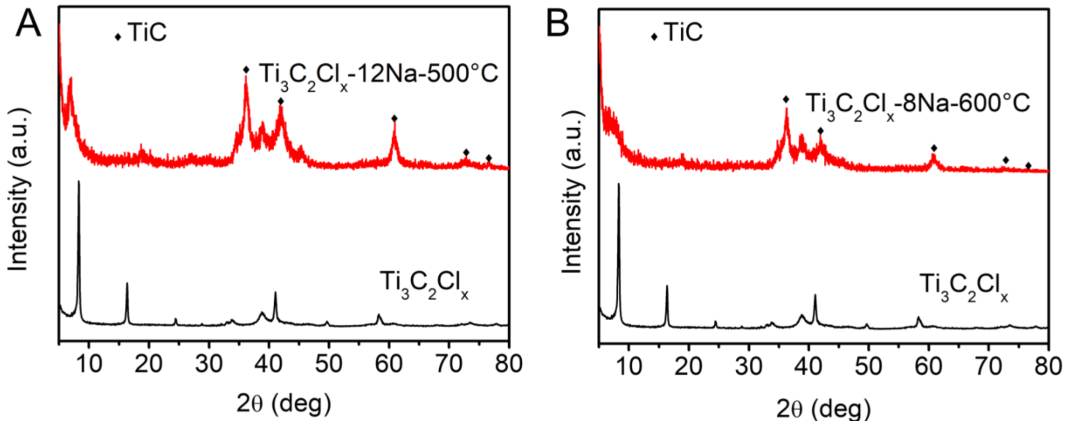

Fig. S2.
XRD characterization of reduced MXene with unsuitable reaction conditions. (A) XRD patterns of pristine $Ti_3C_2Cl_x$ and the product obtained from the reaction between $Ti_3C_2Cl_x$ and Na with a molar ratio of 1:12 at 500°C, showing the structural degradation after the reduction reaction under such conditions. **(B)** XRD patterns of pristine $Ti_3C_2Cl_x$ and the product obtained from the reaction between $Ti_3C_2Cl_x$ and Na with a molar ratio of 1:8 at 600°C, also showing the structural degradation after the reduction reaction under such conditions.

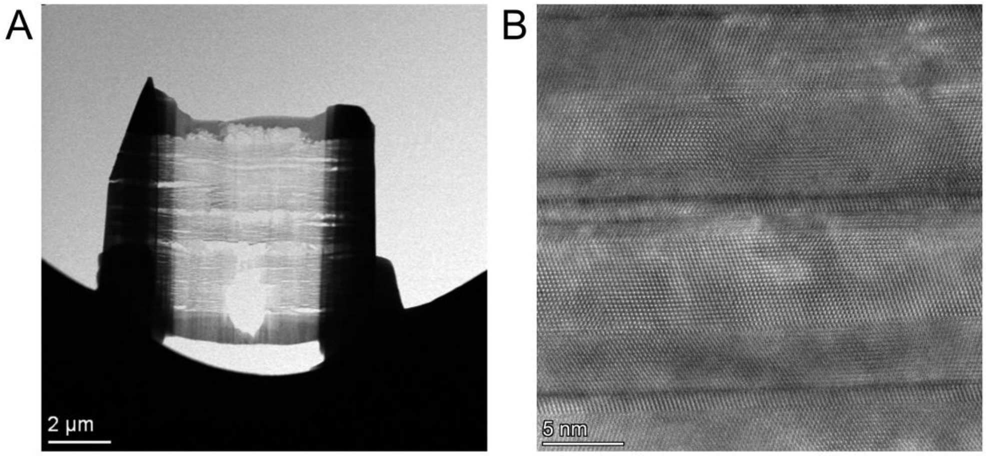

Fig. S3.
STEM characterization of the product obtained from the reaction of $Ti_3C_2Cl_x$ and Na with a molar ratio of 1:8 at 600°C. (A) Focused ion beam (FIB) cross-sectional image, showing the layered morphology despite the phase transformation. **(B)** Corresponding high-resolution STEM image, revealing the transformation of the MXene structure into crystalline TiC.

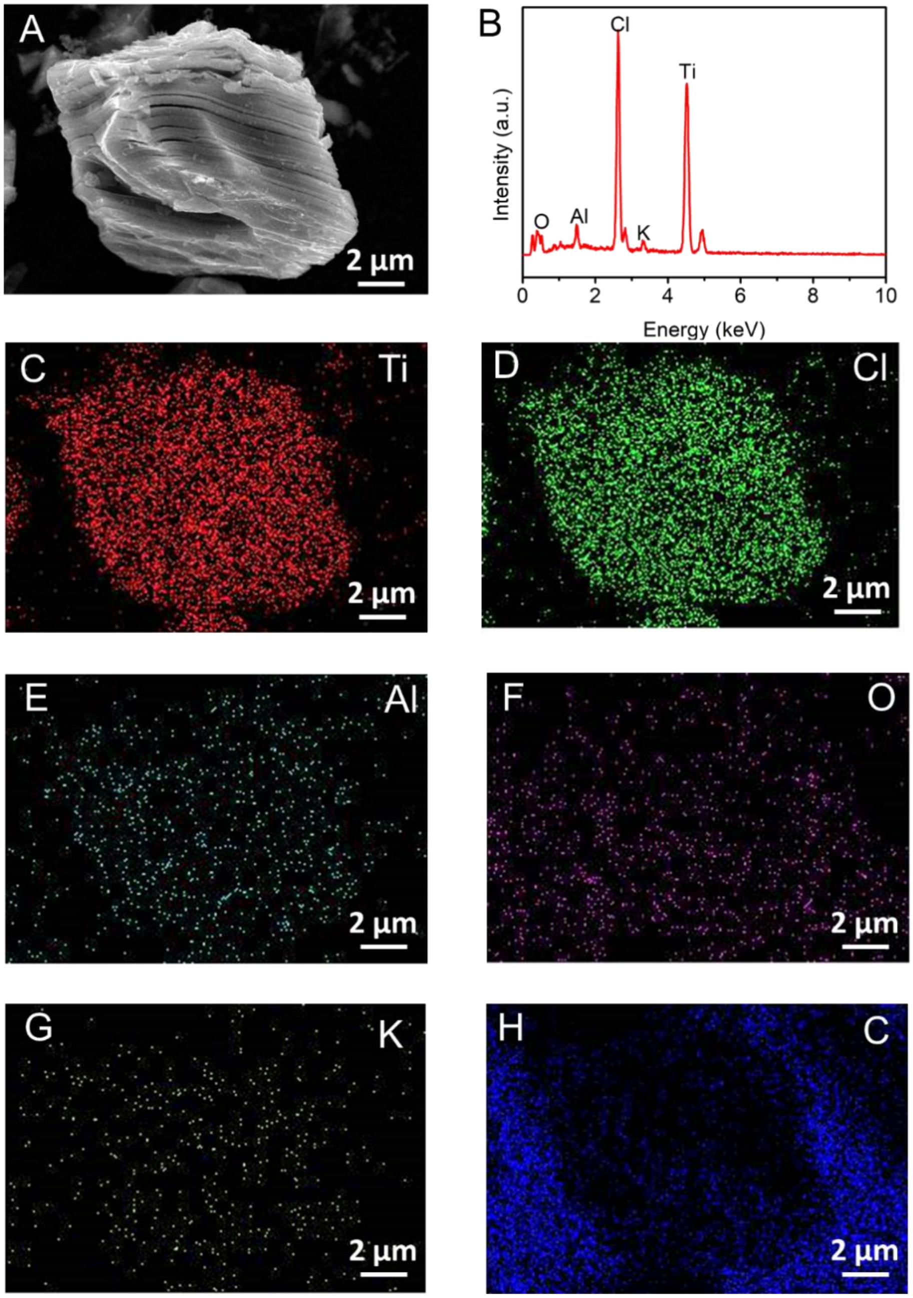

Fig. S4.

SEM characterization of pristine $Ti_3C_2Cl_x$ particle. (A) SEM image of the pristine $Ti_3C_2Cl_x$ particle. **(B)** SEM-EDS spectrum of pristine $Ti_3C_2Cl_x$ particle. SEM-EDS mappings of pristine $Ti_3C_2Cl_x$ particle, showing the elemental signal distribution of Ti **(C)**, Cl **(D)**, Al **(E)**, O **(F)**, K **(G)**, and C **(H)**.

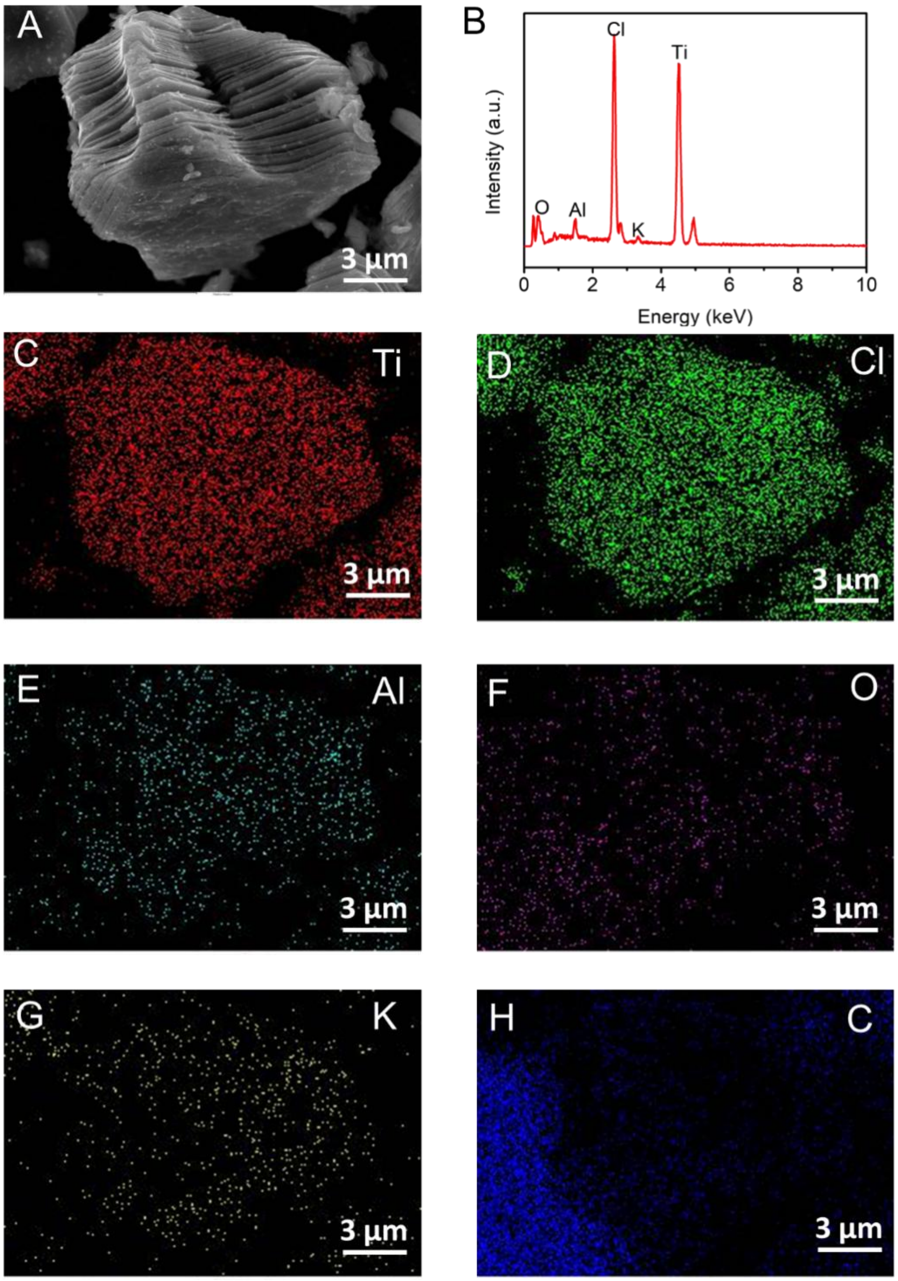

Fig. S5.

SEM characterization of annealed $Ti_3C_2Cl_x$ particle. (A) SEM image of an annealed $Ti_3C_2Cl_x$ particle. **(B)** SEM-EDS spectrum of annealed $Ti_3C_2Cl_x$ particle. SEM-EDS mappings of pristine $Ti_3C_2Cl_x$ particle, showing the elemental signal distribution of Ti **(C)**, Cl **(D)**, Al **(E)**, O **(F)**, K **(G)**, and C **(H)**.

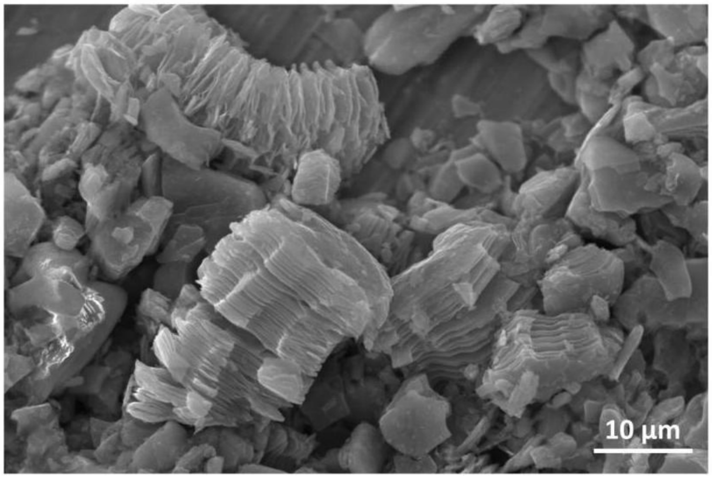

Fig. S6.
SEM image of reduced Ti_3C_2 (1:8, 550°C) particle, showing the exfoliated layer morphology after reduction reaction.

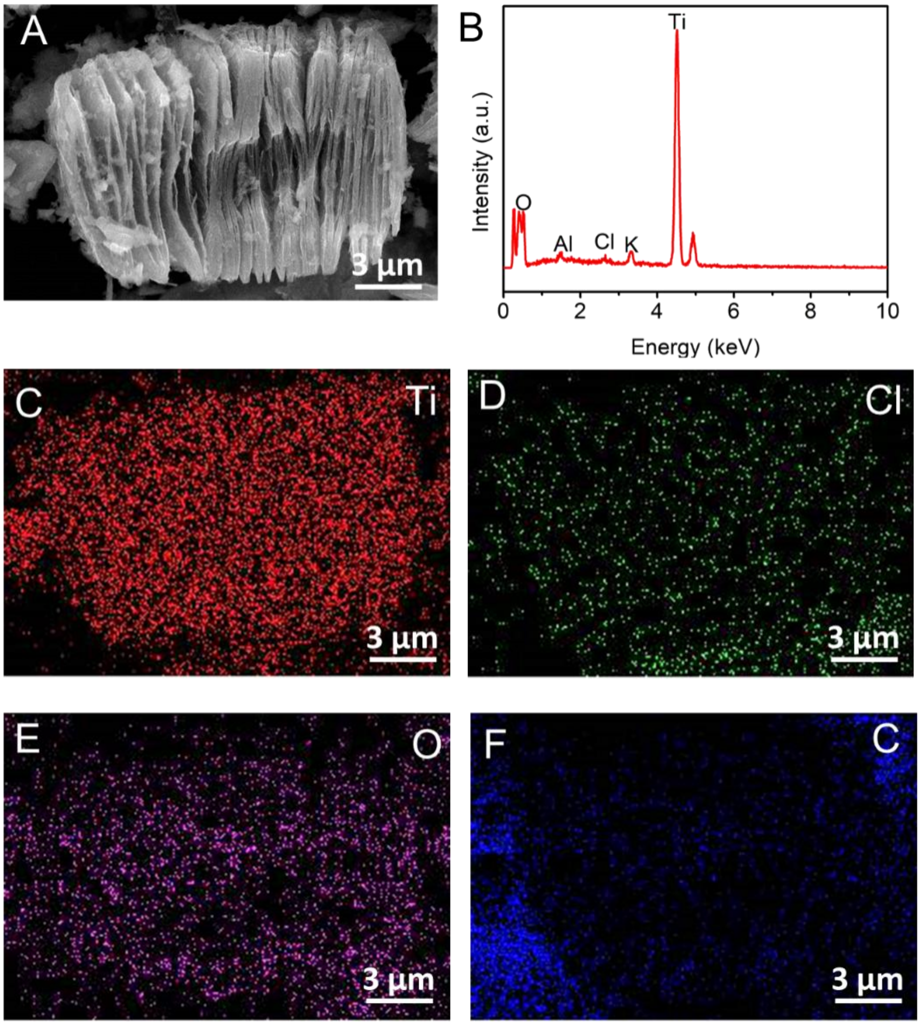

Fig. S7.

SEM characterization of reduced Ti_3C_2 (1:2, 500°C) particle. (A) SEM image of reduced Ti_3C_2 (1:2, 500°C) particle. **(B)** SEM-EDS spectrum detected from reduced Ti_3C_2 (1:2, 500°C) particle. SEM-EDS mappings of reduced Ti_3C_2 (1:2, 500°C) particle, showing the elemental signal distribution of Ti **(C)**, Cl **(D)**, O **(E)**, and C **(F)**.

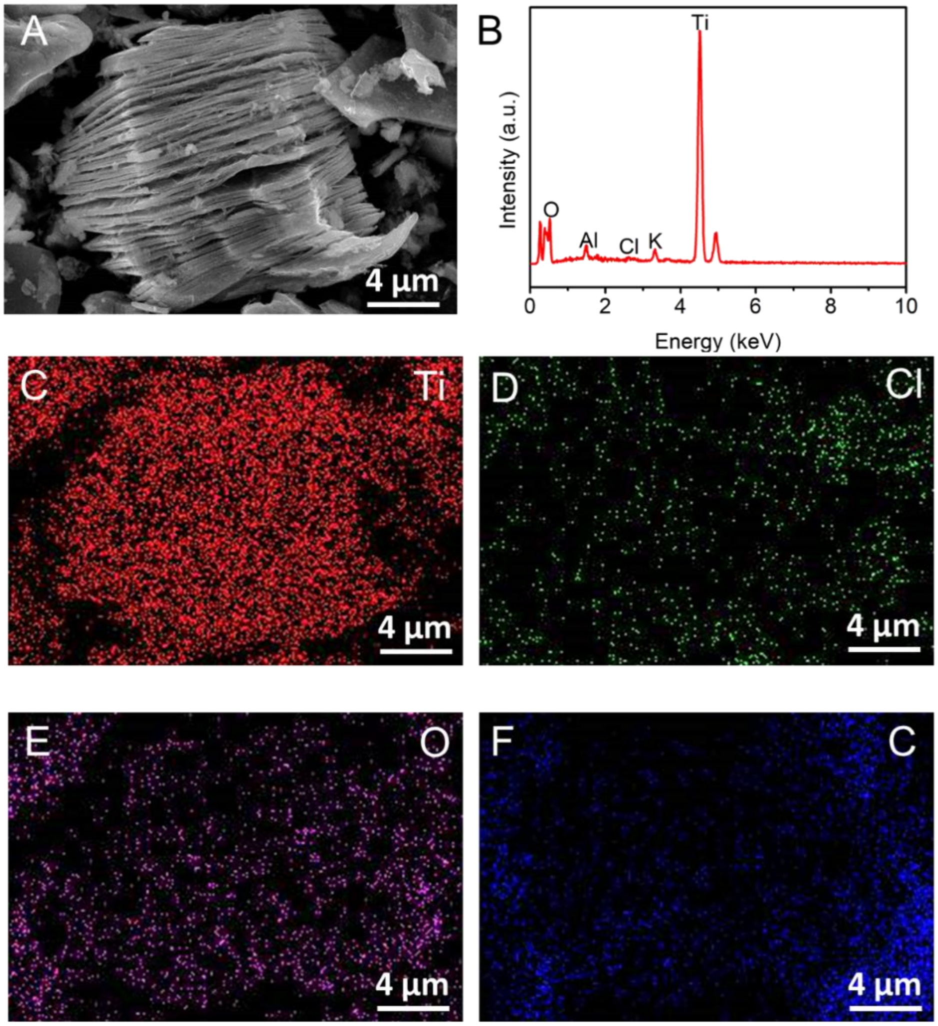

Fig. S8.
SEM characterization of reduced Ti_3C_2 (1:8, 500°C) particle. (A) SEM image of reduced Ti_3C_2 (1:8, 500°C) particle. **(B)** SEM-EDS spectrum detected from reduced Ti_3C_2 (1:8, 500°C) particle. SEM-EDS mappings of reduced Ti_3C_2 (1:8, 500°C) particle, showing the elemental signal distribution of Ti **(C)**, Cl **(D)**, O **(E)**, and C **(F)**.

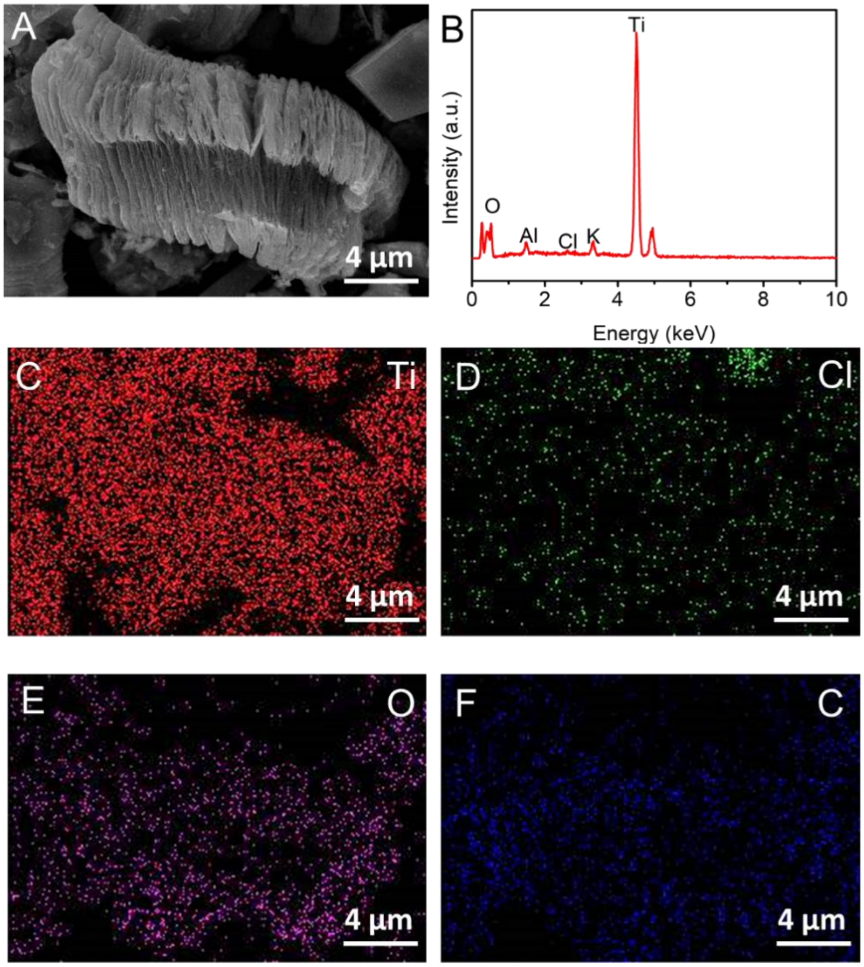

Fig. S9.

SEM characterization of reduced Ti_3C_2 (1:8, 550°C) particle. (A) SEM image of reduced Ti_3C_2 (1:8, 550°C) particle. **(B)** SEM-EDS spectrum detected from reduced Ti_3C_2 (1:8, 550°C) particle. SEM-EDS mappings of reduced Ti_3C_2 (1:8, 550°C) particle, showing the elemental signal distribution of Ti **(C)**, Cl **(D)**, O **(E)**, and C **(F)**.

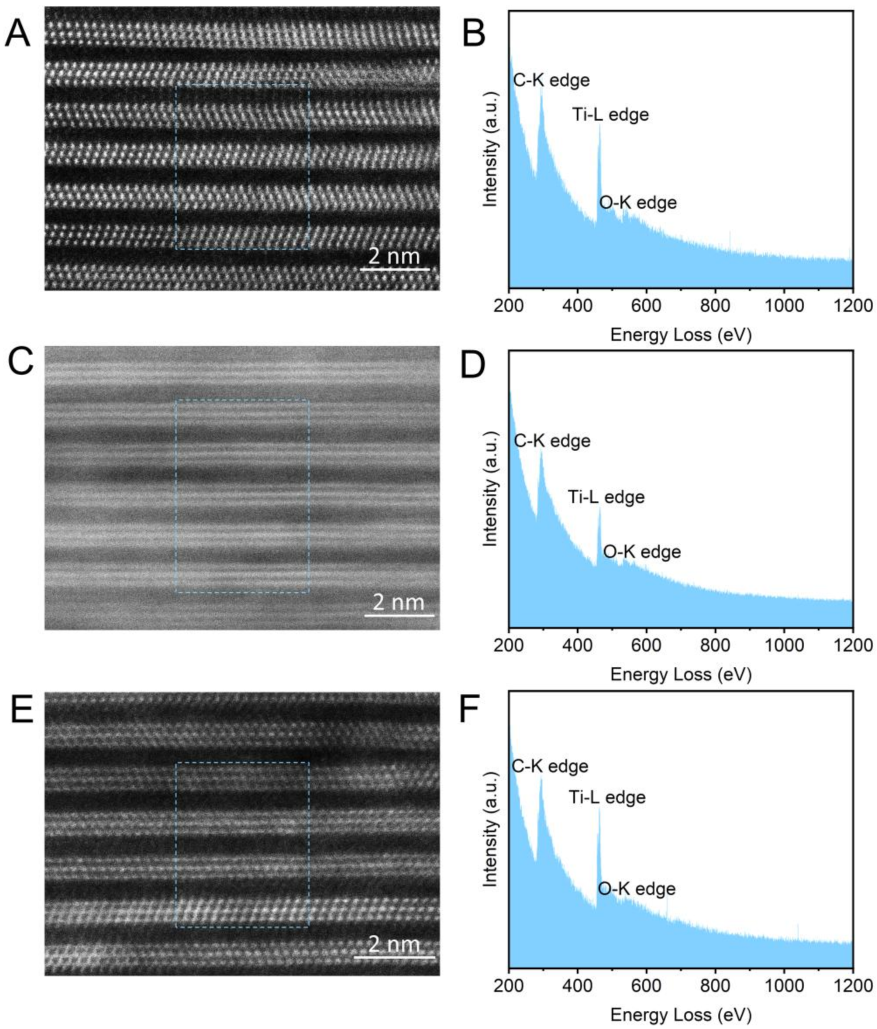

Fig. S10.

STEM characterization of different reduced MXenes. Atomic-resolution STEM images and their corresponding energy loss (EELS) spectra of reduced Ti_3C_2 (1:2, 500°C) **(A-B)**, reduced Ti_3C_2 (1:8, 500°C) **(C-D)**, and reduced Ti_3C_2 (1:8, 550°C) **(E-F)**.

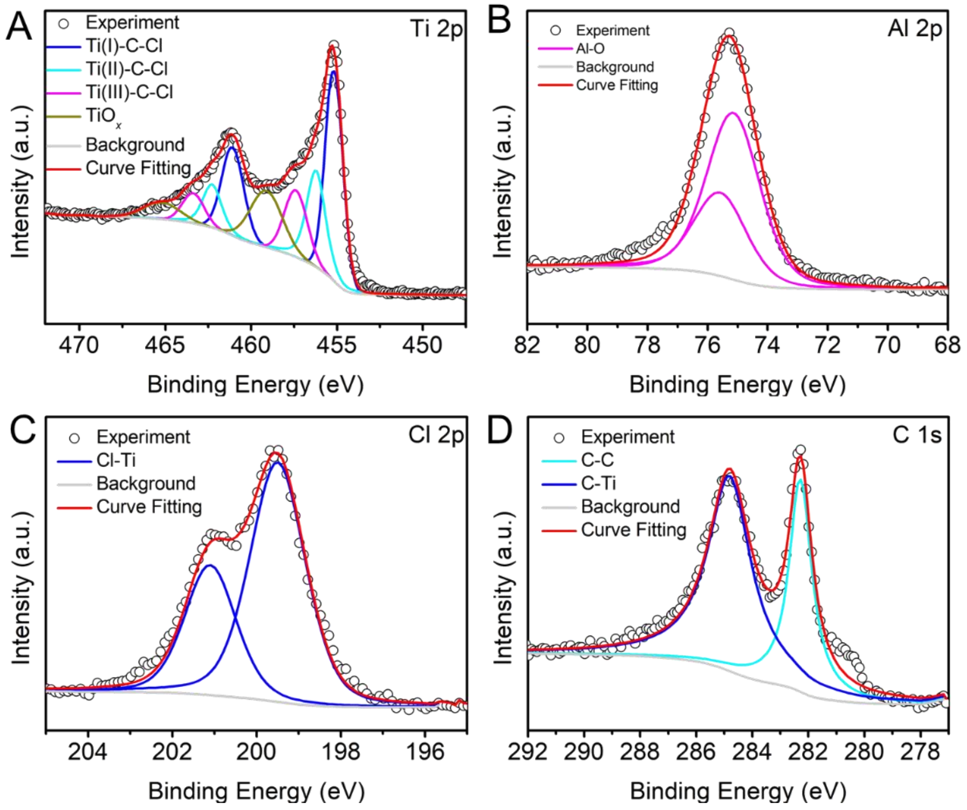

Fig. S11.
XPS analysis of pristine $Ti_3C_2Cl_x$. XPS fitting results of Ti 2p **(A)**, Al 2p **(B)**, Cl 2p **(C)**, and C 1s **(D)**.

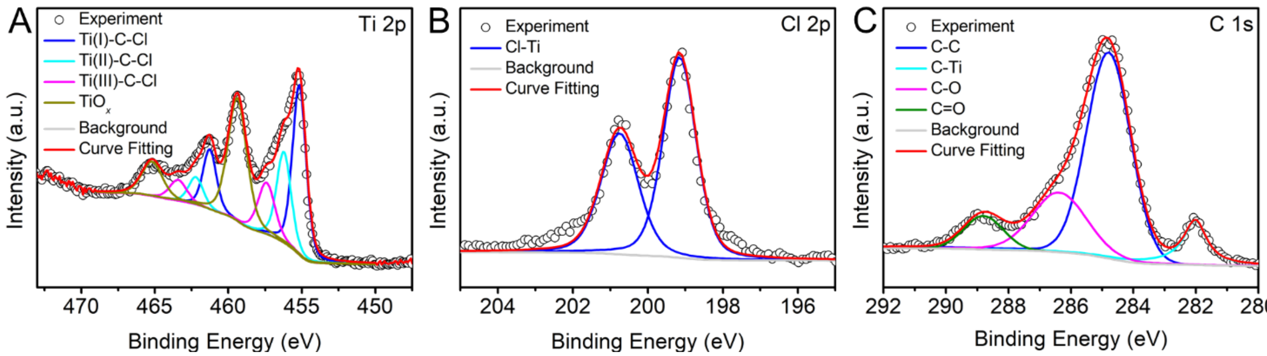

Fig. S12.

XPS analysis of annealed $Ti_3C_2Cl_x$. XPS fitting results of Ti 2p **(A)**, Cl 2p **(B)**, and C 1s **(C)**.

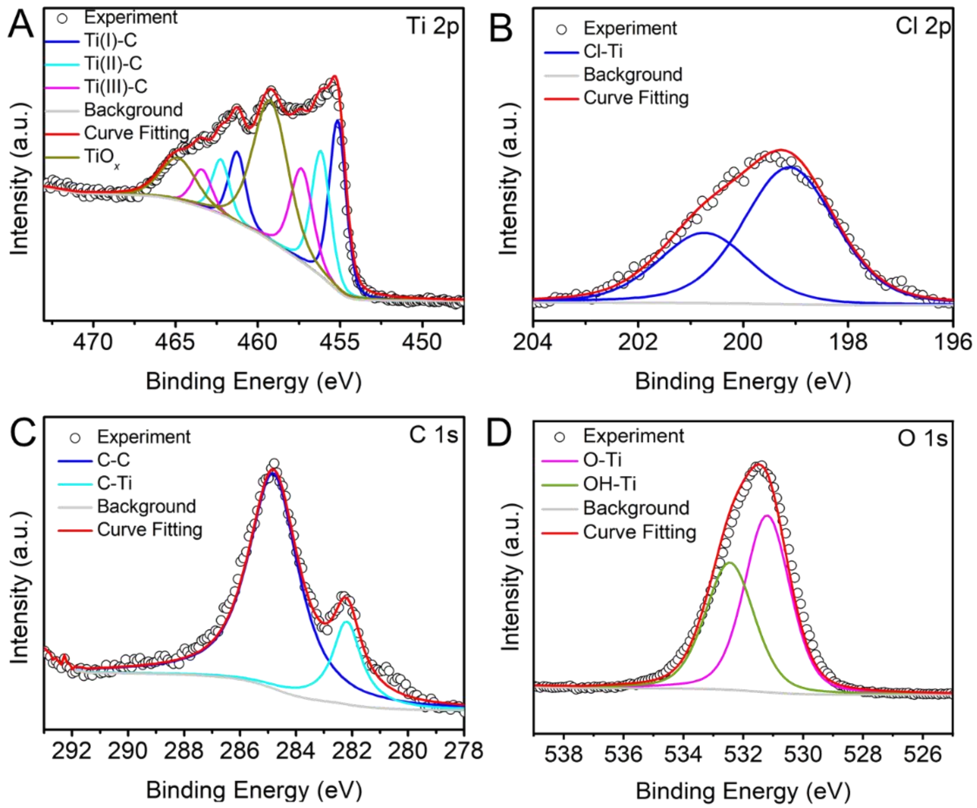

Fig. S13.
XPS analysis of reduced Ti_3C_2 (1:2, 500°C). XPS fitting results of Ti 2p **(A)**, Cl 2p **(B)**, C 1s **(C)**, and O 1s **(D)**. It is worth noting that the signals of Cl may be attributed to the trace residual on the MXene surface, which will not be detected in the reduced MXene nanosheets.

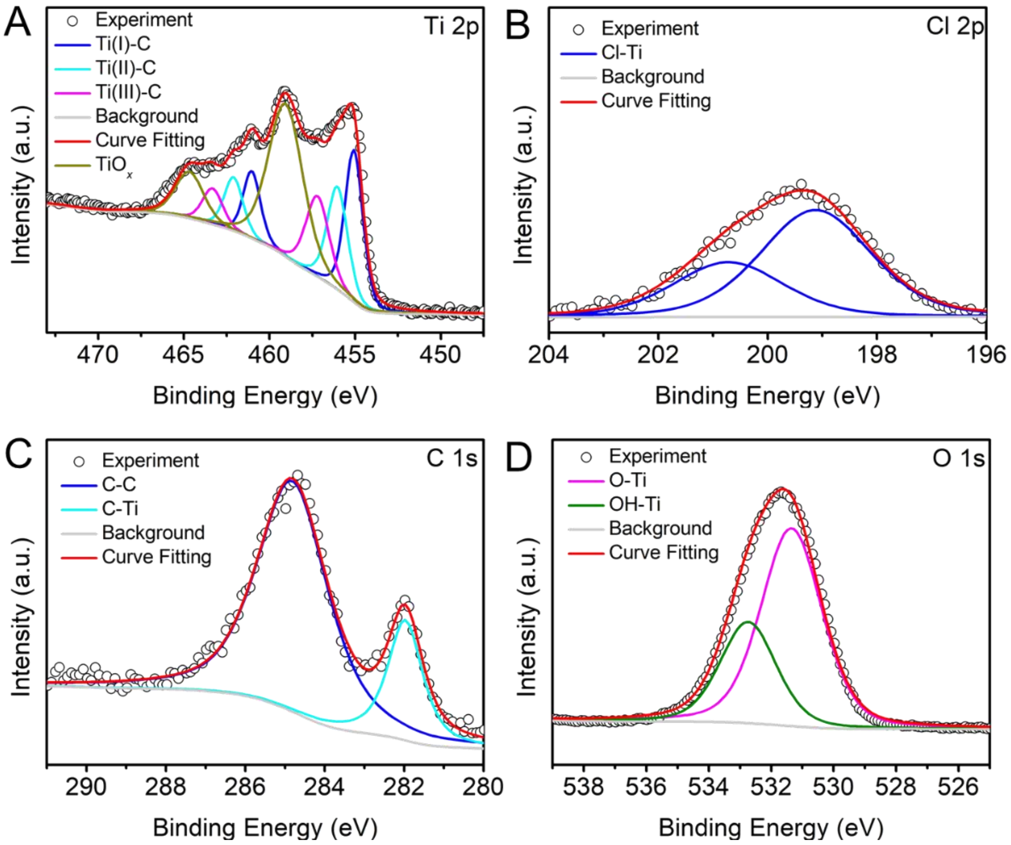

Fig. S14.
XPS analysis of reduced Ti_3C_2 (1:8, 500°C). XPS fitting results of Ti 2p **(A)**, Cl 2*p* **(B)**, C 1s **(C)**, and O 1s **(D)**. It is worth noting that the signals of Cl may be attributed to the trace residual on the MXene surface, which will not be detected in the reduced MXene nanosheets.

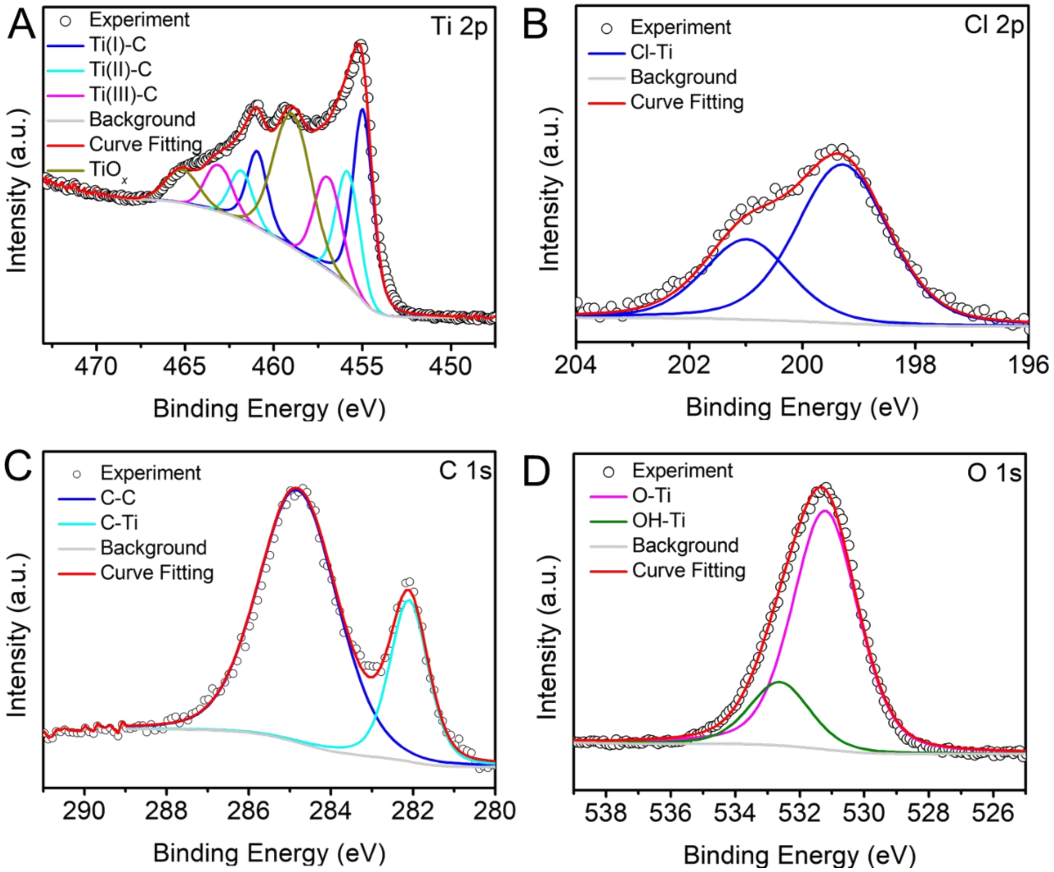

Fig. S15.

XPS analysis of reduced Ti_3C_2 (1:8, 550°C). XPS fitting results of Ti 2p **(A)**, Cl 2p **(B)**, C 1s **(C)**, and O 1s **(D)**. It is worth noting that the signals of Cl may be attributed to the trace residual on the MXene surface, which will not be detected in the reduced MXene nanosheets.

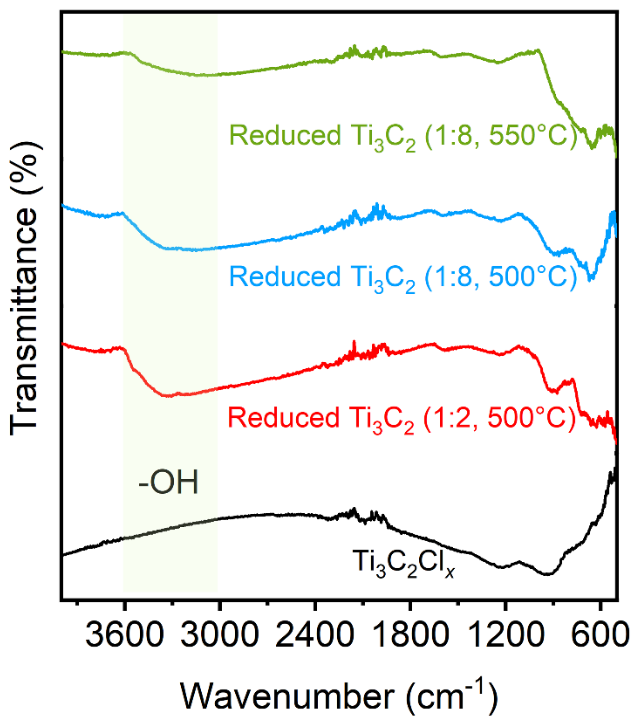

Fig. S16.
FTIR spectra of different MXenes.

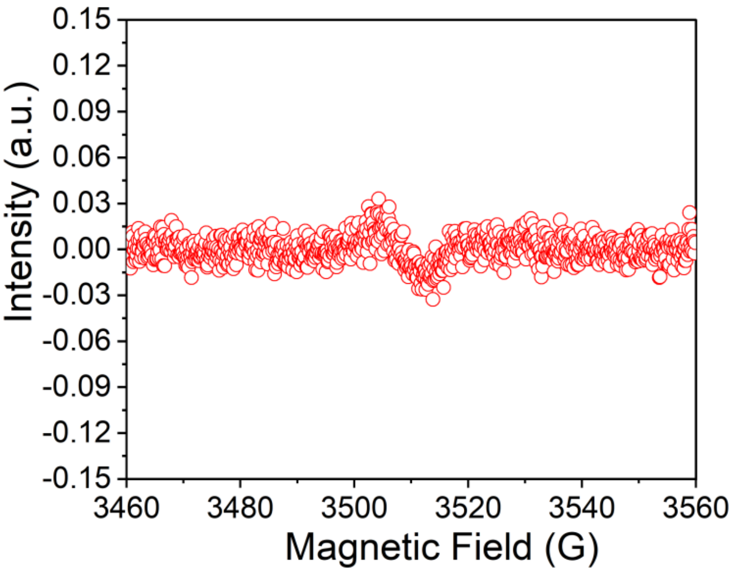

Fig. S17.
EPR spectrum of reduced Ti_3C_2 (1:8, 550°C) powder.

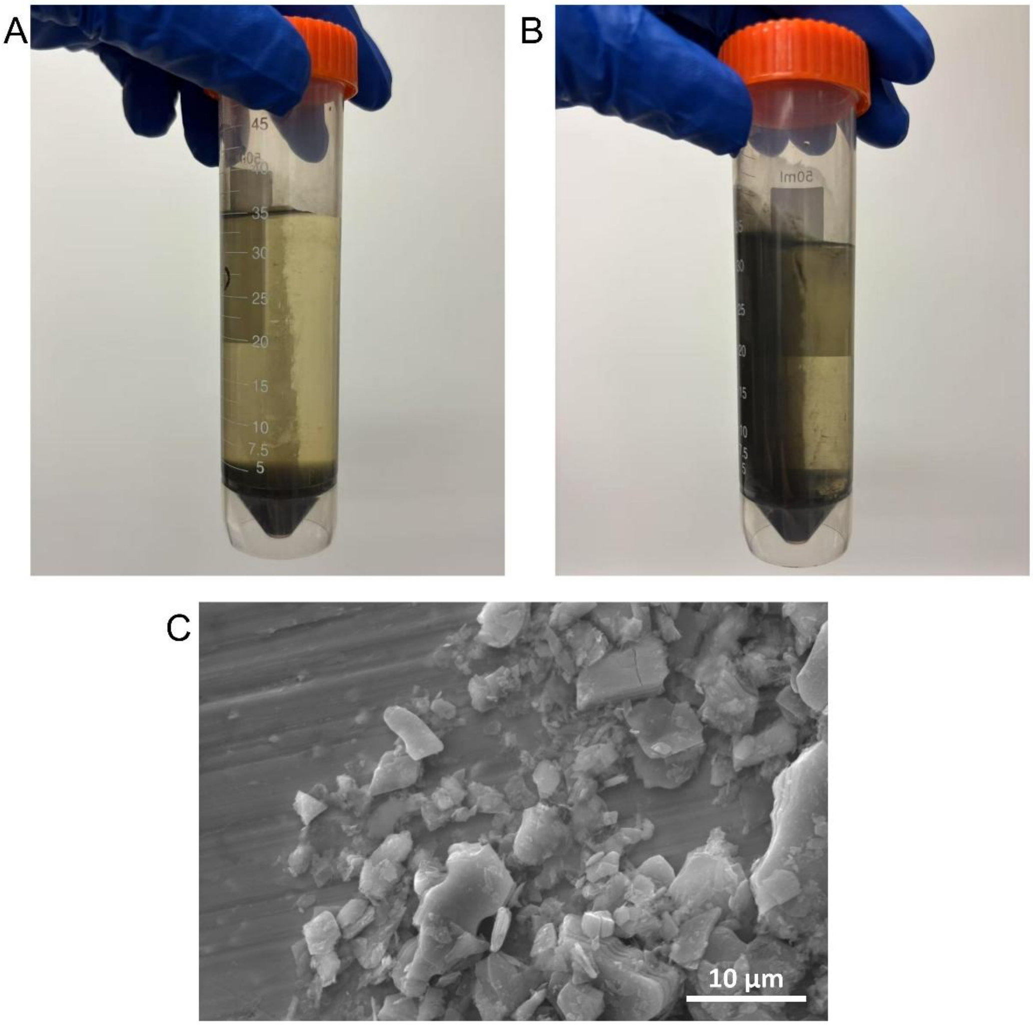

Fig. S18.

Characterization of reduced MXenes after sonication. Optical photographs of sonicated **(A)** reduced Ti_3C_2 (1:2, 500°C) and **(B)** reduced Ti_3C_2 (1:8, 500°C) after centrifugation at 5000 rpm for 1h. **(C)** SEM image of sonicated reduced Ti_3C_2 (1:8, 500°C).

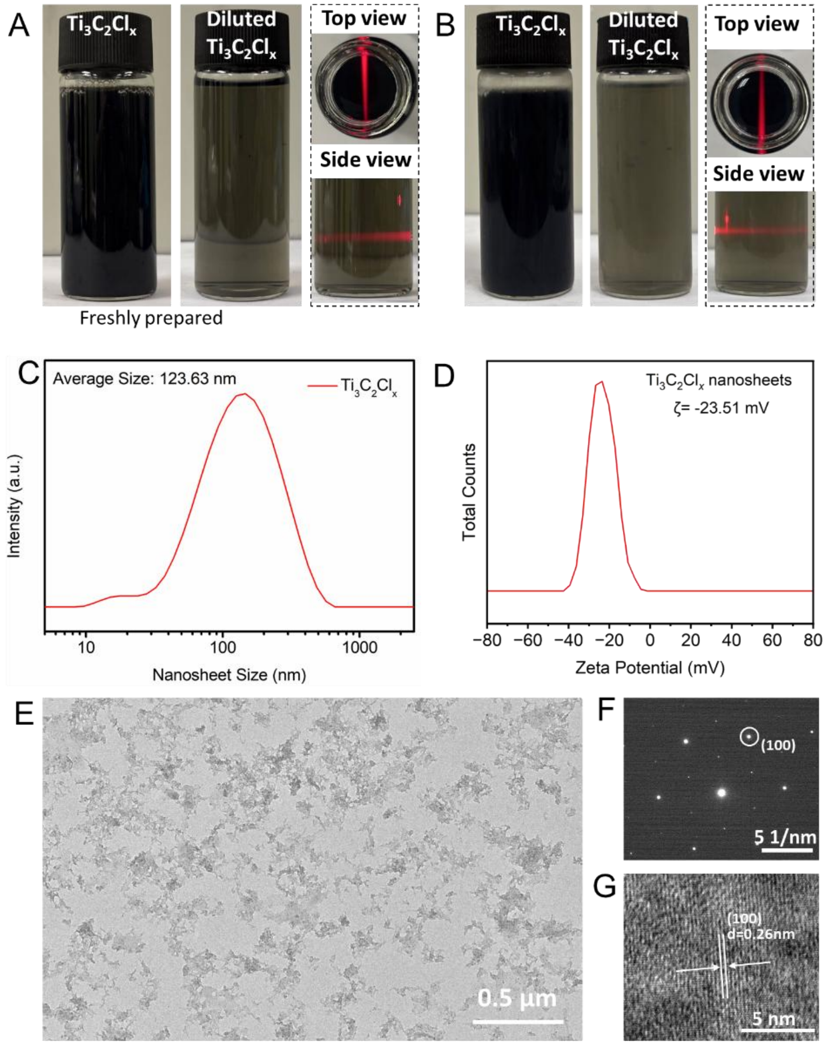

Fig. S19.

Characterization of $Ti_3C_2Cl_x$ nanosheet. Optical photographs of $Ti_3C_2Cl_x$ nanosheet dispersion solution: **(A)** freshly prepared, **(B)** after 7 days, showing the good dispersion and stability in water. **(C)** Dynamic light scattering (DLS) size distribution of $Ti_3C_2Cl_x$ nanosheets. **(D)** Zeta potential of $Ti_3C_2Cl_x$ nanosheets in deionized water. **(E)** TEM image of $Ti_3C_2Cl_x$ nanosheets, showing a uniform distribution. **(F)** Selected area electron diffraction of $Ti_3C_2Cl_x$ nanosheets, showing the hexagonal crystal structure. **(G)** High-resolution TEM image of $Ti_3C_2Cl_x$ nanosheets, showing the (100) crystal plane.

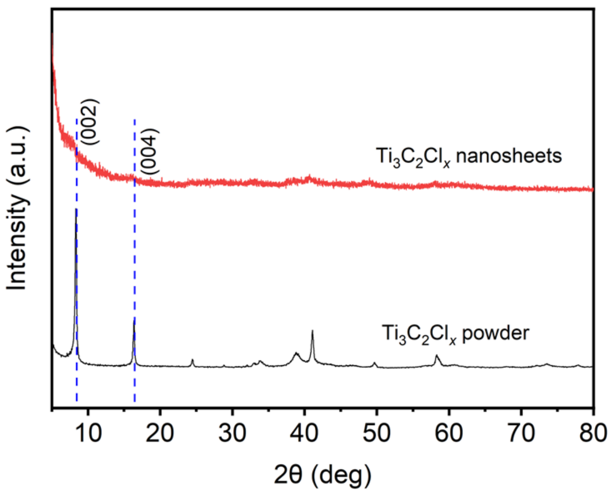

Fig. S20.
XRD patterns of $Ti_3C_2Cl_x$ nanosheets and particles.

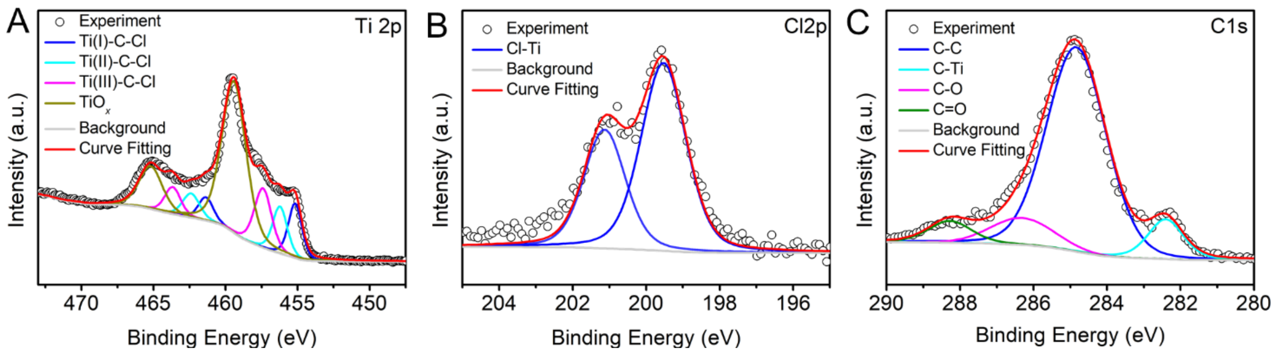

Fig. S21.

XPS analysis of $Ti_3C_2Cl_x$ nanosheets. XPS fitting results of Ti 2p **(A)**, Cl 2p **(B)**, and C 1s **(C)**.

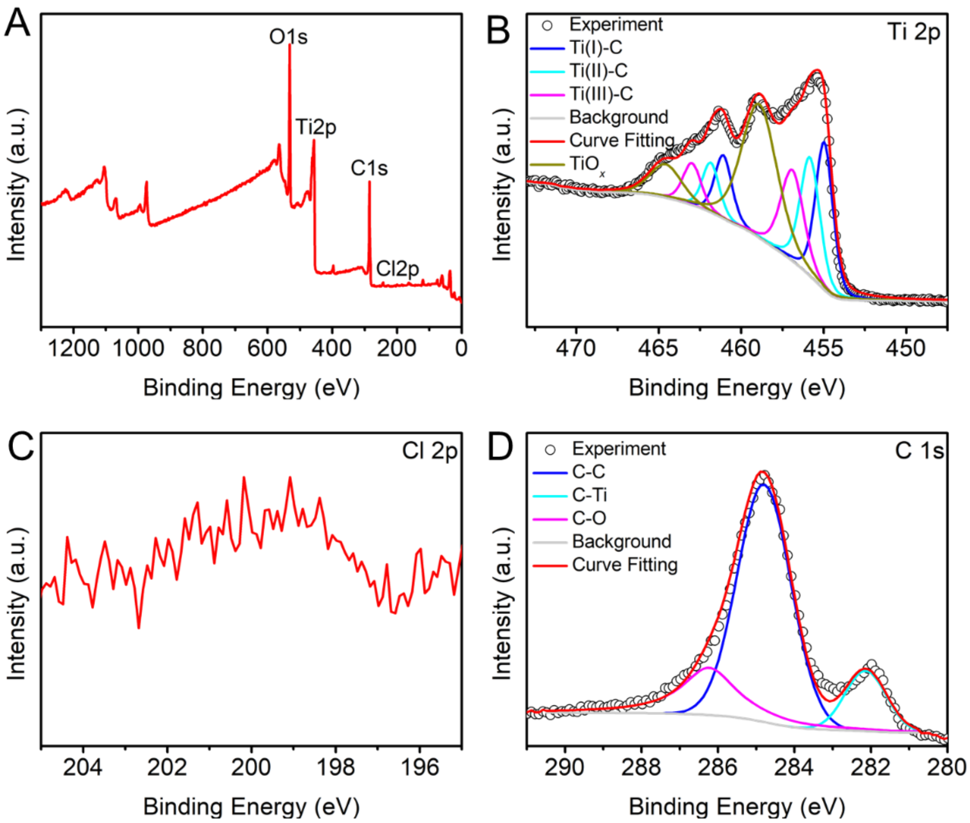

Fig. S22.

XPS analysis of reduced Ti_3C_2 nanosheets (1:8, 550°C) prepared in ascorbic acid solution. **(A)** The overview spectrum of reduced Ti_3C_2 nanosheets (1:8, 550°C) prepared in ascorbic acid solution, showing the signals of Ti 2p, Cl 2p, C 1s, and O 1s. XPS fitting results of Ti 2p **(B)**, Cl 2p **(C)**, and C 1s **(D)**.

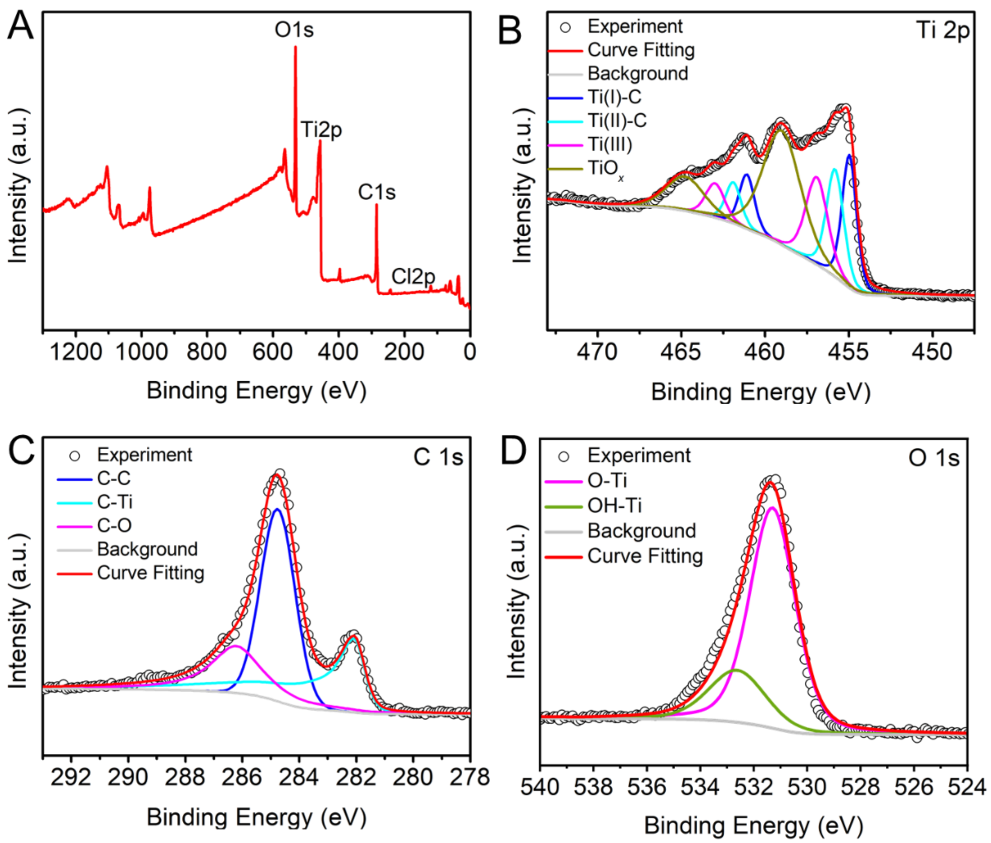

Fig. S23.

XPS analysis of reduced Ti_3C_2 nanosheets (1:8, 550°C) stored in deionized water for 24h. (A) The overview spectrum of reduced Ti_3C_2 nanosheets (1:8, 550°C) treated in deionized water for 24h, showing the signals of Ti 2p, C 1s, and O 1s. XPS fitting results of Ti 2p **(B)**, C 1*s* **(C)**, and O 1s **(D)**.

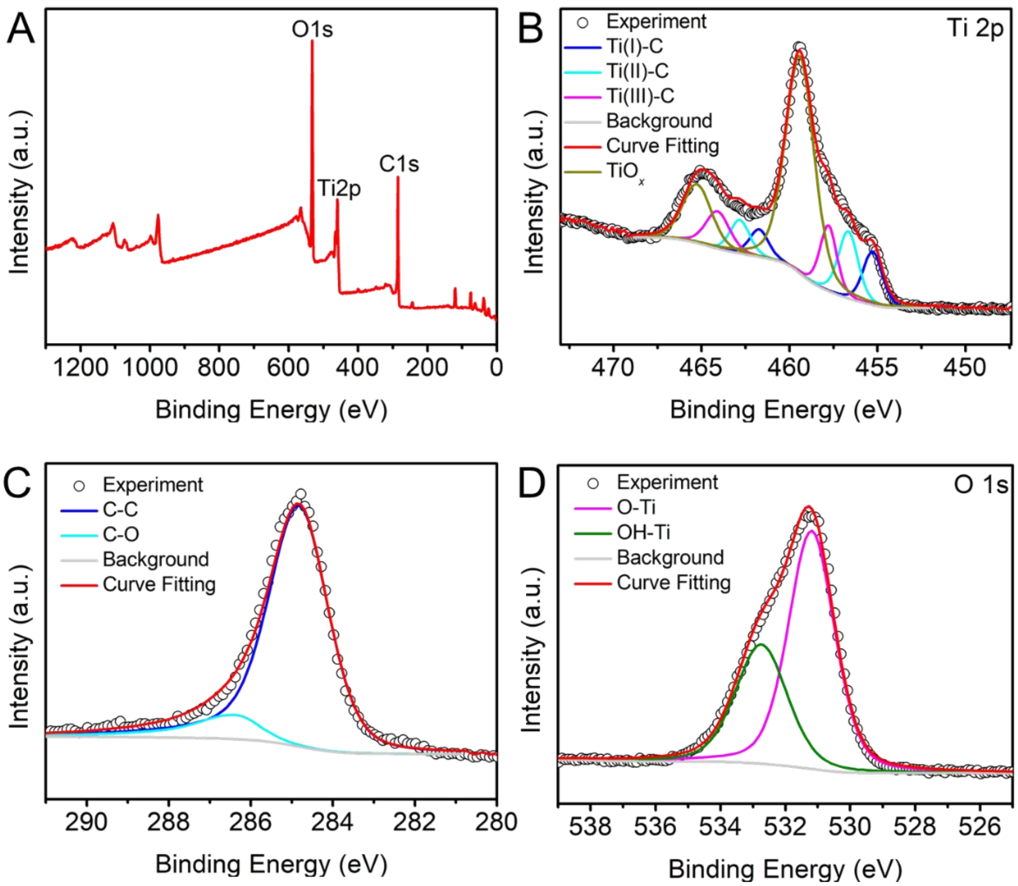

Fig. S24.

XPS analysis of reduced Ti_3C_2 nanosheets (1:8, 550°C) prepared without ascorbic acid solution. (A) The overview spectrum of reduced Ti_3C_2 nanosheets (1:8, 550°C) prepared without ascorbic acid solution, showing the signals of Ti 2p, C 1s, and O 1s. XPS fitting results of Ti 2p **(B)**, C 1s **(C)**, and O 1s **(D)**.

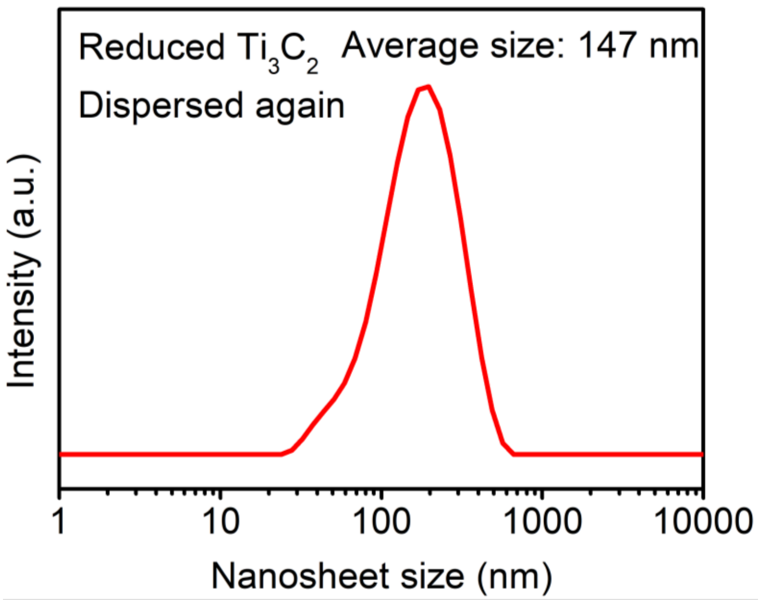

Fig. S25.
Size distribution of reduced Ti_3C_2 (1:8, 550°C) nanosheets dispersed again in deionized water after vacuum filtration.

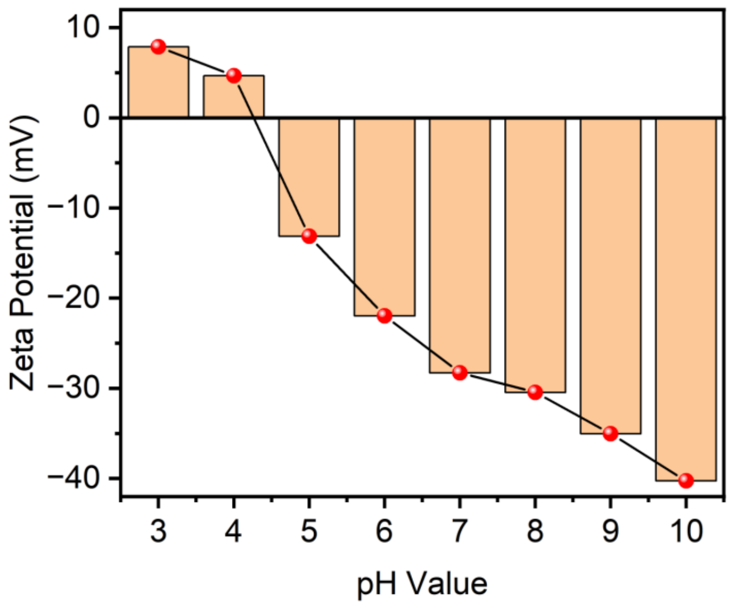

Fig. S26.

Zeta potentials of $Ti_3C_2T_x$ nanosheets obtained from the traditional HF method with the change of pH values.

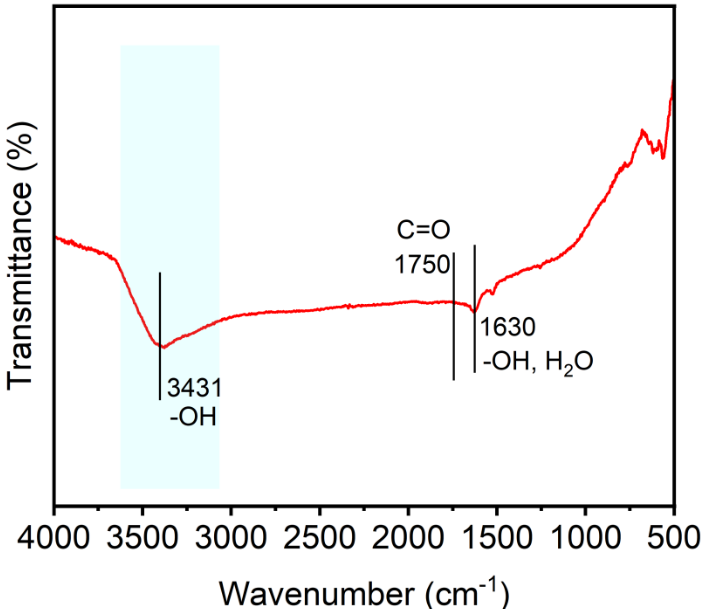

Fig. S27.
FTIR spectrum of reduced Ti_3C_2 nanosheets, confirming the absence of ascorbic acid residue, as no characteristic absorption band of the C=O bond.

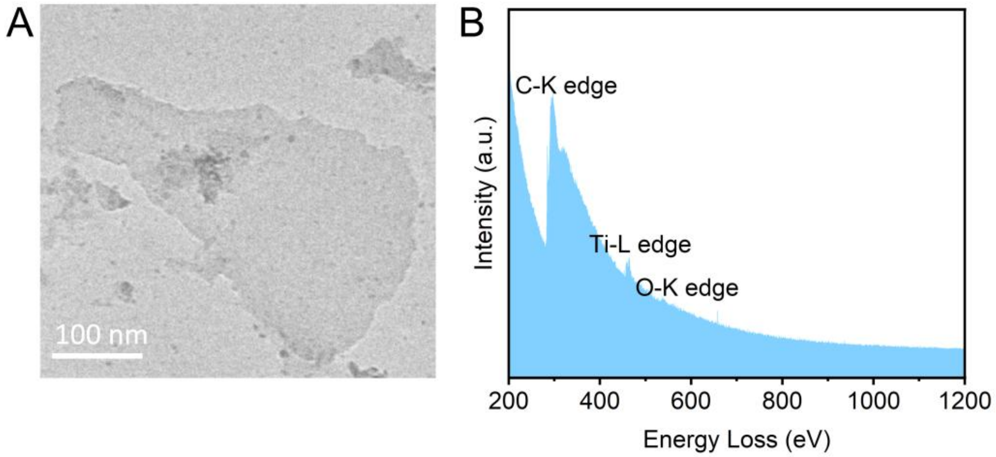

Fig. S28.

STEM characterization of reduced Ti_3C_2 (1:8, 550°C) nanosheets. TEM image **(A)** and its corresponding energy loss (EELS) spectrum **(B)** of reduced Ti_3C_2 (1:8, 550°C) nanosheets

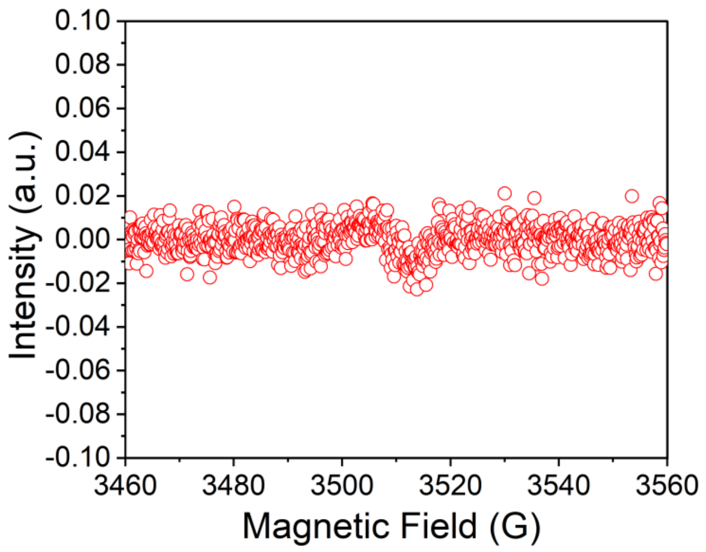

Fig. S29.
EPR spectrum of reduced Ti_3C_2 (1:8, 550°C) nanosheets.

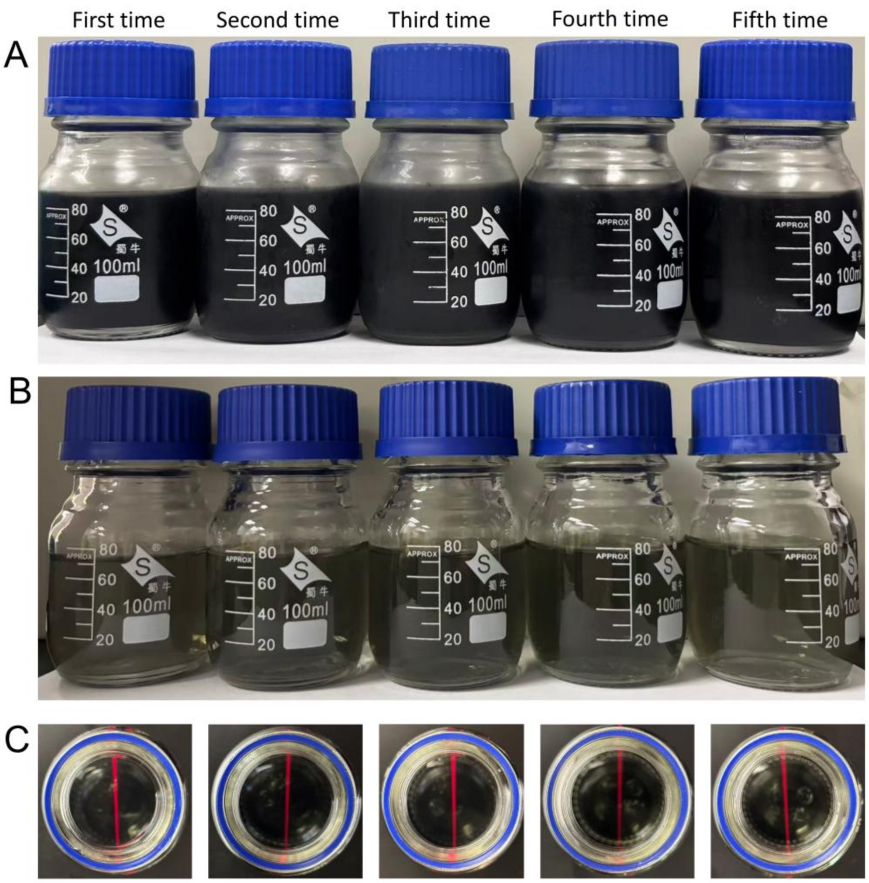

Fig. S30.
Photographs of reduced Ti_3C_2 (1:8, 550°C) nanosheet suspensions after 1-5 ultrasonic exfoliation cycles (0.5 g starting powder). (A) Concentrated suspensions. **(B)** Diluted counterparts of (A). **(C)** The Tyndall effect was observed in the diluted suspensions, verifying their colloidal stability.

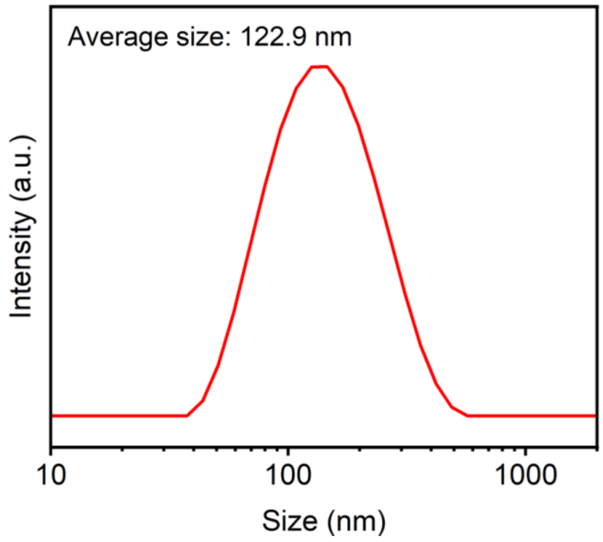

Fig. S31.
DLS size distribution of the as-obtained reduced Ti_3C_2 (1:8, 550°C) nanosheets obtained after five cycles of ultrasonic exfoliation, showing an average size of 122.9 nm.

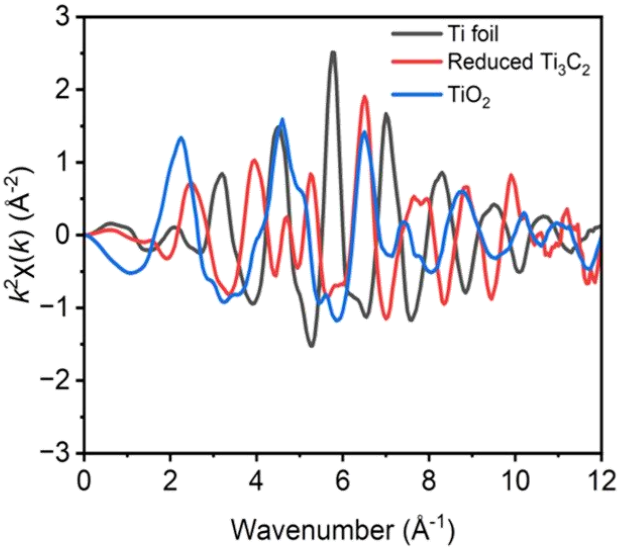

Fig. S32.

Normalized Ti *K*-edge EXAFS oscillations (k^2-weighted $\chi(k)$ functions) of Ti foil, reduced Ti_3C_2 (1:8, 550°C) nanosheets, and TiO_2 in *k*-space, presenting the oscillatory features that reflect the structural variations.

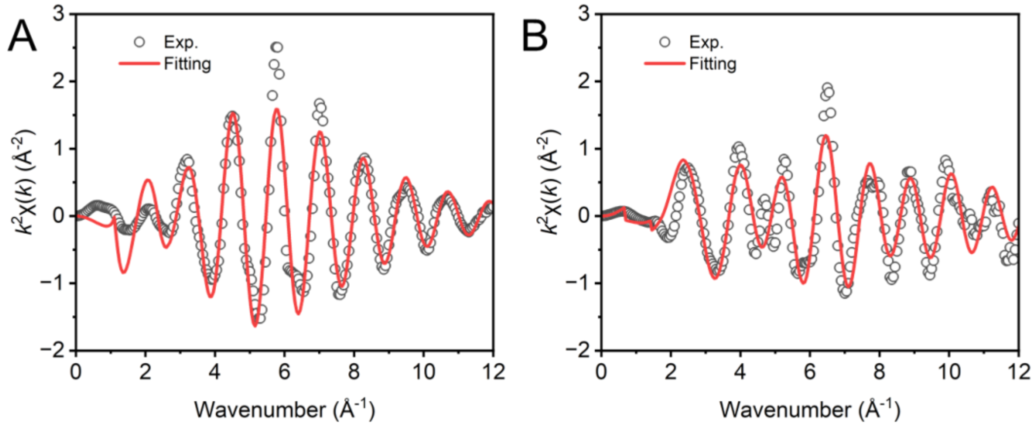

Fig. S33.

EXAFS fitting of Ti foil and reduced Ti_3C_2 (1:8, 550°C) nanosheets in *k*-space. (A) k^2-weighted EXAFS oscillation and fitting curves for Ti foil in *k*-space. **(B)** k^2-weighted EXAFS oscillation and fitting curves for reduced Ti_3C_2 (1:8, 550°C) nanosheets in k-space.

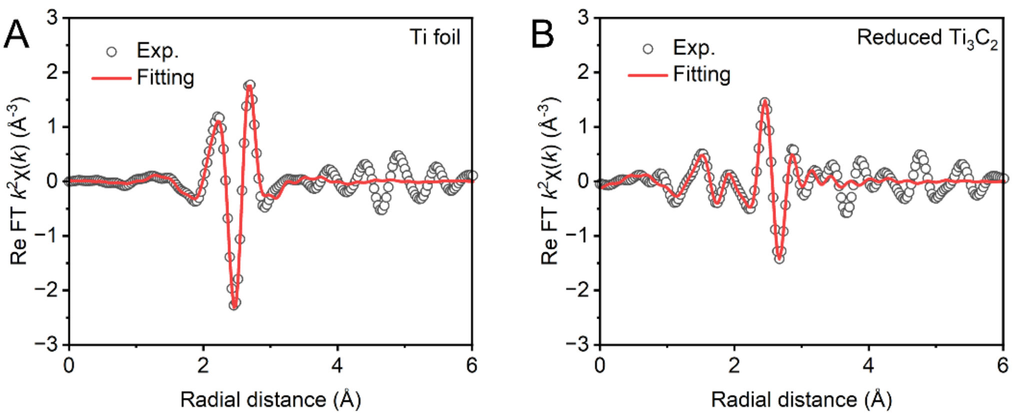

Fig. S34.

Real part fitting of Fourier-transformed extended X-ray absorption fine structure (FT-EXAFS) spectra in *R*-space. (A) Real part of FT-EXAFS and fitting curves for Ti foil in *R*-space. Open circles are experimental data, and the red line is the fitting result. **(B)** Real part of FT-EXAFS and fitting curves for reduced Ti_3C_2 (1:8, 550°C) nanosheets in *R*-space.

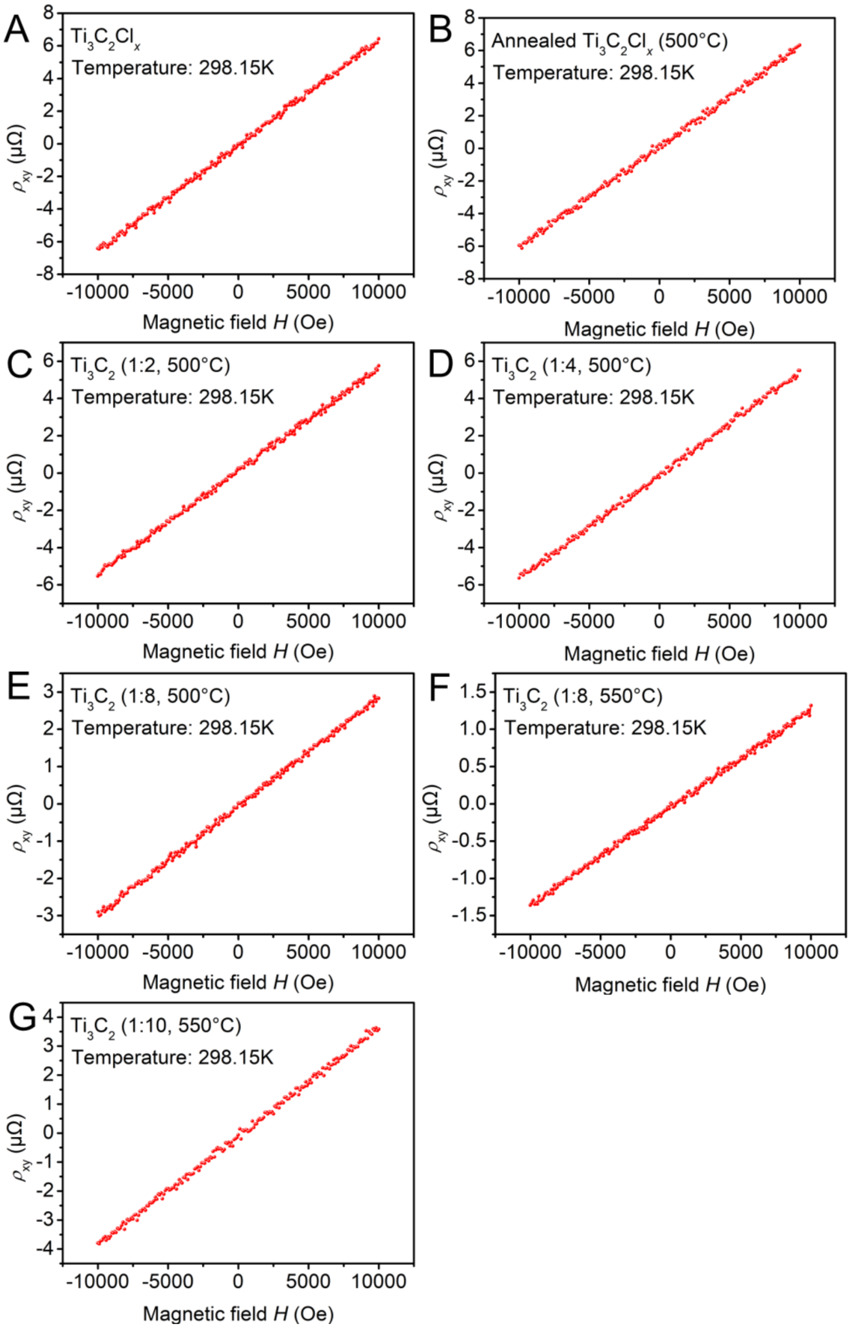

Fig. S35.

Hall effect results of different MXenes. (A) Pristine $Ti_3C_2Cl_x$. **(B)** Annealed $Ti_3C_2Cl_x$ (500°C). **(C)** Reduced Ti_3C_2 (1:2, 500°C). **(D)** Reduced Ti_3C_2 (1:4, 500°C). **(E)** reduced Ti_3C_2 (1:8, 500°C). **(F)** Reduced Ti_3C_2 (1:8, 550°C). **(G)** Reduced Ti_3C_2 (1:10, 550°C)

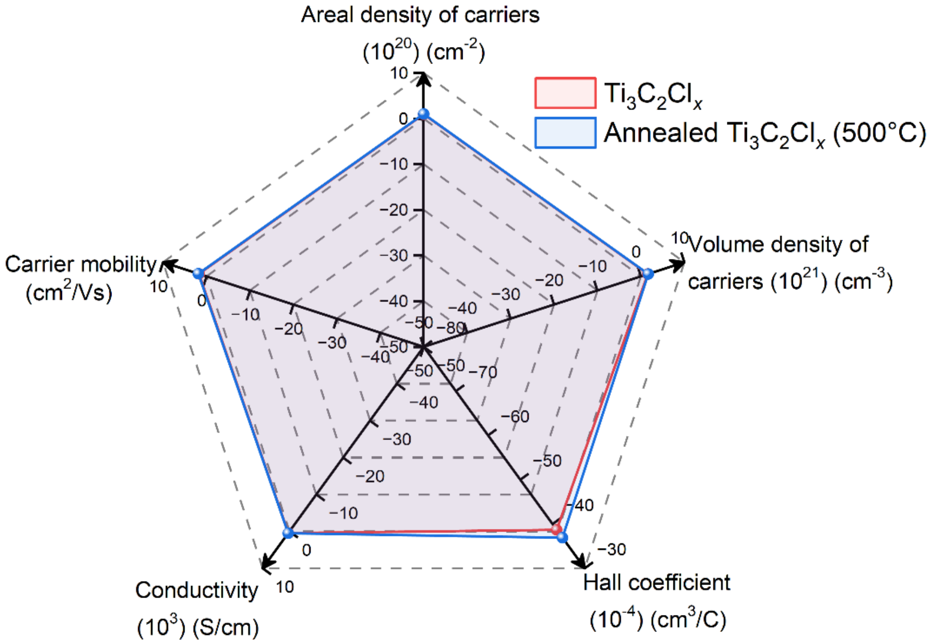

Fig. S36.

Radar map of the electronic information of $Ti_3C_2Cl_x$ and annealed $Ti_3C_2Cl_x$ (500°C) based on the test of the Hall effect measurement, showing the electron concentration, mobility, conductivity, and Hall coefficient.

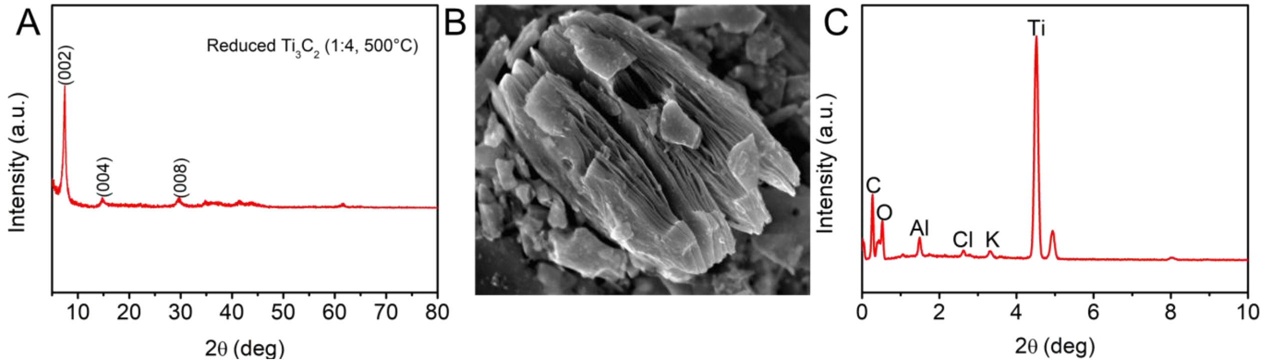

Fig. S37.
Characterization of reduced Ti_3C_2 (1:4, 500°C). (A) XRD pattern of reduced Ti_3C_2 (1:4, 500°C). **(B)** SEM image of reduced Ti_3C_2 (1:4, 500°C). **(C)** SEM-EDS spectrum of reduced Ti_3C_2 (1:4, 500°C).

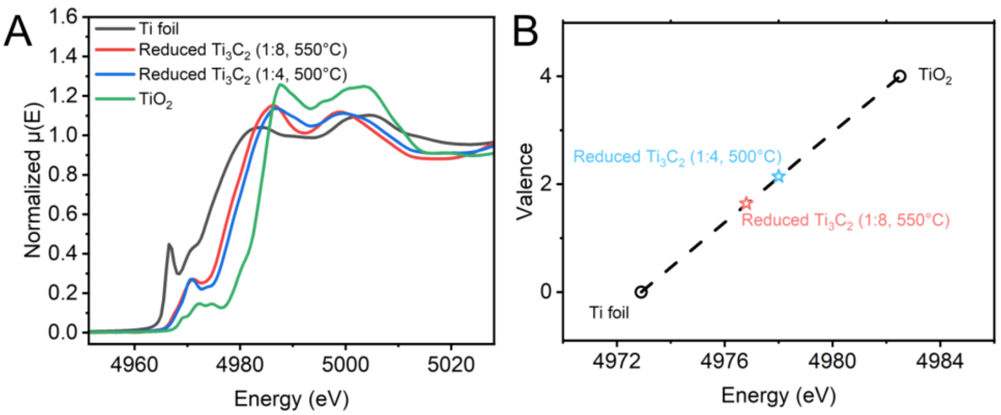

Fig. S38.

X-ray absorption near-edge structure (XANES) characterization of reduced Ti_3C_2 MXenes. **(A)** Ti *K*-edge XANES spectra of Ti foil, reduced Ti_3C_2 (1:8, 550°C) nanosheets, reduced Ti_3C_2 (1:4, 500°C), and TiO_2. **(B)** Valence state plot of Ti species, with Ti foil, reduced Ti_3C_2 (1:8, 550°C) nanosheets, reduced Ti_3C_2 (1:4, 500°C), and TiO_2 positioned to show the variation in Ti valence.

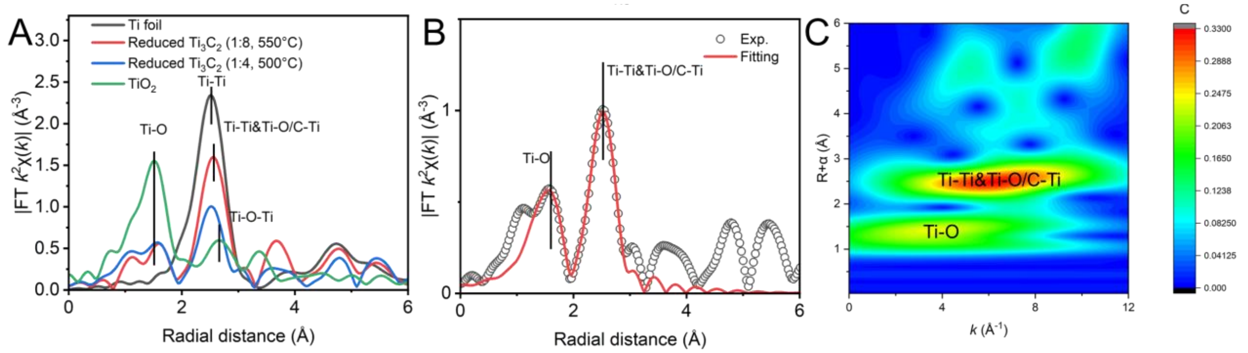

Fig. S39.

EXAFS characterization of reduced Ti_3C_2 MXenes in *R*-space. (A) FT-EXAFS spectra of Ti foil, reduced Ti_3C_2 (1:8, 550°C) nanosheets, reduced Ti_3C_2 (1:4, 500°C), and TiO_2 in *R*-space. **(B)** FT-EXAFS and fitting curves for reduced Ti_3C_2 (1:4, 500°C) in *R*-space. **(C)** The wavelet transform analysis in Ti *K*-edge for reduced Ti_3C_2 (1:4, 500°C).

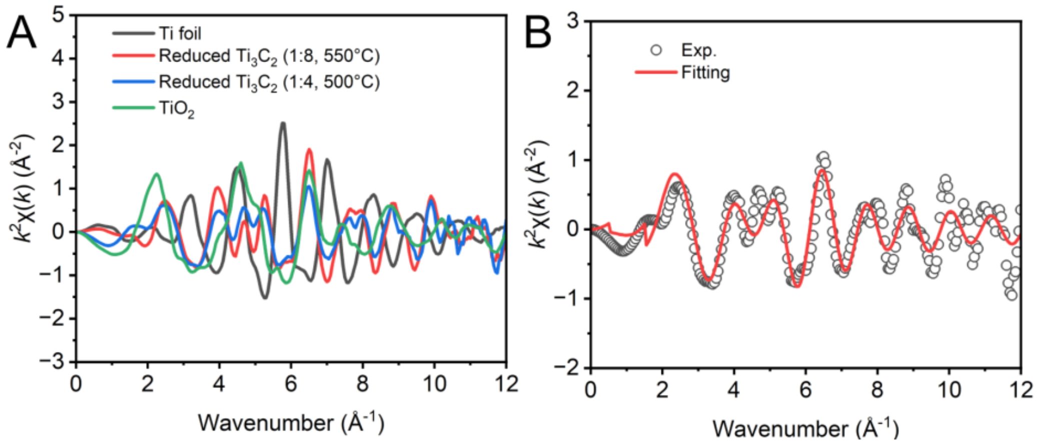

Fig. S40.

Ti *K*-edge EXAFS characterization of reduced Ti_3C_2 MXenes. (A) Normalized Ti *K*-edge EXAFS oscillations (k^2-weighted $\chi(k)$ functions) of Ti foil, reduced Ti_3C_2 (1:8, 550°C) nanosheets, reduced Ti_3C_2 (1:4, 500°C), and TiO_2 in *k*-space. **(B)** k^2-weighted EXAFS oscillation and fitting curves for reduced Ti_3C_2 (1:4, 500°C) in *k*-space.

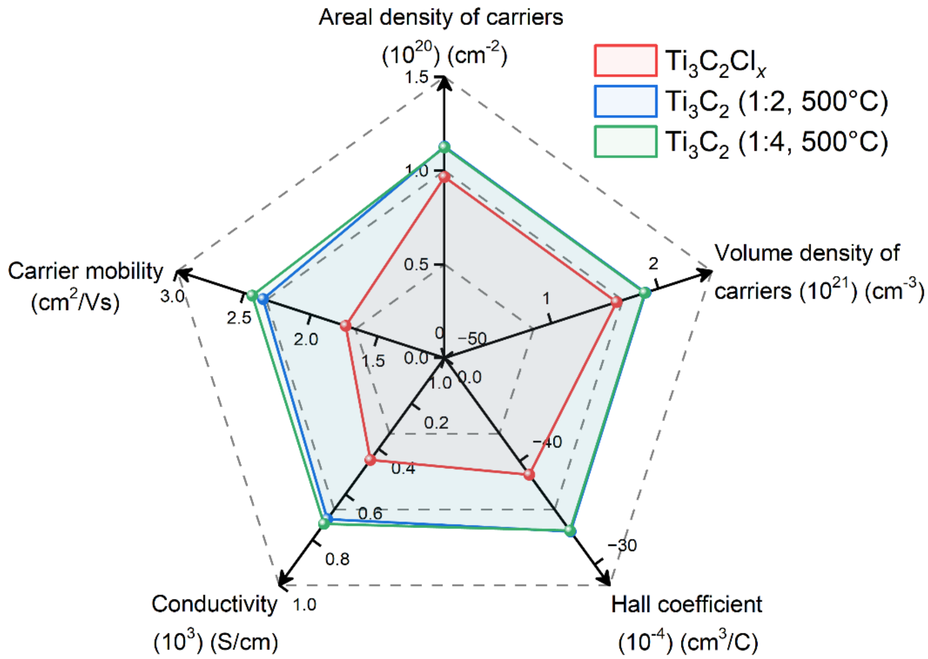

Fig. S41.
Radar map of the electronic information of $Ti_3C_2Cl_x$, reduced Ti_3C_2 (1:2, 500°C), and reduced Ti_3C_2 (1:4, 500°C) based on the test of the Hall effect measurement.

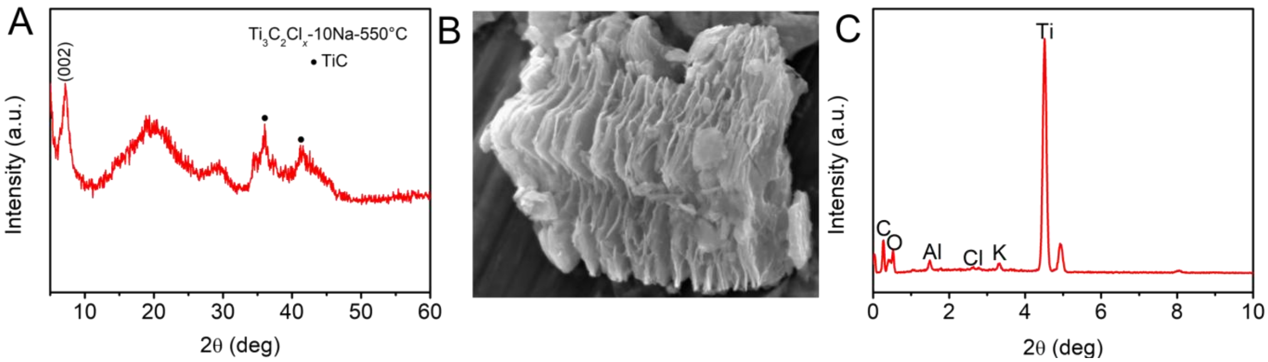

Fig. S42.

Characterization of reduced Ti_3C_2 (1:10, 550°C). (A) XRD pattern of reduced Ti_3C_2 (1:10, 550°C). **(B)** SEM image of reduced Ti_3C_2 (1:10, 550°C). **(C)** SEM-EDS spectrum of reduced Ti_3C_2 (1:10, 550°C).

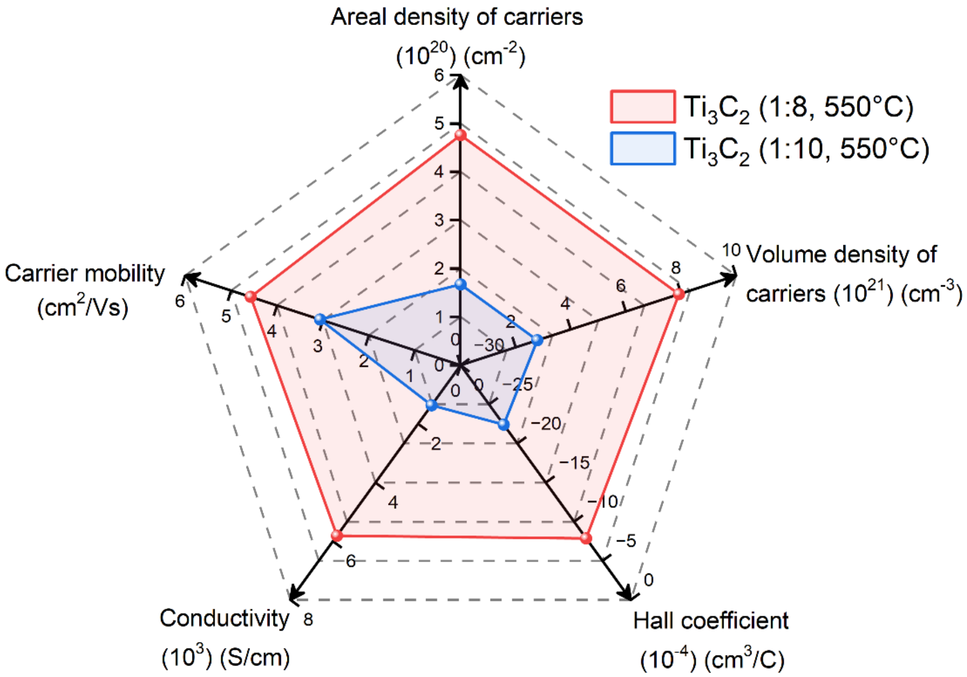

Fig. S43.

Radar map of the electronic information of reduced Ti_3C_2 (1:8, 550℃) and reduced Ti_3C_2 (1:10, 550℃) based on the test of the Hall effect measurement.

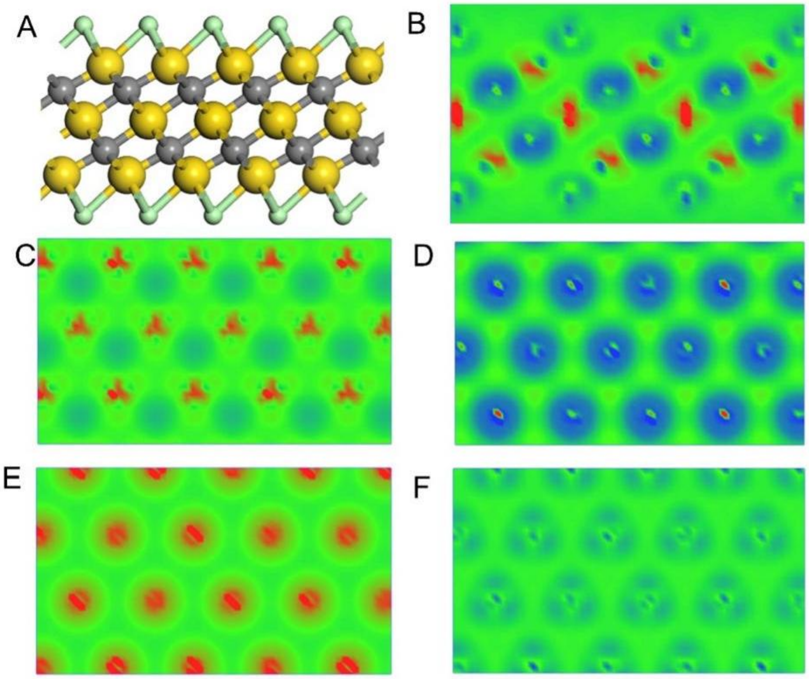

Fig. S44.

Two-dimensional charge density of $Ti_3C_2Cl_x$. (A) Schematic illustration of the atomic structure of $Ti_3C_2Cl_x$. **(B)** Charge density of $Ti_3C_2Cl_x$ observed from the side view. **(C)** In-plane charge density of outer Ti atoms. **(D)** In-plane charge density of outer C atoms. **(E)** In-plane charge density of inner Ti atoms. **(F)** In-plane charge density of Cl atoms. Red represents positive charge, blue represents the negative charge, and green represents neutral charge.

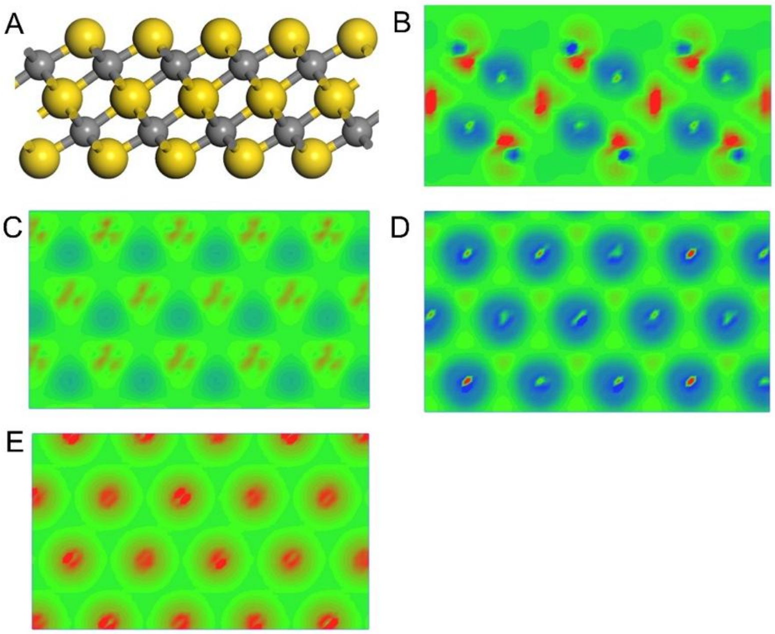

Fig. S45.

Two-dimensional charge density of Ti_3C_2 after removing -Cl terminations. (A) Schematic illustration of the atomic structure of Ti_3C_2 after optimizing the surface coordination. **(B)** Charge density of Ti_3C_2 after removing -Cl terminations observed from the side view. **(C)** In-plane charge density of outer Ti atoms. **(D)** In-plane charge density of outer C atoms. **(E)** In-plane charge density of inner Ti atoms. Red represents positive charge, blue represents the negative charge, and green represents neutral charge.

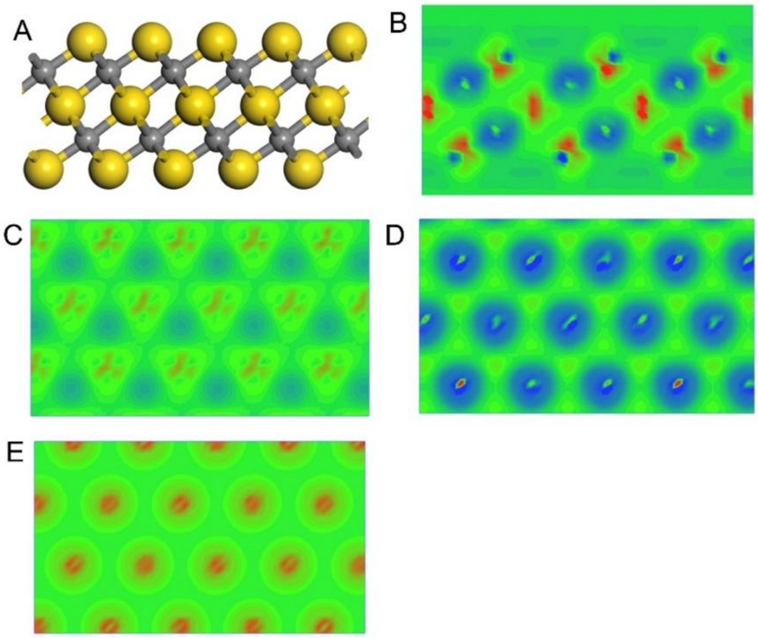

Fig. S46.
Two-dimensional charge density of Ti_3C_2 after injecting electrons. (A) Schematic illustration of the atomic structure of Ti_3C_2 after injecting electrons into the lattice, simulating the status of reduced Ti_3C_2. **(B)** Charge density of Ti_3C_2 observed from the side view. **(C)** In-plane charge density of outer Ti atoms. **(D)** In-plane charge density of outer C atoms. **(E)** In-plane charge density of inner Ti atoms. Red represents positive charge, blue represents the negative charge, and green represents neutral charge.

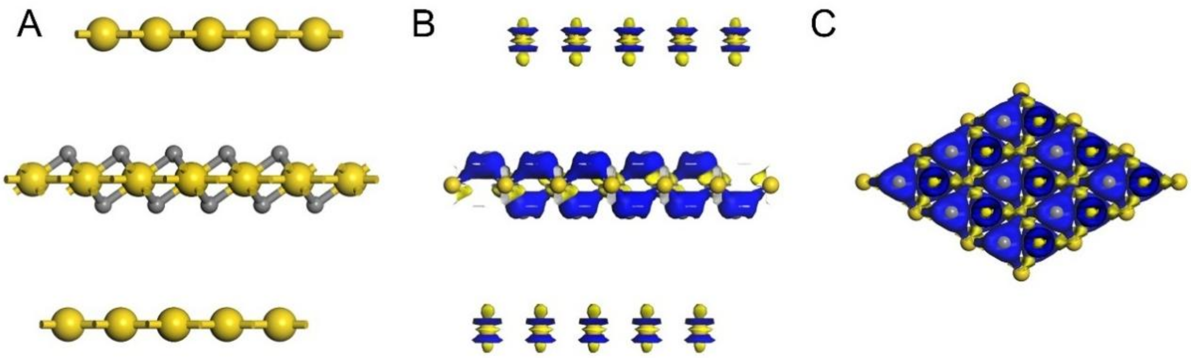

Fig. S47.

Structure change and three-dimensional charge density of Ti_3C_2 after injecting excessive electrons. (A) Schematic illustration of the atomic structure of degraded Ti_3C_2 after excessively injecting electrons into the lattice. Charge density of the degraded Ti_3C_2 observed from side view **(B)** and top view **(C)**.

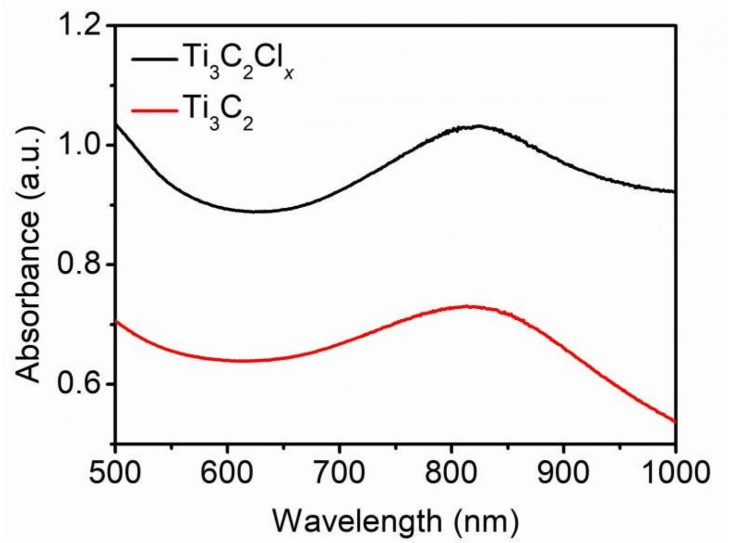

Fig. S48.
UV-Vis absorbance spectra of $Ti_3C_2Cl_x$ nanosheets and reduced Ti_3C_2 (1:8, 550°C) nanosheets.

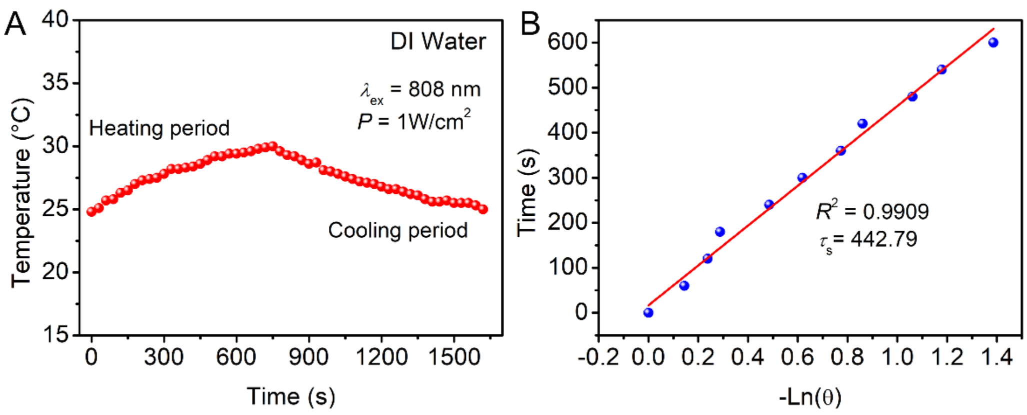

Fig. S49.

Photothermal characterization of deionized water. (A) Temperature curve of deionized water, including two periods: temperature rise during laser irradiation and temperature drop after turning off the laser. **(B)** Fitted time constant (τ_s) for heat dissipation based on the data of temperature drop.

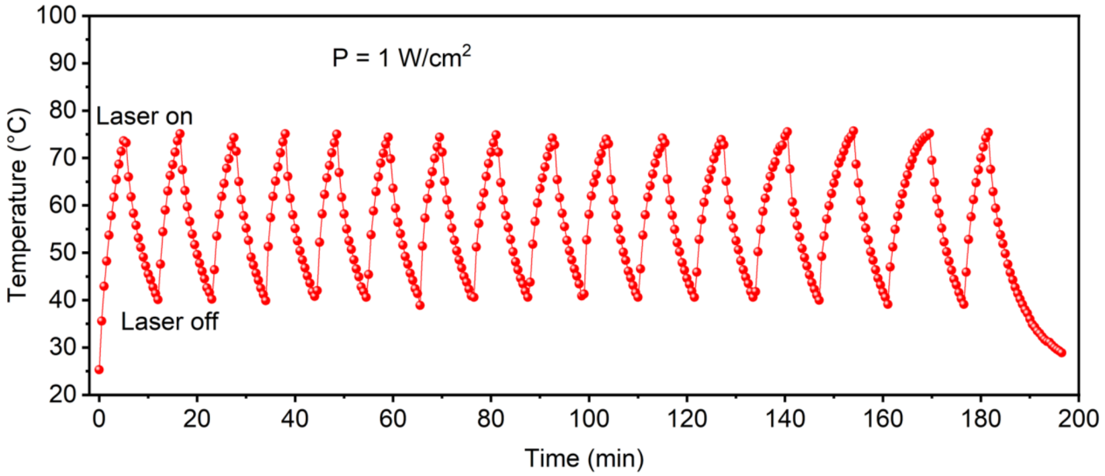

Fig. S50.
Stability test over 15 on/off laser cycles (1 W/cm^2) for the solution of reduced Ti_3C_2 (1:8, 550°C) nanosheets with a 50 μg/mL concentration.

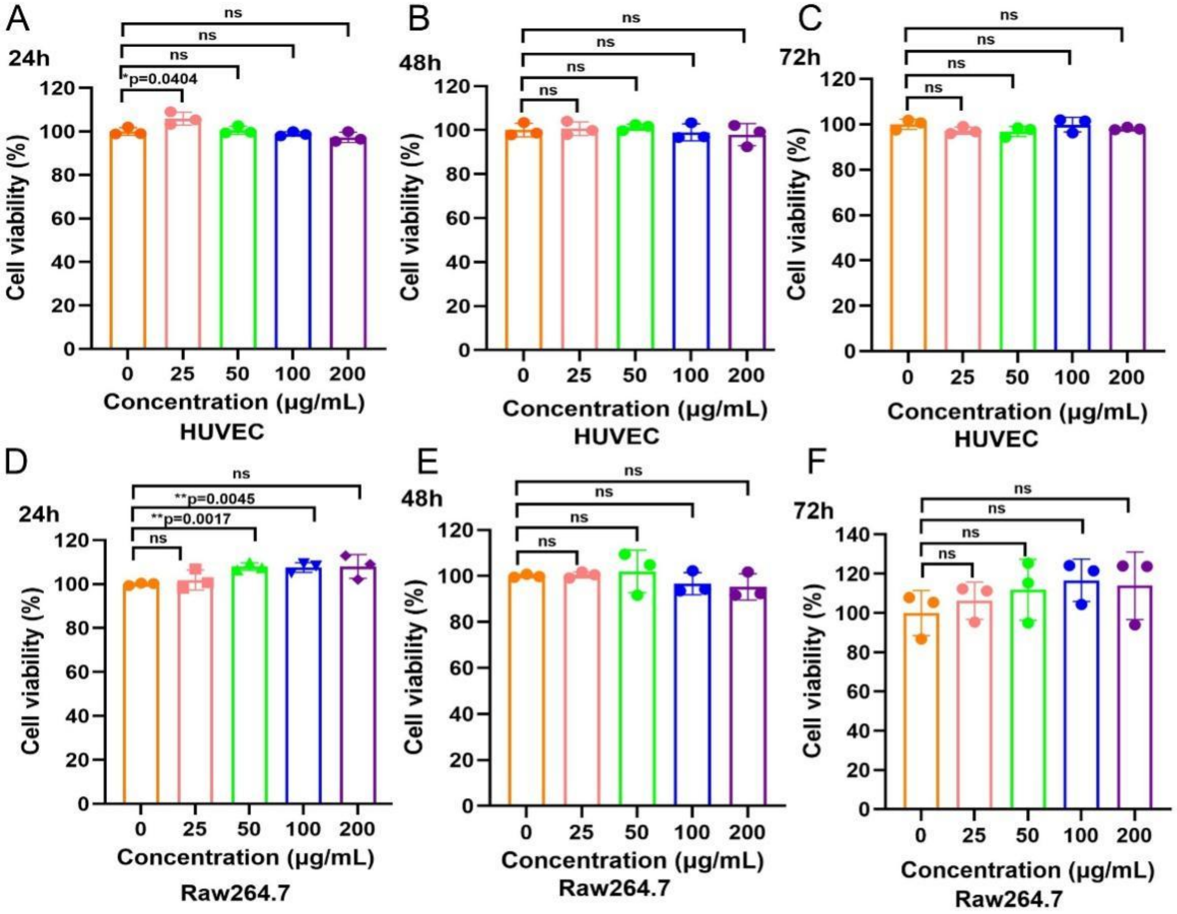

Fig. S51.

Cell toxicity test of reduced MXene. HUVEC cell toxicity results of reduced MXene for 24 h **(A)**, 48 h **(B)**, and 72 h **(C)**. Raw264.7 cell toxicity results of reduced MXene for 24 h **(D)**, 48 h **(E)**, and 72 h **(F)**.

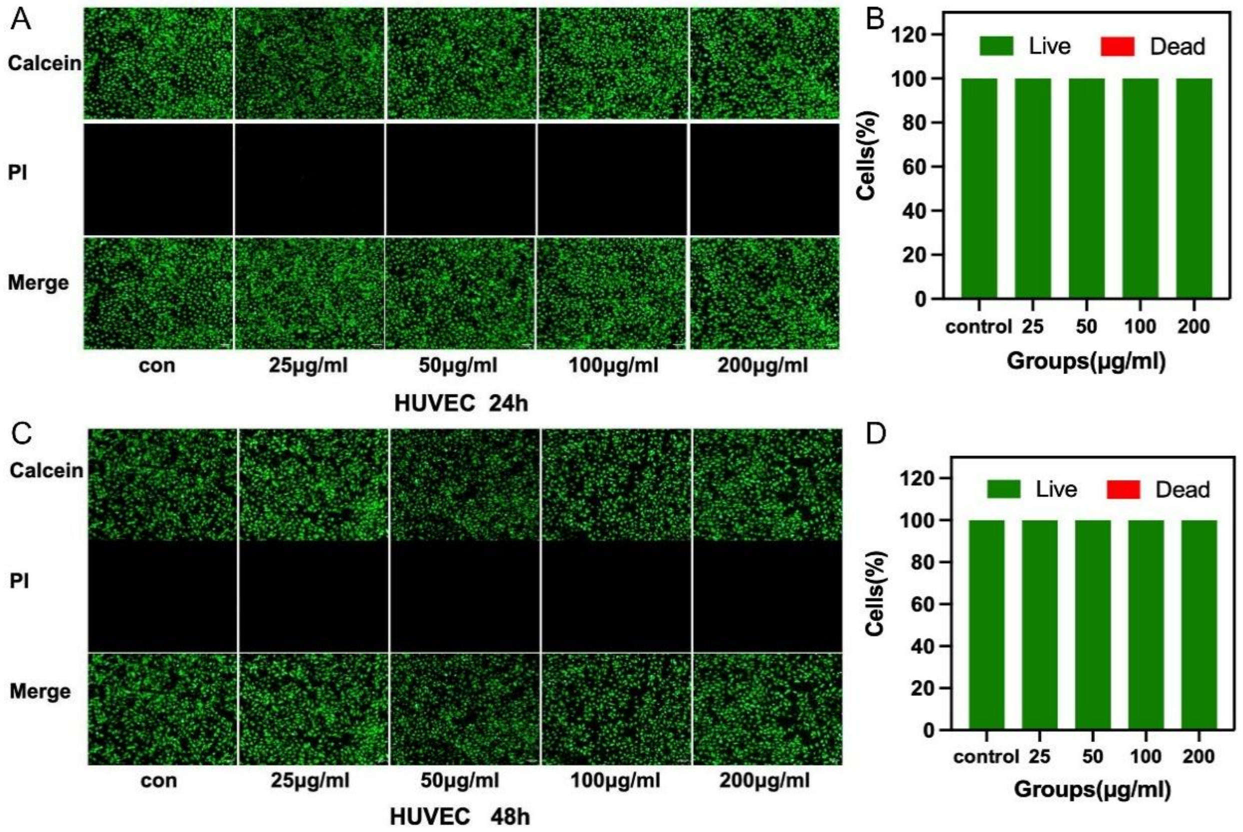

Fig. S52.

Cell viability staining after co-culture with reduced MXene at different concentrations. (A) Immunofluorescence image of HUVEC cells for 24 h, green: live cells, red: dead cells. **(B)** Statistical analysis of HUVEC cells for 24 h. **(C)** Immunofluorescence image of HUVEC cells for 48 h, green: live cells, red: dead cells. **(D)** Statistical analysis of HUVEC cells for 48 h.

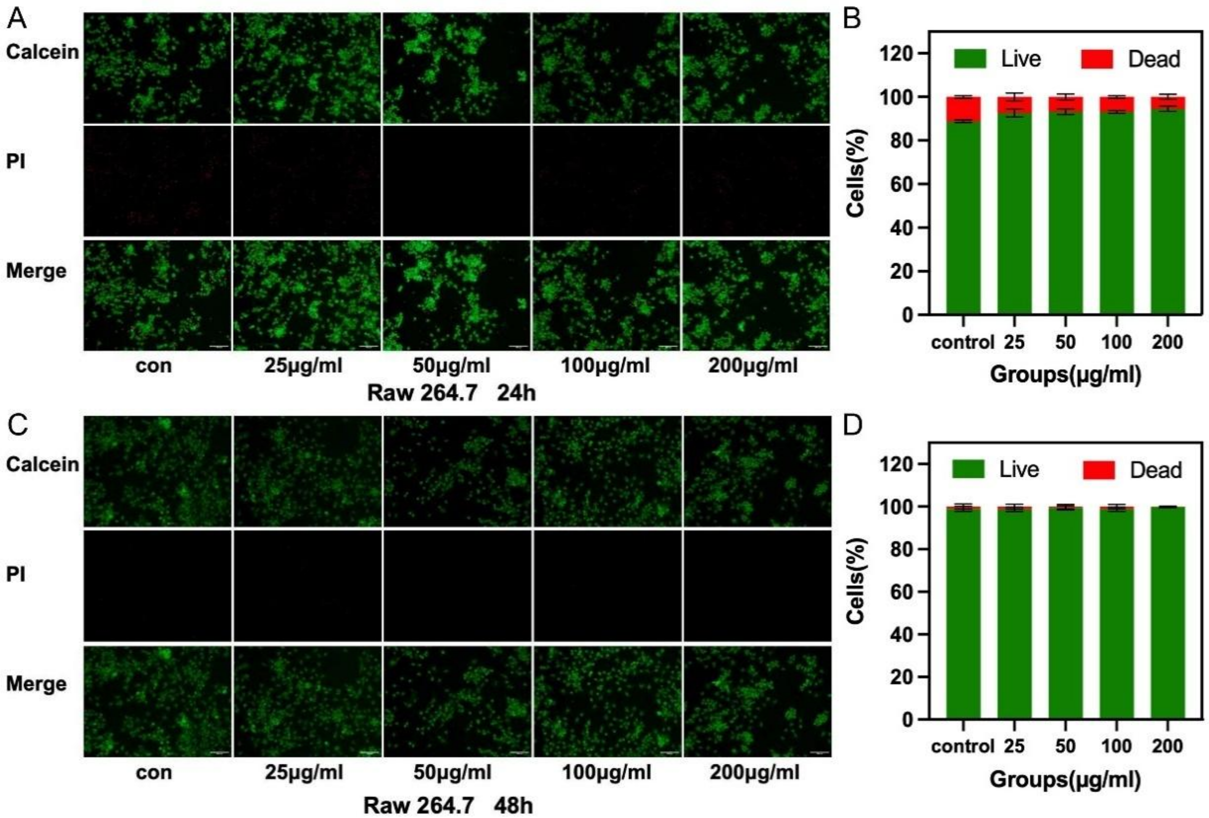

Fig. S53.

Cell viability staining after co-culture with reduced MXene at different concentrations. (A) Immunofluorescence image of Raw 264.7 cells for 24 h, green: live cells, red: dead cells. **(B)** Statistical analysis of HUVEC cells for 24 h. **(C)** Immunofluorescence image of Raw 264.7 cells for 48 h, green: live cells, red: dead cells. **(D)** Statistical analysis of HUVEC cells for 48 h.

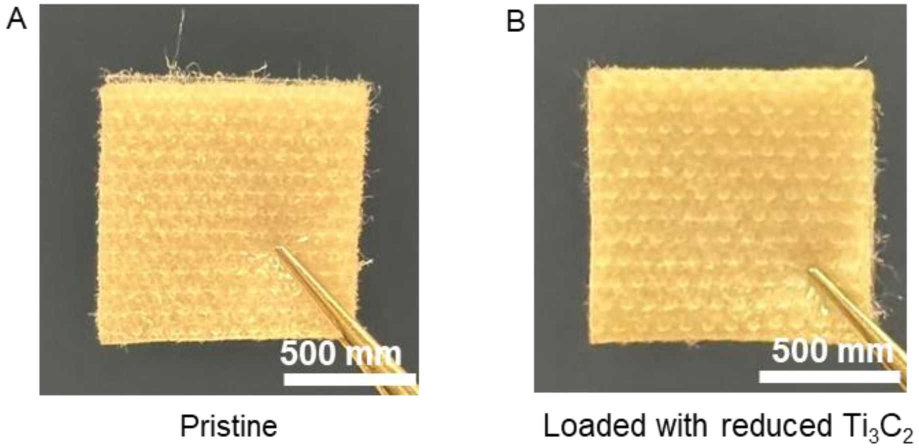

Fig. S54.
Optical photographs of woundplasts. **(A)** Pristine woundplast. **(B)** Woundplast loaded with reduced Ti_3C_2 (1:8, 550°C) nanosheets.

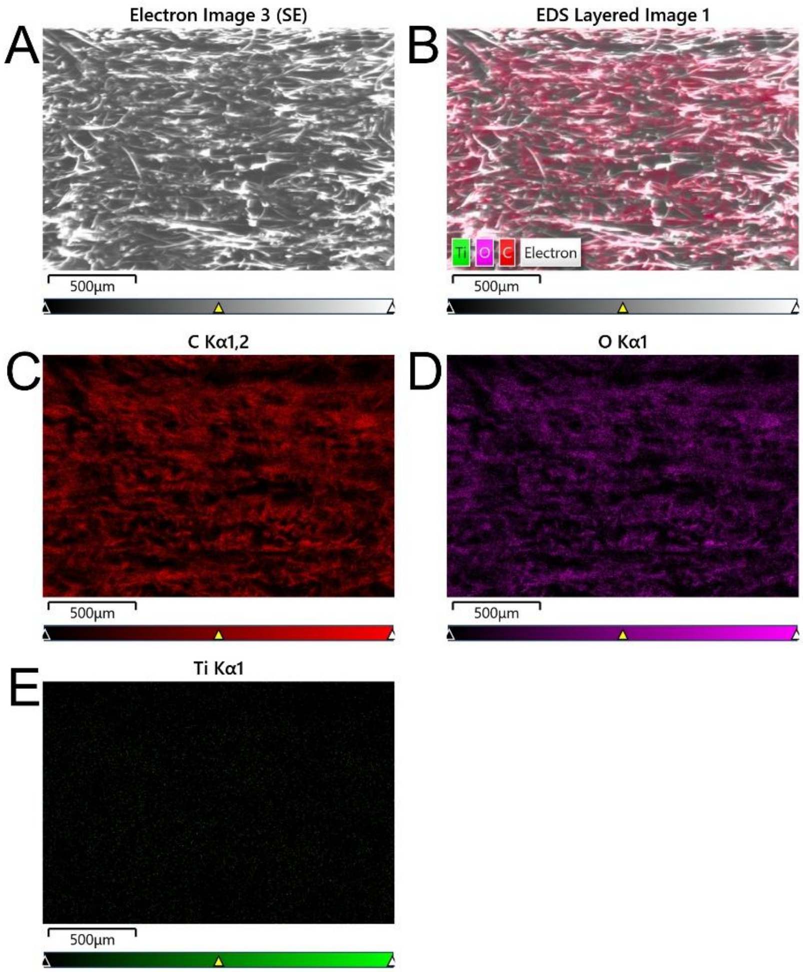

Fig. S55.

SEM characterization of woundplast loaded with reduced Ti_3C_2 (1:8, 550°C) nanosheets. (A) SEM image of woundplast loaded with reduced Ti_3C_2 (1:8, 550°C) nanosheets. **(B)** Overlaid EDS elemental map, with Ti (green), C (red), and O (pink) superimposed on the corresponding SEM image. **(C-E)** Individual EDS elemental maps for C, O, and Ti, respectively, showing the spatial distribution of each element across the same region shown in **(A)**.

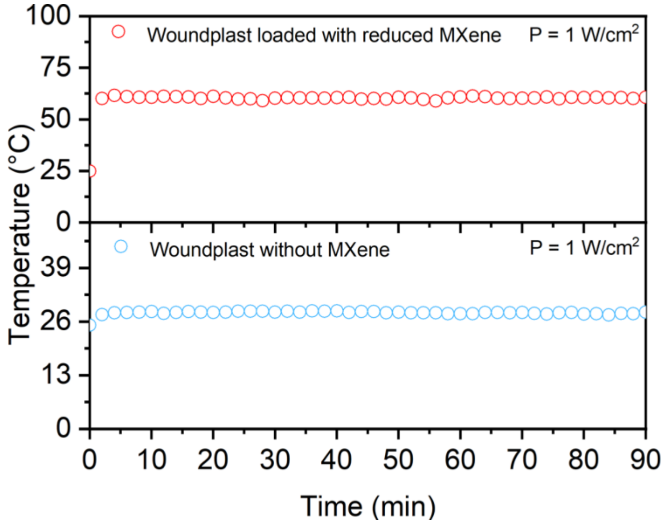

Fig. S56.

Stability test over 90 minutes irradiation of an 808 nm laser (1 W/cm^2) for woundplast loaded with reduced Ti_3C_2 (1:8, 550°C) nanosheets (2.8 μg/cm^2).

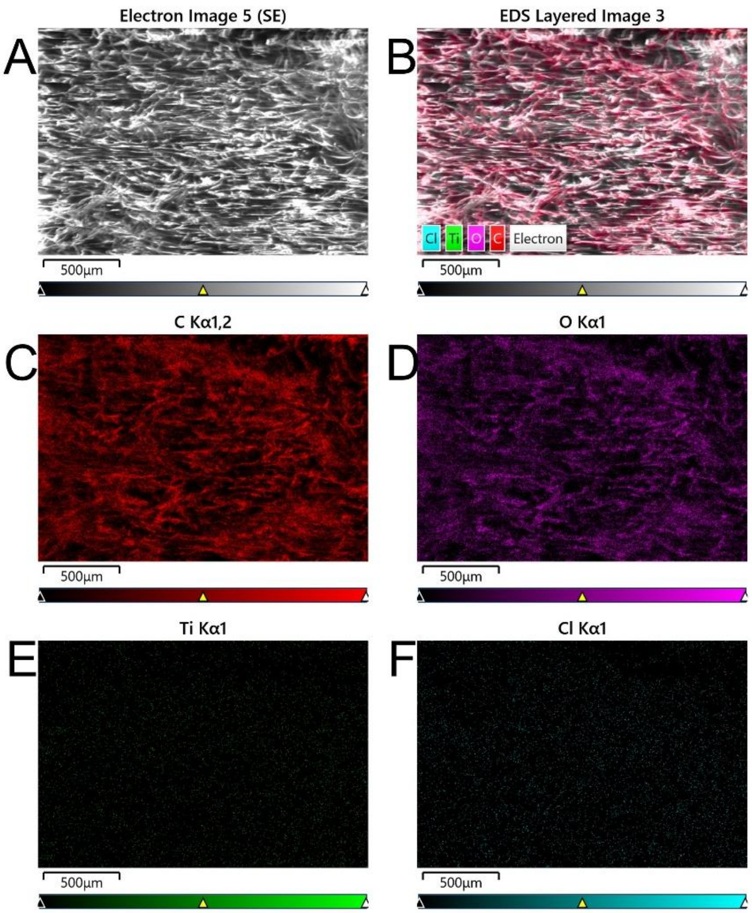

Fig. S57.

SEM characterization of woundplast loaded with $Ti_3C_2Cl_x$ nanosheets. (A) SEM image of woundplast loaded with $Ti_3C_2Cl_x$ nanosheets. **(B)** Overlaid EDS elemental map, with Ti (green), C (red), O (pink), and Cl (cyan) superimposed on the corresponding SEM image. **(C-F)** Individual EDS elemental maps for C, O, Ti, and Cl, respectively, showing the distribution of each element across the same region shown in **(A)**.

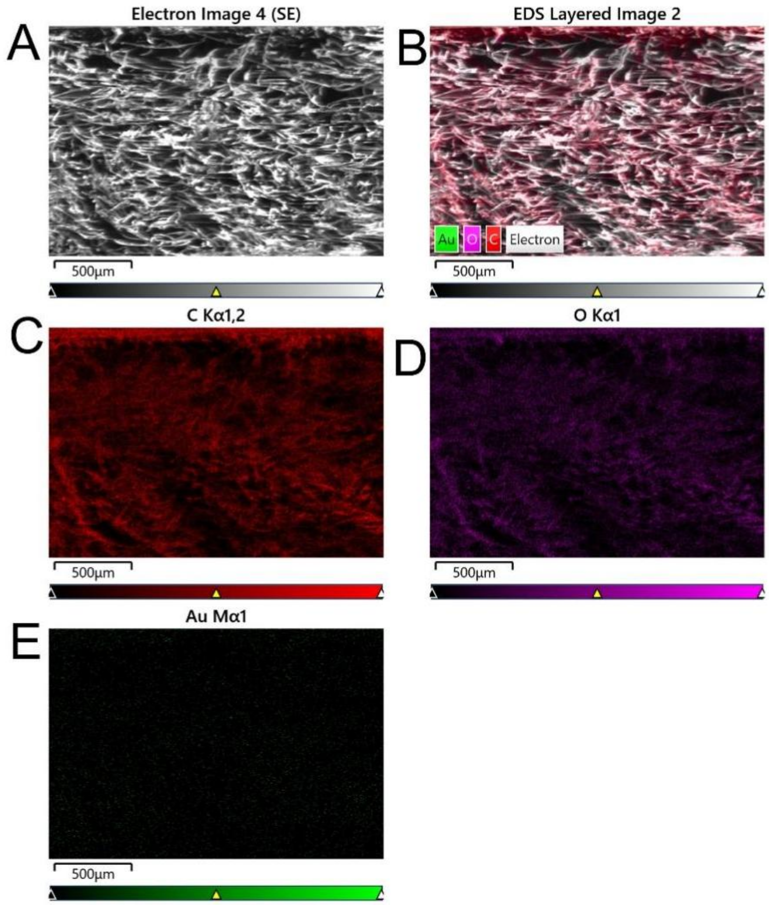

Fig. S58.

SEM characterization of woundplast loaded with Au nanoparticles. (A) SEM image of woundplast loaded with Au nanoparticles. **(B)** Overlaid EDS elemental map, with Au (green), C (red), and O (pink) superimposed on the corresponding SEM image. **(C-E)** Individual EDS elemental maps for C, O, and Au, respectively, showing the distribution of each element across the same region shown in **(A)**.

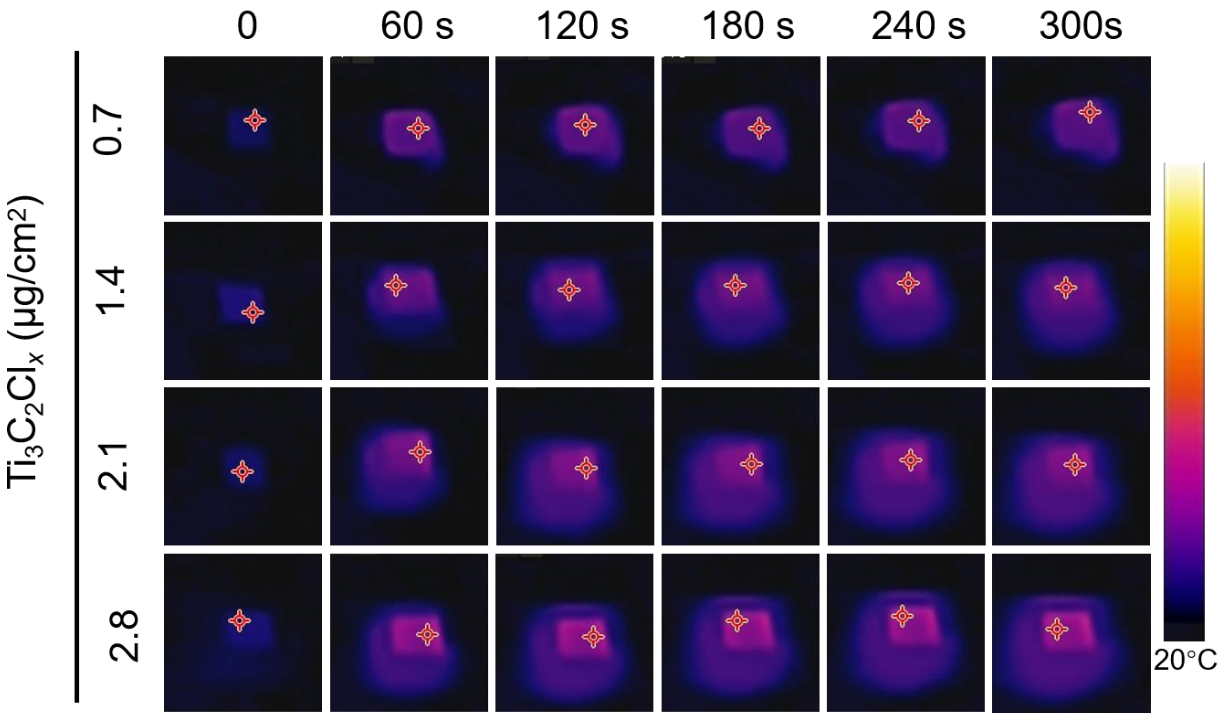

Fig. S59.

Infrared thermal images of woundplast loaded with reduced $Ti_3C_2Cl_x$ at different concentrations under 808 nm laser irradiation with a power density of 1 W/cm^2.

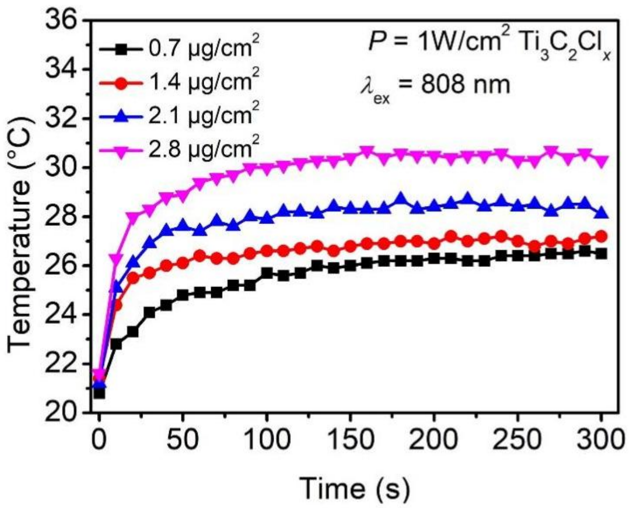

Fig. S60.

Photothermal heating curves of woundplast loaded with reduced $Ti_3C_2Cl_x$ at different concentrations under 808 nm laser irradiation with a power density of 1 W/cm^2.

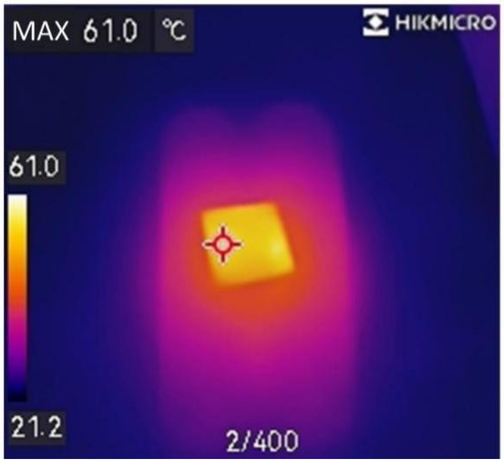

Fig. S61.
Infrared imaging image of woundplast (loaded with reduced Ti_3C_2 (1:8, 550°C) nanosheets) with bacteria after irradiations with 1W/cm^2 808nm laser.

Table S1. Synthesis recipes for different products.

Molar ratio	Molten salt	Temperature	Time	Denoted as
Ti_3AlC_2:$CdCl_2$=1:3	NaCl-KCl	700℃	5h	$Ti_3C_2Cl_x$
$Ti_3C_2Cl_x$:Na=1:0	LiCl-KCl	500℃	5h	Annealed $Ti_3C_2Cl_x$
$Ti_3C_2Cl_x$:Na=1:2	LiCl-KCl	500℃	5h	Reduced Ti_3C_2 (1:2, 500℃)
$Ti_3C_2Cl_x$:Na=1:4	LiCl-KCl	500℃	5h	Reduced Ti_3C_2 (1:4, 500℃)
$Ti_3C_2Cl_x$:Na=1:8	LiCl-KCl	500℃	5h	Reduced Ti_3C_2 (1:8, 500℃)
$Ti_3C_2Cl_x$:Na=1:12	LiCl-KCl	500℃	5h	N/A
$Ti_3C_2Cl_x$:Na=1:8	LiCl-KCl	550℃	5h	Reduced Ti_3C_2 (1:8, 550℃)
$Ti_3C_2Cl_x$:Na=1:10	LiCl-KCl	550℃	5h	Reduced Ti_3C_2 (1:10, 550℃)
$Ti_3C_2Cl_x$:Na=1:8	LiCl-KCl	600℃	5h	N/A

Table S2. XPS peak fitting results of pristine $Ti_3C_2Cl_x$.

Region	BE(eV)	FWHM(eV)	Fraction	Assigned to
Ti $2p_{3/2}(2p_{1/2})$	455.17(461.07)	1.2(1.5)	42.3	Ti(I)-C
	456.22(462.28)	1.3 (1.3)	23.2	Ti(II)-C
	457.4(463.38)	1.57(1.5)	16.3	Ti(III)-C
	459.11(465.21)	2.2(2.5)	18.2	TiO_x
Al $2p_{3/2}(2p_{1/2})$	75.14(75.58)	2.09(2.09)	100	Al-O
Cl $2p_{3/2}(2p_{1/2})$	199.5(201.1)	1.56(1.44)	100	Cl-Ti
C 1s	282.27	0.97	36.6	C-Ti
	284.8	1.99	63.4	C-C

Table S3. XPS peak fitting results of annealed $Ti_3C_2Cl_x$.

Region	BE(eV)	FWHM(eV)	Fraction	Assigned to
Ti $2p_{3/2}(2p_{1/2})$	455.17(461.27)	1.09(1.09)	34.2	Ti(I)-C
	456.22(462.21)	1.15 (1.18)	18.8	Ti(II)-C
	457.41(463.41)	1.34(1.37)	13.5	Ti(III)-C
	459.11(465.21)	1.53(1.67)	33.5	TiO_x
Cl $2p_{3/2}(2p_{1/2})$	199.15(200.75)	1.04(1.18)	100	Cl-Ti
C 1s	282.02	0.88	10.5	C-Ti
	284.8	1.65	59.8	C-C
	286.37	2.04	21.5	C-O
	288.79	1.48	8.2	C=O

Table S4. XPS peak fitting results of reduced Ti_3C_2 (1:2, 500°C).

Region	BE(eV)	FWHM(eV)	Fraction	Assigned to
Ti $2p_{3/2}(2p_{1/2})$	455.15(461.26)	1.2(1.2)	24.5	Ti(I)-C
	456.17(462.25)	1.34 (1.24)	20.1	Ti(II)-C
	457.34(463.40)	1.49(1.39)	16.2	Ti(III)-C
	459.22(464.84)	2.55(2.55)	39.2	TiO_x
Cl $2p_{3/2}(2p_{1/2})$	199.12(200.74)	2.08(2.08)	100	Cl-Ti
C 1s	282.17	1.21	19.5	C-Ti
	284.8	1.65	80.5	C-C
O 1s	531.25	1.77	56.49	O-Ti
	532.55	1.93	43.51	OH-Ti

Table S5. XPS peak fitting results of reduced Ti_3C_2 (1:8, 500°C).

Region	BE(eV)	FWHM(eV)	Fraction	Assigned to
Ti $2p_{3/2}(2p_{1/2})$	455.07(461.03)	1.25(1.2)	24.2	Ti(I)-C
	456.03(462.1)	1.4 (1.3)	19.1	Ti(II)-C
	457.19(463.32)	1.6(1.4)	16.0	Ti(III)-C
	459.05(464.99)	2.4(1.96)	40.7	TiO_x
Cl $2p_{3/2}(2p_{1/2})$	199.12(200.72)	2.39(2.39)	100	Cl-Ti
C 1s	281.97	1.04	21.7	C-Ti
	284.8	2.1	78.3	C-C
O 1s	531.31	2.30	68	O-Ti
	532.64	2.11	32	OH-Ti

Table S6. XPS peak fitting results of reduced Ti_3C_2 (1:8, 550°C).

Region	BE(eV)	FWHM(eV)	Fraction	Assigned to
Ti $2p_{3/2}(2p_{1/2})$	454.97(460.94)	1.34(1.34)	31.6	Ti(I)-C
	455.83(461.83)	1.52 (1.52)	18.7	Ti(II)-C
	456.9(463.07)	1.74(1.74)	18.9	Ti(III)-C
	459.02(465.17)	2.5(2.0)	30.8	TiO_x
Cl $2p_{3/2}(2p_{1/2})$	199.29(200.99)	1.91(1.8)	100	Cl-Ti
C 1s	282.1	1.1	25.1	C-Ti
	284.8	2.26	74.9	C-C
O 1s	531.21	2.39	80.3	O-Ti
	532.62	2.2	19.7	OH-Ti

Table S7. XPS peak fitting results of $Ti_3C_2Cl_x$ nanosheets.

Region	BE(eV)	FWHM(eV)	Fraction	Assigned to
Ti $2p_{3/2}(2p_{1/2})$	455.17(461.37)	1.11(1.21)	13.2	Ti(I)-C
	456.19(462.39)	1.2(1.4)	13.3	Ti(II)-C
	457.47(463.67)	1.4(1.3)	17.5	Ti(III)-C
	459.41(465.19)	1.98(1.92)	56	TiO_x
C 1s	286.31	2.02	10	C-O
	282.40	1.14	9.5	C-Ti
	288.28	1.39	6.2	C=O
	284.8	1.9	74.3	C-C
Cl $2p_{3/2}(2p_{1/2})$	199.53(201.13)	1.44(1.33)	100	Cl-Ti

Table S8. XPS peak fitting results for reduced Ti_3C_2 nanosheets (1:8, 550°C) prepared in ascorbic acid solution.

Region	BE(eV)	FWHM(eV)	Fraction	Assigned to
Ti $2p_{3/2}(2p_{1/2})$	454.97(461.05)	1.25(1.24)	23.3	Ti(I)-C
	455.83(461.85)	1.4(1.3)	20.6	Ti(II)-C
	456.9(463.1)	1.64(1.5)	18.9	Ti(III)-C
	458.9(465.05)	2.55(2.41)	37.2	TiO_x
C 1s	282.14	1.27	13.2	C-Ti
	284.8	1.76	72.2	C-C
	286.70	1.76	14.6	C-O

Table S9. XPS peak fitting results for reduced Ti_3C_2 nanosheets (1:8, 550℃) treated with deionized water for 24h.

Region	BE(eV)	FWHM(eV)	Fraction	Assigned to
Ti $2p_{3/2}(2p_{1/2})$	454.97(461.12)	1.02(1.12)	19.9	Ti(I)-C
	455.77(461.9)	1.21 (1.11)	18.5	Ti(II)-C
	456.89(462.92)	1.55(1.34)	19.4	Ti(III)-C
	458.99(464.77)	2.68(2.68)	42.2	TiO_x
C 1s	286.21	2.13	23.4	C-O
	284.8	1.43	46.7	C-C
	282.08	0.86	29.9	C-Ti
O 1s	531.30	2.01	77.6	O-Ti
	532.60	2.5	22.4	OH-Ti

Table S10. XPS peak fitting results of reduced Ti_3C_2 nanosheets (1:8, 550°C) prepared without ascorbic acid solution.

Region	BE(eV)	FWHM(eV)	Fraction	Assigned to
Ti $2p_{3/2}(2p_{1/2})$	455.28(461.73)	1.29(1.29)	11.6	Ti(I)-C
	456.66(462.82)	1.32 (1.32)	14.7	Ti(II)-C
	457.78(464.09)	1.13(1.61)	14.9	Ti(III)-C
	459.41(465.25)	1.93(1.93)	58.8	TiO_x
C 1s	286.37	1.38	10.7	C-O
	284.8	1.65	89.3	C-C
O 1s	531.20	2.68	64.1	O-Ti
	532.74	1.44	35.9	OH-Ti

Table S11. EXAFS data fitting results of Ti of reduced Ti_3C_2 (1:8, 550°C) nanosheets

Sample	Path	CN[a]	R(Å)[b]	σ^2 (Å^2)[c]	ΔE_0(eV)[d]	R factor
Ti K-edge (S_0^2=0.617)						
Ti foil	Ti-Ti	12*	2.91±0.01	0.0073	4.7	0.0058
Reduced Ti_3C_2	Ti-O	0.94±0.23	2.05±0.01	0.0020	7.5	0.0052
	Ti-Ti	5.51±0.20	2.96±0.01	0.0031	-5.4	
	Ti-C	3.02±0.51	2.17±0.01	0.0060	1.7	
	Ti-O/C-Ti	4.81±0.18	3.04±0.01	0.0010	-6.7	

[a]CN, coordination number; [b]R, the distance between absorber and backscatter atoms; [c]σ^2, the Debye Waller factor value; [d]ΔE_0, inner potential correction to account for the difference in the inner potential between the sample and the reference compound; R factor indicates the goodness of the fit. S_0^2 was fixed to 0.617, according to the experimental EXAFS fit of Ti foil by fixing CN as the known crystallographic value. * This value was fixed during EXAFS fitting. Fitting conditions: k range: 3.0–12.0; R range: 1.0–3.0; fitting space: R space; k-weight = 2. A reasonable range of EXAFS fitting parameters: CN > 0; σ^2 > 0 Å^2; $|\Delta E_0|$ < 10 eV.

Table S12. **Electronic information of different MXenes from the Hall effect test**

Sample	Carrier type	Carrier Areal density (cm^{-2})	Carrier volume density(cm^{-3})	Resistivity (Ω·cm)	Conductivity (S/cm)	Hall coefficient (cm^3/C)	Carrier mobility (cm^2/Vs)
$Ti_3C_2Cl_x$	n	9.66221E19	1.61037E21	0.00223	448.43	-0.00387	1.73951
Annealed $Ti_3C_2Cl_x$ (500°C)	n	1.01456E20	1.69094E21	0.00197	507.61	-0.00369	1.86928
Reduced Ti_3C_2(1:2, 500°C)	n	1.12883E20	1.88139E21	0.00141	709.22	-0.00332	2.35762
Reduced Ti_3C_2(1:4, 500)	n	1.12418E20	1.87364E21	0.00137	729.93	-0.00333	2.43916
Reduced Ti_3C_2(1:8, 500°C)	n	2.13694E20	3.56157E21	5.39657E-4	1853.03	-0.00175	3.24657
Reduced Ti_3C_2(1:8, 550°C)	n	4.75857E20	7.93096E21	1.72075E-4	5811.42	-7.8679E-4	4.57236
Reduced Ti_3C_2(1:10, 550°C)	n	1.67013E20	2.78355E21	7.31941E-4	1366.22	-0.00224	3.06273

Table S13. **Mulliken charge of different MXenes**

Material	Inner Ti	Outer Ti	C	Cl	Total charge of Ti_3C_2
$Ti_3C_2Cl_x$	0.448	0.267	-0.425	-0.066	0.132
Ti_3C_2	0.331	0.201	-0.366		0
Ti_3C_2-1	0.256	-0.24	-0.388		-1
Ti_3C_2-2	-0.377	-0.631	-0.18		-2

Table S14. **Summary of photothermal conversion efficiency of different MXene-based nanosystems.**

Material names	PTCE
$Ti_3C_2T_x$-DNA	24.4
Mo_2C-PVA	24.5
Nb_2C-aOPN	28.51
$Ti_3C_2T_x$@EGCG	29.2
Fe-Ti_3C_2	29.3
FA@MXene/CuO_2/GA	29.4
V_4C_3	29.79
$Ti_3C_2T_x$	30.6
$Ti_3C_2T_x$	30.7
TC@Ch-MS	31.2
$Ti_3C_2T_x$-Pt	31.78
$Ti_3C_2T_x$	32.3
$W_{1.33}C$	32.5
$Ti_3C_2T_x$-DOX/PEG	33.34
Ti_3C_2-CoNWs	34
$Ti_3C_2T_x$@Au	34.3
MnO_x/Ta_4C_3	34.9
$Ti_3C_2T_x$/agarose hydrogel	35
$Ti_3C_2T_x$	35.02
$Bi_2S_3/Ti_3C_2T_x$	35.43
Ti_3CN	36.5
CeO_2/Nb_2C	38
CP-MX-Z(BTC)@CS	38.1
$Ti_3C_2T_x$	38.5
Ti_3C_2-HPG	38.66
Ti_3C_2-PVP@DOXjade	40
$Ti_3C_2T_x$	40.28
Mn-Ti_3C_2@PEG	41.3
$Ti_3C_2T_x$/PDA	42.3
$Ti_3C_2T_x$-Au	43.40
FA_Au@c-Ti_3C_2	43.51
Ta_4C_3	44.7
HE-Mxene	44.8
MXene/MPN/Ag	46.6
$Ti_3C_2T_x$/Fe MOFs	51.9
$Ti_3C_2T_x$-Au	55
BiOI@Bi_2S_3/Ti_3C_2	57.8
Ti_3C_2-DOX	58.3
$Ti_3C_2Cl_x$	59.09
Ti_3C_2/Cu_2O	63.69
Reduced Ti_3C_2	91.66

Movie S1.

Photothermal comparison of woundplasts loaded with $Ti_3C_2Cl_x$ and reduced Ti_3C_2 nanosheets.

Movie S2.

Photothermal comparison of woundplasts loaded with gold nanoparticles and reduced Ti_3C_2 nanosheets.
